# Identifying and Targeting Prediction of the PI3K-AKT Signaling Pathway in Drug-Induced Thrombocytopenia in Infected Patients Receiving Linezolid Therapy: A Network Pharmacology-Based Analysis

**DOI:** 10.1155/2022/2282351

**Published:** 2022-10-15

**Authors:** Jiao Xie, Hongli Chen, Youjia Li, Chenwei Liu, Xiaowei Zheng, Siping Feng, Haitao Wang

**Affiliations:** ^1^Department of Pharmacy, The Second Affiliated Hospital of Xi'an Jiaotong University, Xi'an, China; ^2^Department of Hematology, The Second Affiliated Hospital of Xi'an Jiaotong University, Xi'an, China; ^3^Department of Pharmacy, Xi'an No.1 Hospital, Xi'an, China; ^4^Department of Pharmacy, Yan'an People's Hospital, Yan'an, China

## Abstract

The pharmacological mechanisms underlying the adverse effects of linezolid on thrombocytopenia have not been conclusively determined. This network pharmacology study aimed at investigating the potential pharmacological mechanisms of linezolid-induced adverse reactions in thrombocytopenia. In this study, target genes for linezolid and thrombocytopenia were compared and analyzed. Overlapping thrombocytopenia-associated targets and predicted targets of linezolid were imported to establish protein-protein interaction networks. Gene Ontology and the Kyoto Encyclopedia of Genes and Genome pathway enrichment analyses were performed to determine the enriched biological terms and pathways. The mechanisms involved in linezolid-induced thrombocytopenia were established to be associated with various biological processes, including T cell activation, peptidyl serine modification, and peptidyl serine phosphorylation. The top five relevant protein targets were obtained, including ALB, AKT1, EGFR, IL6, and MTOR. Enrichment analysis showed that the targets of linezolid were positively correlated with T cell activation responses. The mechanism of action of linezolid was positively correlated with the PI3K-AKT signaling pathway and negatively correlated with the Ras signaling pathway. We identified the important protein targets and signaling pathways involved in linezolid-induced thrombocytopenia in anti-infection therapy, providing new information for subsequent studies on the pathogenesis of drug-induced thrombocytopenia and potential therapeutic strategies for rational use of linezolid in clinical settings.

## 1. Introduction

Linezolid, the first oxazolidinone antibacterial drug to be introduced on the market, is a broad-spectrum antibacterial agent with 100% oral bioavailability. *In vivo*, linezolid rapidly achieves effective blood concentrations with low protein binding and favorable drug distribution [1]. A recent meta-analysis of randomized clinical trials showed that the efficacy of linezolid for treatment of Gram-positive bacterial pneumonia was comparable to that of controls [2]. Linezolid-associated nephrotoxic effects are lower than those of vancomycin; however, with widespread clinical uses of linezolid, there are concerns about its platelet suppression effects, anemia induction, and other hematologic adverse effects [3–6]. Due to differences in design protocols and anemia definitions among studies, incidences of reported linezolid-associated adverse events have been shown to range from 14.1% to 64.7% [7–9]. When administered for an extended period of time, linezolid is associated with a high rate of adverse events, the most common of which is anemia. The mechanisms involved in linezolid-induced anemia have yet to be fully clarified. Severe drug-induced anemia is associated with multiple mechanisms. Therefore, through a network pharmacology approach, we investigated the mechanisms of linezolid-induced thrombocytopenia.

## 2. Materials and Methods

### 2.1. Screening for Potential Targets of Linezolid

The SMILES ID of linezolid was obtained from the PubChem database [10] (https://pubchem.ncbi.nlm.nih.gov/), imported into the SwissTargetPrediction database [11] (http://www. swisstargetprediction.ch/) and the SEA database [12] (http://sea.bkslab.org/) to obtain the corresponding compound targets, followed by the GeneCards database [13] (https://www.genecards.org/) to retrieve the compound targets of the drug. Targets of the SwissTargetPrediction database were selected with a probability >0 score for inclusion. To obtain the final potential targets of linezolid, the obtained targets were adjusted while duplicates were eliminated by the UniProt database (https://www.uniprot.org/).

### 2.2. Screening for Potential Targets of Thrombocytopenia

Human gene searches were performed in the GeneCards database, the NCBI database [15], the OMIM database [16] (https://www. omim.org/), and the DisGeNET database [17] (https://www.disgenet.org/) using the keyword “Thrombocytopenia” for human gene searches. All identified targets were de-duplicated and integrated to construct a database of thrombocytopenia-related targets.

### 2.3. Potential Linezolid Targets for Adverse Reactions Associated with Thrombocytopenia

Target genes for the action of linezolid and thrombocytopenia were matched and the results presented by a Venn diagram (Venny software version 2.1).

### 2.4. Establishment and Analysis of Overlapping Target Networks

Overlapping target gene data for linezolid and thrombocytopenia were imported into the STRING database (https://string-db.org/cgi/input.pl) for protein-protein interaction (PPI) analysis. The PPI network results were obtained by loading the PPI data while network plots of overlapping targets were prepared using the Cytoscape software (version 3.8.0). Topological data analysis was performed using the NetworkAnalyzer tool, using degree as the reference standard and sorted by degree. Genes whose scores were greater than the average score were selected as key targets. In this study, screening of key genes was performed via MCODE analysis. After importing the PPI network data, gene clustering analysis and core target screening were implemented using the MCODE module.

### 2.5. Pathway Enrichment Analysis

The Kyoto Encyclopedia of Genes and Genomes (KEGG) pathway and Gene Ontology (GO) enrichment analyses were performed to systematically analyze the functions of potential target genes for linezolid-induced thrombocytopenia in terms of a network of genes and molecules.

## 3. Results

### 3.1. Results of Target Screening of Linezolid and Thrombocytopenia

After removing repeated targets from SwissTargets and SEA databases, 183 linezolid targets were obtained. GenoCards, NCBI, OMIM, and DisGeNET were used to collect the thrombocytopenia target data. We combined data from four databases and removed duplicate items to obtain 2068 thrombocytopenia-related targets.

### 3.2. Potential Targets for Linezolid-Induced Thrombocytopenia

**Figure 1:**
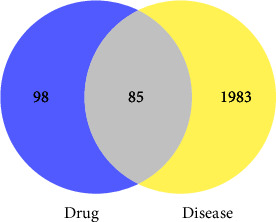
Potential targets for linezolid-induced thrombocytopenia.

After crossover analysis of the targets of linezolid and thrombocytopenia-related targets, we obtained 85 overlapping targets, which may be responsible for linezolid-induced thrombocytopenia (Figure 1 and Table 1).

### 3.3. PPI Network Analysis

**Figure 2:**
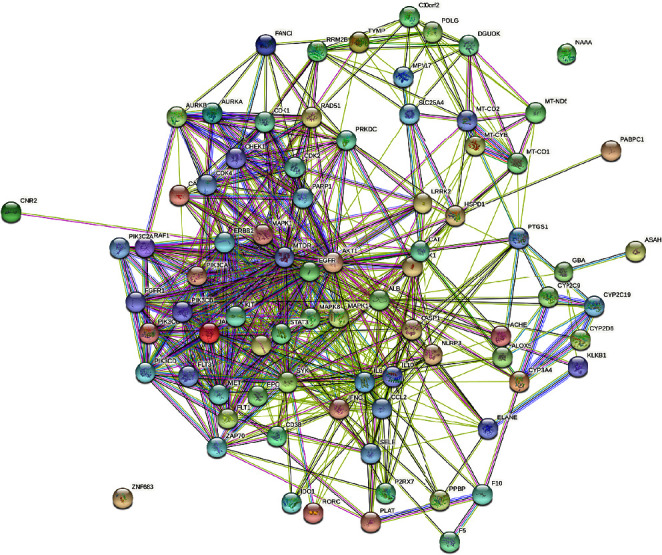
The PPI network of 85 drug-disease interaction targets according to the STRING website.

**Figure 3:**
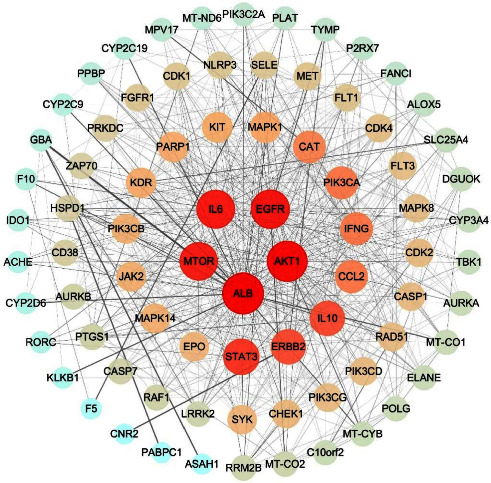
The PPI network analysis of 85 drug-disease interaction targets according to the Cytoscape software.

The PPI network derived from analysis of 85 drug-disease interaction targets was established using the STRING website (Figure 2). The PPI network in Figure 3 was created using the Cytoscape software, where node sizes and color were adjusted based on the degree value. Nodes with larger dimensions and darker colors indicate larger degree values, whereas lines from thick to thin indicate edge betweenness from large to small. Based on our analyses, the top five targets are ALB, AKT1, EGFR, IL6, and MTOR.

### 3.4. Results  of Topological  Data and MCODE Cluster Analyses

**Figure 4:**
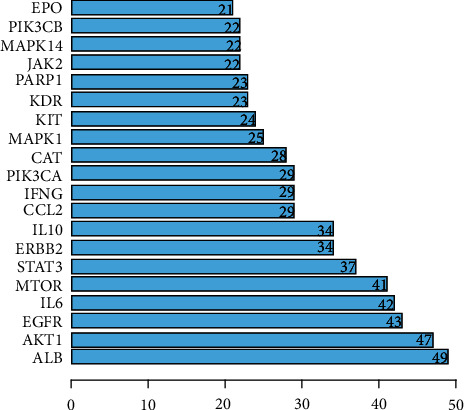
Plotting of the top 20 targets from topological data analysis.

Through degree sorting, genes with scores better than the average were selected as key targets in the topological data analysis. Thirty-two key targets were screened, and the top 20 targets are plotted in Figure 4. The horizontal coordinates are the degree values of different targets. In this study, screening of key genes was also performed by MCODE analysis. After importing the constructed PPI network, the MCODE module was used to analyze gene clusters and to screen the core targets. We identified four gene clusters and four core genes (MAPK14, PARP1, MAPK8, and POLG) (Supplementary Table 1 presents findings from MCODE cluster analysis).

### 3.5. Drug-Disease Target Network Development

**Figure 5:**
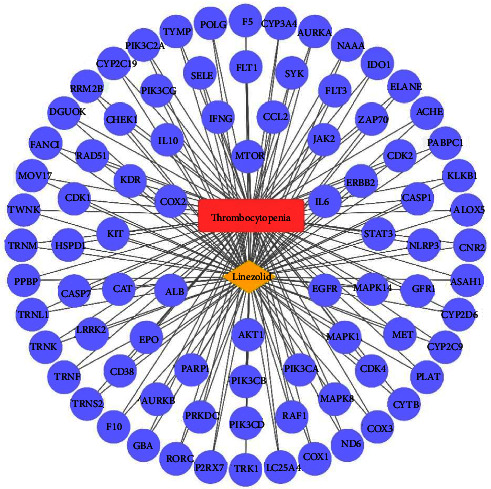
Plotting of drug-disease target network for linezolid-induced thrombocytopenia.

A drug-disease-relevant target network diagram was constructed based on inclusion of potentially relevant targets to understand the complex interplay among linezolid, thrombocytopenia, and corresponding targets (Figure 5).

### 3.6. Enrichment Analysis of Target Pathways

**Figure 6:**
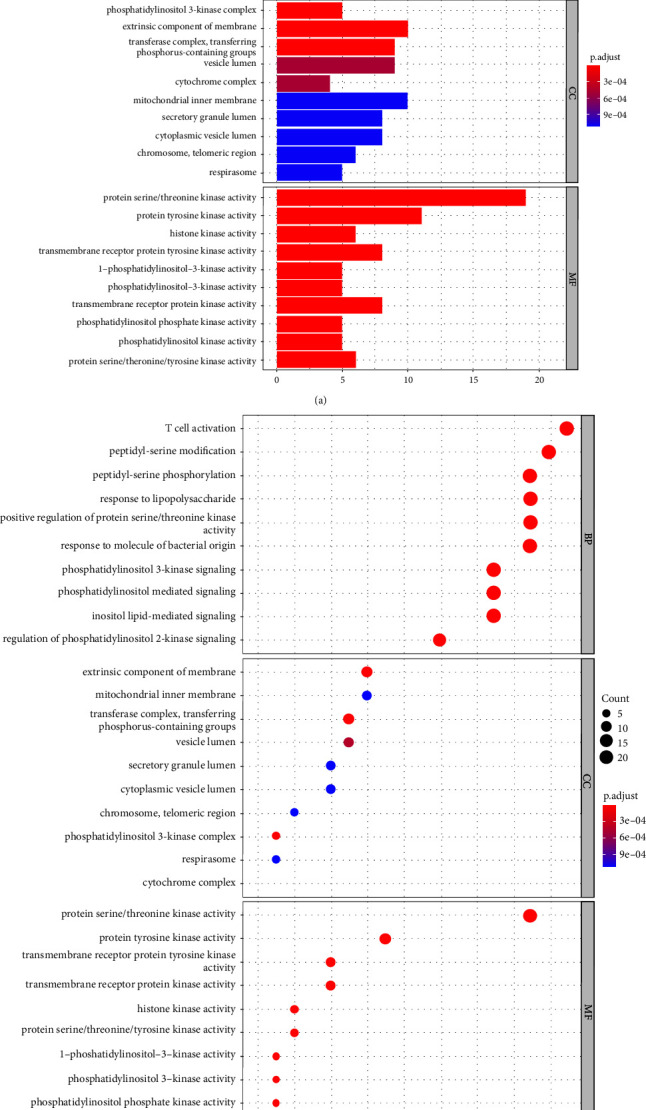
(a) Bar plot of GO enrichment analysis. (b) Bubble plot of GO enrichment analysis.

Enriched GO terms were categorized by biological processes (BP), molecular functions (MF), and cells components (CC) for targets relevant to linezolid and thrombocytopenia. A total of 1481 GO terms were obtained (55 CC terms, 1359 BP terms, and 67 MF terms). We selected the top 10 statistically significant GO information terms; moreover, in GO enrichment circles, the circle size or line length represents number of genes enriched, while the color represents prominence of enrichment. Several biological processes were associated with linezolid-induced thrombocytopenia, including T cell activation, peptidyl-serine modification, and peptidyl-serine phosphorylation. Various cellular components are also involved, including the extrinsic component of the membrane, mitochondrial inner membrane, and transferase complex transferring phosphorus-containing groups. Protein serine/threonine kinase activity, protein tyrosine kinase activity, and transmembrane receptor protein tyrosine kinase activity were the associated molecular functions (Figures 6(a) and 6(b)).

**Figure 7:**
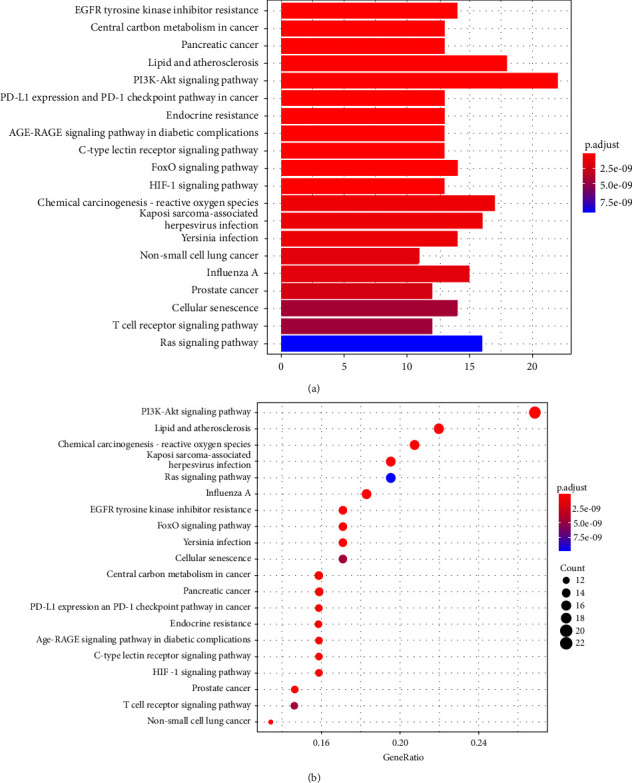
(a) Bar plot of KEGG pathway enrichment analysis. (b) Bubble plot of KEGG pathway enrichment analysis.

In Figures 7(a) and 7(b), we selected the top 20 most highly correlated KEGG pathway enrichment items to be shown as bubble and bar plots. The length of the bar and size of the circle in the plots reflects the number of genes that were enriched in KEGG, while color represents the significance of gene enrichment. The targets were found to be markedly enriched in multiple pathways, including the PI3K-AKT, Ras, FoxO, C-type lectin receptor, and the HIF-1 signaling pathways. It is shown that linezolid has positive associations with reactions to T cell activation. Moreover, the effects of linezolid were positively correlated with the PI3K-AKT signaling pathway and adversely correlated with the Ras signaling pathway.

**Figure 8:**
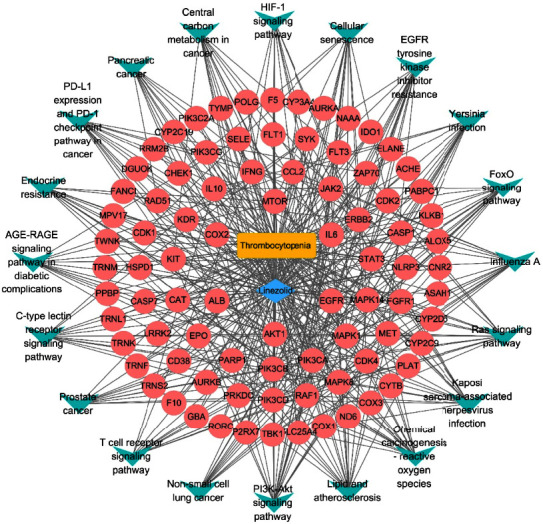
Drug-target-pathway network for linezolid-induced thrombocytopenia.

We plotted the drug-target-pathway network to visualize the characteristics of linezolid-induced thrombocytopenia during infection treatment. In Figure 8, the blue diamond represents the drug, pink circles represent potential targets of linezolid-induced thrombocytopenia, green arrows are the top 20 most significant pathways, while the yellow square represents the disease.

## 4. Discussion

Platelets are among the most important cells in the body and play a role in accelerating blood clotting and hemostasis. Impaired megakaryocyte maturation results in decreased platelet production, leading to symptoms such as skin petechiae, the gums, gastrointestinal bleeding, and in severe cases, life-threatening brain hemorrhage. The mechanisms involved in linezolid-induced anemia have not been fully understood, while multiple mechanisms are involved in development of linezolid-induced thrombocytopenia. An 83-year-old man was administered with linezolid for *Staphylococcus aureus* treatment. After the initiation of linezolid therapy, he developed progressive anemia. During the linezolid-administration period, his hemoglobin levels were 5.7 g/dL, reticulocyte percentage was 0.36%, while his white blood cell and platelet counts were unchanged. Bone marrow examination revealed a significant reduction in erythropoiesis with cytoplasmic vacuolation of erythroblasts, suggesting that the anemia caused by linezolid may have been due to myelosuppression [18]. The potential hematological side effects associated with linezolid, including pure red aplastic anemia are a public health concern. Wang et al. evaluated the levels of reactive oxygen species, malondialdehyde, and cholesterol as well as the activities of antioxidant enzymes in blood samples after linezolid treatment. They found that serum levels of reactive oxygen species and malondialdehyde were significantly elevated while superoxide dismutase and catalase levels were suppressed in the thrombocytopenic group, relative to the normal platelet count group. These findings suggest that oxidative damage might be the underlying mechanism of thrombocytopenia in patients receiving prolonged linezolid treatment [19]. In an *in vivo* study, Tajima et al. found elevated levels of myosin light chain 2 (MLC2) phosphorylation in mature megakaryocytes affected by linezolid. Biologically, MLC2 regulates platelet maturation [20]. Linezolid-induced thrombocytopenia may also result from MLC2 phosphorylation, which blocks the maturation of megakaryocytes into platelets.

In this study, we found that linezolid-induced thrombocytopenia is associated with the PI3K-AKT pathway. Our conclusion is supported by the latest findings, which showed that the PI3K-AKT pathway is a key pathway through which the macrophage M2 subtype promotes megakaryocyte maturation [21]. Platelets are produced by migration and directed differentiation of hematopoietic stem cells into megakaryocytes in the bone marrow microenvironment. Megakaryocyte maturation and platelet production are strictly regulated by the bone marrow microenvironment. *In vitro* and in a macrophage-specific PI3K-knockdown mouse model, genetic knockdown of the PI3K-AKT pathway impaired the ability of macrophages to support megakaryopoiesis, suggesting an important role of the PI3K-AKT pathway in regulating macrophage M2 megakaryopoiesis. In addition, the TGF-*β* released by M2 macrophages promotes megakaryopoiesis by upregulating the JAK2/STAT5 and MAPK/ERK pathways. In this study, the core genes identified by MCODE analysis contained MAPK8 and MAPK14. Our findings reveal the significance of the PI3K-AKT pathway in linezolid-induced thrombocytopenia and provide a basis for in-depth studies on potential treatment targets that promote megakaryopoiesis.

A previous case report concluded that linezolid causes thrombocytopenia via immunosuppression. By performing a bone marrow biopsy for a linezolid-administered patient with associated thrombocytopenia, Bernstein et al. showed that linezolid-induced thrombocytopenia is not associated with myelosuppression or thrombocytopoiesis, and adequate, normal megakaryocytes were detected. In contrast, after the administration of immunoglobulin therapy, the rate of platelet count decline slowed in this patient, suggesting drug-induced immune-mediated thrombocytopenia[22]. We found that the mechanisms of action involved in linezolid-induced thrombocytopenia are associated with various biological processes, including T cell activation. Immune thrombocytopenia has traditionally been considered to be a B-cell-mediated disorder, as antiplatelet antibodies are detected in most patients. The nature of autoantigens, the apparent processes of isotype switching, and affinity maturation of antiplatelet antibodies suggest that for B cells to induce an antiplatelet immune response, they require the support of self-functioning CD4^+^ T cells. The pathogenesis of immune thrombocytopenia can be traced to imbalanced Th1 and Th2 subsets of CD4^+^ T cells. Numerous subsets of these cells have been described, including Th17, Th9, Th22, as well as T follicular helper and regulatory T cells[23]. Elucidation of the mechanisms of action of linezolid through immune-mediated responses to platelet clearance will inform on the roles of different immune cells and different targets for thrombocytopenia treatment.

Based on the abovementioned studies, linezolid-induced thrombocytopenia may be caused by multiple mechanisms that may function together. Since most diseases today are defined by phenotypes instead of mechanisms, we are hardly aware of the mechanism of any disease and treat the symptoms with low precision consistently [24, 25]. The concept of network pharmacology refers to pharmacological treatment that involves the combination of drugs that target the causal disease module or signaling network and acts synergistically on molecules within the network [26–28]. Network-based approaches offer the development of robust multilayer computational networks integrating related data may help to investigate complex biological pathways altered by drug treatment or to better understand the complex biological mechanisms that contribute to several diseases [29, 30].

Mechanistically, linezolid-associated thrombocytopenia may be due to different mechanisms, which should be further investigated. This study has some limitations. Not all database information was included in this study, implying that some targets may have been missed during screening. In addition, the time of onset and change in action of linezolid-induced thrombocytopenia could not be interpreted and analyzed in this study, and the effects of drug dose on disease onset could not be analyzed.

## 5. Conclusions

The mechanisms involved in linezolid-induced thrombocytopenia were established to be associated with various biological processes, including T cell activation, peptidyl serine modification, and peptidyl serine phosphorylation. The top five relevant protein targets were obtained, including ALB, AKT1, EGFR, IL6, and MTOR. Enrichment analysis showed that the targets of linezolid were positively correlated with T cell activation responses. The mechanism of action of linezolid was positively correlated with the PI3K-AKT signaling pathway and negatively correlated with the Ras signaling pathway. We identified key proteins and metabolic pathways associated with linezolid-induced thrombocytopenia during anti-infection therapy, which forms the basis for subsequent studies on the pathogenesis of drug-induced thrombocytopenia and identification of potential therapeutic strategies.

## Figures and Tables

**Table 1:** The related target genes of linezolid-induced thrombocytopenia.

Number	Gene symbol	Number	Gene symbol	Number	Gene symbol
1	CYP3A4	31	ND6	61	TBK1
2	CYP2D6	32	COX3	62	CASP7
3	CYP2C9	33	CYTB	63	AKT1
4	F5	34	TRNS2	64	CASP1
5	POLG	35	F10	65	PABPC1
6	TYMP	36	STAT3	66	FLT1
7	ALB	37	GBA	67	FGFR1
8	IL6	38	MAPK14	68	FLT3
9	COX2	39	PRKDC	69	ACHE
10	PIK3C2A	40	RORC	70	ELANE
11	CYP2C19	41	KDR	71	CDK2
12	IFNG	42	PIK3CB	72	IDO1
13	CCL2	43	CHEK1	73	ERBB2
14	IL10	44	P2RX7	74	EGFR
15	RRM2B	45	SYK	75	MTOR
16	SLC25A4	46	MET	76	AURKB
17	DGUOK	47	LRRK2	77	CDK1
18	FANCI	48	RAF1	78	CDK4
19	MPV17	49	JAK2	79	NAAA
20	TWNK	50	PARP1	80	AURKA
21	TRNM	51	ASAH1	81	ZAP70
22	PPBP	52	CNR2	82	SELE
23	NLRP3	53	PIK3CD	83	KIT
24	TRNL1	54	PIK3CG	84	MAPK8
25	TRNK	55	PIK3CA	85	RAD51
26	TRNF	56	ALOX5		
27	CAT	57	PLAT		
28	HSPD1	58	KLKB1		
29	EPO	59	MAPK1		
30	COX1	60	CD38		

## Data Availability

The data used to support the findings of this study are available from the corresponding author upon request.

## Supplementary Materials

Supplementary Materials Supplementary Table 1 and experimental dataset are provided for MCODE cluster analysis. Supplementary data files for all the figures are also provided in the supplementary materials.
