# Supplementary material for: Identifying and Targeting Prediction of the PI3K-AKT Signaling Pathway in Drug-Induced Thrombocytopenia in Infected Patients Receiving Linezolid Therapy: A Network Pharmacology-Based Analysis
Source: J Healthc Eng. 2022 Oct 15;2022:2282351. doi: 10.1155/2022/2282351 (PMC9588367; doi:10.1155/2022/2282351)
Supplement: Supplementary Materials — Supplementary Table 1 and experimental dataset are provided for MCODE cluster analysis. Supplementary data files for all the figures are also provided in the supplementary materials. [file 2282351.f1.zip › 1. Supplementary Table 1.docx]

**Supplementary Table 1 Results of MCODE cluster analysis.**

| **Cluster** | **Network** | **Nodes** | **Edges** | **Node IDs** |
| --- | --- | --- | --- | --- |
| 1 | 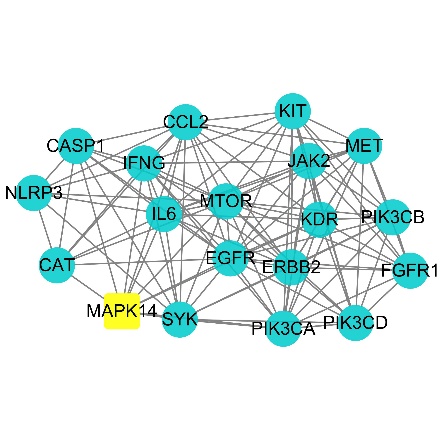 | 19 | 116 | MET, MAPK14, IFNG, JAK2, PIK3CB, PIK3CD, CCL2, KIT, EGFR, ERBB2, IL6, SYK, MTOR, CAT, PIK3CA, CASP1, KDR, NLRP3, FGFR1 |
| 2 | 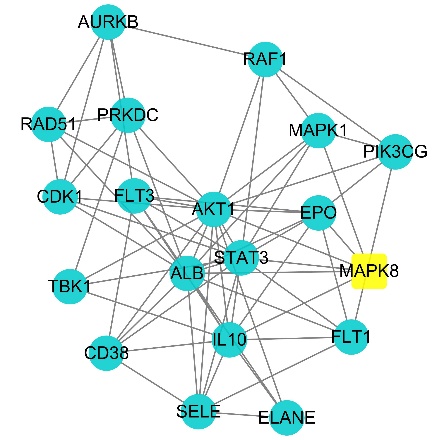 | 19 | 70 | FLT1, FLT3, MAPK8, TBK1, CDK1, PRKDC, AURKB, RAD51, CD38, MAPK1, AKT1, ALB, STAT3, ELANE, PIK3CG, IL10, EPO, SELE, RAF1 |
| 3 | 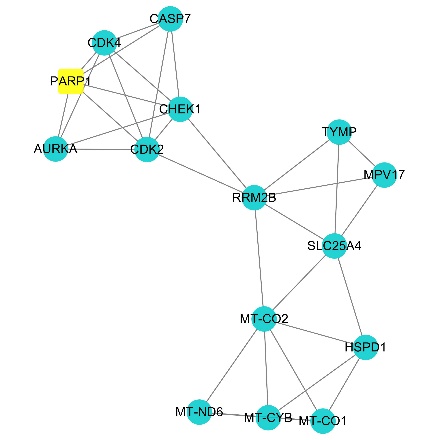 | 15 | 34 | MPV17, HSPD1, TYMP, MT-CO2, MT-CO1, AURKA, CASP7, MT-ND6, PARP1, SLC25A4, CDK2, MT-CYB, RRM2B, CDK4, CHEK1 |
| 4 | 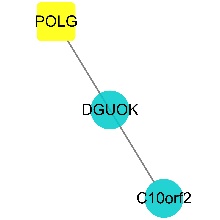 | 3 | 3 | C10orf2, DGUOK, POLG |
