# Supplementary material for: Identifying and Targeting Prediction of the PI3K-AKT Signaling Pathway in Drug-Induced Thrombocytopenia in Infected Patients Receiving Linezolid Therapy: A Network Pharmacology-Based Analysis
Source: J Healthc Eng. 2022 Oct 15;2022:2282351. doi: 10.1155/2022/2282351 (PMC9588367; doi:10.1155/2022/2282351)
Supplement: Supplementary Materials — Supplementary Table 1 and experimental dataset are provided for MCODE cluster analysis. Supplementary data files for all the figures are also provided in the supplementary materials. [file 2282351.f1.zip › Figure 4-topological data.pdf]

SUID	AverageShort	Betweenness(	ClosenessCer	ClusteringCo	Degree	Eccentrici	name
72.00	2.12	0.00	0.47	1.00	4.00	3.00	ACHE
73.00	1.67	0.04	0.60	0.43	28.00	3.00	CAT
75.00	1.37	0.20	0.73	0.30	49.00	2.00	ALB
77.00	1.41	0.09	0.71	0.35	47.00	3.00	AKT1
79.00	1.51	0.07	0.66	0.36	42.00	3.00	IL6
81.00	1.73	0.01	0.58	0.57	25.00	3.00	MAPK1
83.00	2.12	0.00	0.47	0.78	11.00	4.00	AURKA
85.00	1.72	0.02	0.58	0.51	29.00	3.00	CCL2
87.00	1.94	0.00	0.52	0.79	14.00	3.00	CD38
89.00	1.71	0.02	0.59	0.50	29.00	3.00	IFNG
91.00	1.81	0.01	0.55	0.66	22.00	3.00	MAPK14
94.00	1.90	0.00	0.53	0.65	18.00	3.00	FLT3
96.00	2.08	0.00	0.48	0.76	13.00	4.00	RAF1
98.00	1.86	0.00	0.54	0.73	21.00	3.00	EPO
100.00	1.88	0.00	0.53	0.69	18.00	3.00	CDK4
102.00	1.76	0.01	0.57	0.68	23.00	3.00	KDR
104.00	1.82	0.01	0.55	0.52	29.00	4.00	PIK3CA
106.00	1.60	0.03	0.62	0.47	37.00	3.00	STAT3
108.00	2.08	0.00	0.48	0.86	8.00	3.00	PIK3C2A
110.00	1.82	0.01	0.55	0.60	19.00	3.00	CDK2
112.00	1.62	0.05	0.62	0.46	34.00	3.00	ERBB2
114.00	1.47	0.07	0.68	0.40	43.00	3.00	EGFR
116.00	1.78	0.00	0.56	0.68	24.00	3.00	KIT
118.00	1.94	0.00	0.52	0.59	22.00	3.00	PIK3CB
121.00	1.92	0.01	0.52	0.50	12.00	3.00	LRRK2
123.00	1.92	0.01	0.52	0.68	15.00	3.00	PRKDC
125.00	2.09	0.00	0.48	0.82	10.00	4.00	TBK1
127.00	2.17	0.00	0.46	0.86	8.00	4.00	P2RX7
129.00	1.92	0.02	0.52	0.64	16.00	3.00	SELE
131.00	1.90	0.01	0.53	0.66	16.00	3.00	NLRP3
133.00	2.00	0.01	0.50	0.49	10.00	3.00	CYP3A4
135.00	2.00	0.00	0.50	0.66	19.00	4.00	PIK3CG
137.00	1.96	0.02	0.51	0.56	11.00	3.00	MT-CYB
139.00	1.55	0.03	0.64	0.46	41.00	3.00	MTOR
141.00	1.96	0.02	0.51	0.42	13.00	4.00	PTGS1
143.00	1.78	0.01	0.56	0.59	23.00	3.00	PARP1
145.00	1.96	0.00	0.51	0.76	13.00	3.00	CASP7
147.00	2.08	0.00	0.48	0.58	9.00	4.00	ALOX5
149.00	2.03	0.00	0.49	0.67	19.00	4.00	PIK3CD
151.00	1.82	0.02	0.55	0.54	19.00	3.00	RAD51
153.00	1.86	0.03	0.54	0.42	14.00	3.00	HSPD1
155.00	1.87	0.01	0.53	0.69	16.00	3.00	CDK1
157.00	1.88	0.00	0.53	0.76	18.00	3.00	MAPK8
160.00	1.85	0.01	0.54	0.57	20.00	3.00	CHEK1
162.00	1.92	0.00	0.52	0.84	16.00	3.00	FGFR1
164.00	1.63	0.03	0.61	0.44	34.00	3.00	IL10
166.00	1.87	0.00	0.53	0.70	19.00	3.00	CASP1
168.00	2.04	0.00	0.49	0.71	11.00	3.00	ELANE
171.00	2.10	0.00	0.48	0.50	8.00	3.00	PLAT
181.00	2.26	0.00	0.44	0.60	5.00	3.00	CYP2C9

185.00	2.31	0.00	0.43	0.67	3.00	3.00	KLKB1
191.00	1.90	0.00	0.53	0.76	17.00	3.00	FLT1
197.00	2.27	0.03	0.44	0.30	5.00	3.00	GBA
201.00	2.24	0.00	0.45	0.53	6.00	3.00	CYP2C19
205.00	1.87	0.01	0.53	0.58	21.00	3.00	SYK
209.00	2.28	0.00	0.44	0.67	4.00	3.00	CYP2D6
211.00	2.04	0.02	0.49	0.53	11.00	3.00	MT-CO1
215.00	1.83	0.00	0.55	0.69	22.00	3.00	JAK2
218.00	2.19	0.01	0.46	0.50	5.00	3.00	F10
220.00	1.90	0.00	0.53	0.75	17.00	3.00	MET
237.00	3.26	0.00	0.31	0.00	1.00	4.00	ASAH1
245.00	2.37	0.00	0.42	0.71	8.00	4.00	FANCI
249.00	2.21	0.00	0.45	0.64	13.00	4.00	AURKB
263.00	2.50	0.00	0.40	0.58	11.00	4.00	C10orf2
264.00	2.28	0.01	0.44	0.42	12.00	4.00	RRM2B
266.00	2.60	0.00	0.38	0.69	10.00	4.00	DGUOK
268.00	2.54	0.01	0.39	0.56	11.00	4.00	POLG
270.00	2.28	0.01	0.44	0.47	10.00	3.00	SLC25A4
272.00	2.62	0.00	0.38	0.86	7.00	4.00	MT-ND6
275.00	2.06	0.01	0.48	0.50	12.00	4.00	MT-CO2
279.00	2.15	0.01	0.46	0.57	8.00	4.00	TYMP
281.00	2.40	0.00	0.42	0.71	7.00	4.00	MPV17
349.00	2.38	0.00	0.42	1.00	5.00	4.00	IDO1
352.00	2.41	0.00	0.41	0.80	6.00	4.00	PPBP
371.00	2.09	0.00	0.48	0.54	14.00	4.00	ZAP70
420.00	2.60	0.00	0.38	0.00	1.00	4.00	CNR2
524.00	2.87	0.00	0.35	0.00	2.00	4.00	F5
571.00	2.85	0.00	0.35	0.00	1.00	4.00	PABPC1
593.00	2.40	0.00	0.42	1.00	4.00	4.00	RORC

NeighborhoodC	NumberOfDi	Radiality	Stress	TopologicalCoefficient
41.50	4.00	0.72	0.00	0.64
22.61	28.00	0.83	2322.00	0.30
19.04	49.00	0.91	6124.00	0.24
20.51	47.00	0.90	3586.00	0.27
20.12	42.00	0.87	3424.00	0.27
26.20	25.00	0.82	1014.00	0.35
23.18	11.00	0.72	148.00	0.39
23.45	29.00	0.82	1282.00	0.33
30.79	14.00	0.77	102.00	0.45
23.90	29.00	0.82	1672.00	0.33
28.09	22.00	0.80	386.00	0.40
28.28	18.00	0.78	370.00	0.42
28.69	13.00	0.73	102.00	0.48
29.14	21.00	0.79	290.00	0.43
26.39	18.00	0.78	306.00	0.38
27.61	23.00	0.81	898.00	0.38
22.66	29.00	0.79	692.00	0.35
22.95	37.00	0.85	1904.00	0.32
32.75	8.00	0.73	68.00	0.51
24.21	19.00	0.79	674.00	0.33
23.12	34.00	0.85	2192.00	0.32
21.81	43.00	0.88	3284.00	0.29
27.42	24.00	0.80	444.00	0.39
22.86	22.00	0.77	298.00	0.37
26.50	12.00	0.77	526.00	0.37
24.80	15.00	0.77	356.00	0.36
31.50	10.00	0.73	38.00	0.51
28.75	8.00	0.71	12.00	0.50
27.31	16.00	0.77	1012.00	0.41
26.81	16.00	0.78	390.00	0.38
27.00	10.00	0.75	290.00	0.40
24.47	19.00	0.75	184.00	0.41
19.36	11.00	0.76	1192.00	0.28
22.95	41.00	0.86	1954.00	0.32
20.00	13.00	0.76	966.00	0.29
25.52	23.00	0.80	938.00	0.35
29.31	13.00	0.76	74.00	0.43
25.00	9.00	0.73	154.00	0.39
23.63	19.00	0.74	142.00	0.41
23.74	19.00	0.79	1482.00	0.33
23.36	14.00	0.79	1370.00	0.31
24.25	16.00	0.78	604.00	0.34
30.78	18.00	0.78	122.00	0.45
23.30	20.00	0.79	822.00	0.33
29.19	16.00	0.77	98.00	0.43
21.97	34.00	0.84	2214.00	0.30
27.42	19.00	0.78	224.00	0.40
26.36	11.00	0.74	210.00	0.41
22.25	8.00	0.72	126.00	0.36
17.40	5.00	0.69	22.00	0.33

21.67	3.00	0.67	8.00	0.42
29.29	17.00	0.78	230.00	0.42
13.80	5.00	0.68	584.00	0.27
15.00	6.00	0.69	40.00	0.28
26.14	21.00	0.78	424.00	0.39
17.50	4.00	0.68	16.00	0.34
16.55	11.00	0.74	1042.00	0.26
27.36	22.00	0.79	350.00	0.40
22.20	5.00	0.70	228.00	0.39
28.29	17.00	0.78	206.00	0.41
5.00	1.00	0.44	0.00	0.00
16.00	8.00	0.66	140.00	0.38
19.62	13.00	0.70	144.00	0.38
10.73	11.00	0.63	150.00	0.33
14.00	12.00	0.68	552.00	0.30
10.00	10.00	0.60	58.00	0.38
9.73	11.00	0.62	130.00	0.33
12.00	10.00	0.68	402.00	0.26
11.29	7.00	0.60	104.00	0.43
15.08	12.00	0.73	926.00	0.24
17.25	8.00	0.71	676.00	0.29
12.86	7.00	0.65	222.00	0.32
34.20	5.00	0.65	0.00	0.73
25.50	6.00	0.65	6.00	0.58
24.79	14.00	0.73	198.00	0.43
34.00	1.00	0.60	0.00	0.00
10.50	2.00	0.53	2.00	0.59
14.00	1.00	0.54	0.00	0.00
35.50	4.00	0.65	0.00	0.76
