# Supplementary material for: Identifying and Targeting Prediction of the PI3K-AKT Signaling Pathway in Drug-Induced Thrombocytopenia in Infected Patients Receiving Linezolid Therapy: A Network Pharmacology-Based Analysis
Source: J Healthc Eng. 2022 Oct 15;2022:2282351. doi: 10.1155/2022/2282351 (PMC9588367; doi:10.1155/2022/2282351)
Supplement: Supplementary Materials — Supplementary Table 1 and experimental dataset are provided for MCODE cluster analysis. Supplementary data files for all the figures are also provided in the supplementary materials. [file 2282351.f1.zip › Figure 5-network data.pdf]

Linezolid CYP3A4
Linezolid CYP2D6
Linezolid CYP2C9
Linezolid F5
Linezolid POLG
Linezolid TYMP
Linezolid ALB
Linezolid IL6
Linezolid COX2
Linezolid PIK3C2A
Linezolid CYP2C19
Linezolid IFNG
Linezolid CCL2
Linezolid IL10
Linezolid RRM2B
Linezolid SLC25A4
Linezolid DGUOK
Linezolid FANCI
Linezolid MPV17
Linezolid TWNK
Linezolid TRNM
Linezolid PPBP
Linezolid NLRP3
Linezolid TRNL1
Linezolid TRNK
Linezolid TRNF
Linezolid CAT
Linezolid HSPD1
Linezolid EPO
Linezolid COX1
Linezolid ND6
Linezolid COX3
Linezolid CYTB
Linezolid TRNS2
Linezolid F10
Linezolid STAT3
Linezolid GBA
Linezolid MAPK14
Linezolid PRKDC
Linezolid RORC
Linezolid KDR
Linezolid PIK3CB
Linezolid CHEK1

Linezolid P2RX7
Linezolid SYK
Linezolid MET
Linezolid LRRK2
Linezolid RAF1
Linezolid JAK2
Linezolid PARP1
Linezolid ASAH1
Linezolid CNR2
Linezolid PIK3CD
Linezolid PIK3CG
Linezolid PIK3CA
Linezolid ALOX5
Linezolid PLAT
Linezolid KLKB1
Linezolid MAPK1
Linezolid CD38
Linezolid TBK1
Linezolid CASP7
Linezolid AKT1
Linezolid CASP1
Linezolid PABPC1
Linezolid FLT1
Linezolid FGFR1
Linezolid FLT3
Linezolid ACHE
Linezolid ELANE
Linezolid CDK2
Linezolid IDO1
Linezolid ERBB2
Linezolid EGFR
Linezolid MTOR
Linezolid AURKB
Linezolid CDK1
Linezolid CDK4
Linezolid NAAA
Linezolid AURKA
Linezolid ZAP70
Linezolid SELE
Linezolid KIT
Linezolid MAPK8
Linezolid RAD51
Thrombocytopenia CYP3A4
Thrombocytopenia CYP2D6

Thrombocytopenia	CYP2C9
Thrombocytopenia	F5
Thrombocytopenia	POLG
Thrombocytopenia	TYMP
Thrombocytopenia	ALB
Thrombocytopenia	IL6
Thrombocytopenia	COX2
Thrombocytopenia	PIK3C2A
Thrombocytopenia	CYP2C19
Thrombocytopenia	IFNG
Thrombocytopenia	CCL2
Thrombocytopenia	IL10
Thrombocytopenia	RRM2B
Thrombocytopenia	SLC25A4
Thrombocytopenia	DGUOK
Thrombocytopenia	FANCI
Thrombocytopenia	MPV17
Thrombocytopenia	TWNK
Thrombocytopenia	TRNM
Thrombocytopenia	PPBP
Thrombocytopenia	NLRP3
Thrombocytopenia	TRNL1
Thrombocytopenia	TRNK
Thrombocytopenia	TRNF
Thrombocytopenia	CAT
Thrombocytopenia	HSPD1
Thrombocytopenia	EPO
Thrombocytopenia	COX1
Thrombocytopenia	ND6
Thrombocytopenia	COX3
Thrombocytopenia	CYTB
Thrombocytopenia	TRNS2
Thrombocytopenia	F10
Thrombocytopenia	STAT3
Thrombocytopenia	GBA
Thrombocytopenia	MAPK14
Thrombocytopenia	PRKDC
Thrombocytopenia	RORC
Thrombocytopenia	KDR
Thrombocytopenia	PIK3CB
Thrombocytopenia	CHEK1
Thrombocytopenia	P2RX7
Thrombocytopenia	SYK
Thrombocytopenia	MET

Thrombocytopenia	LRRK2
Thrombocytopenia	RAF1
Thrombocytopenia	JAK2
Thrombocytopenia	PARP1
Thrombocytopenia	ASAH1
Thrombocytopenia	CNR2
Thrombocytopenia	PIK3CD
Thrombocytopenia	PIK3CG
Thrombocytopenia	PIK3CA
Thrombocytopenia	ALOX5
Thrombocytopenia	PLAT
Thrombocytopenia	KLKB1
Thrombocytopenia	MAPK1
Thrombocytopenia	CD38
Thrombocytopenia	TBK1
Thrombocytopenia	CASP7
Thrombocytopenia	AKT1
Thrombocytopenia	CASP1
Thrombocytopenia	PABPC1
Thrombocytopenia	FLT1
Thrombocytopenia	FGFR1
Thrombocytopenia	FLT3
Thrombocytopenia	ACHE
Thrombocytopenia	ELANE
Thrombocytopenia	CDK2
Thrombocytopenia	IDO1
Thrombocytopenia	ERBB2
Thrombocytopenia	EGFR
Thrombocytopenia	MTOR
Thrombocytopenia	AURKB
Thrombocytopenia	CDK1
Thrombocytopenia	CDK4
Thrombocytopenia	NAAA
Thrombocytopenia	AURKA
Thrombocytopenia	ZAP70
Thrombocytopenia	SELE
Thrombocytopenia	KIT
Thrombocytopenia	MAPK8
Thrombocytopenia	RAD51
