# Supplementary material for: Identifying and Targeting Prediction of the PI3K-AKT Signaling Pathway in Drug-Induced Thrombocytopenia in Infected Patients Receiving Linezolid Therapy: A Network Pharmacology-Based Analysis
Source: J Healthc Eng. 2022 Oct 15;2022:2282351. doi: 10.1155/2022/2282351 (PMC9588367; doi:10.1155/2022/2282351)
Supplement: Supplementary Materials — Supplementary Table 1 and experimental dataset are provided for MCODE cluster analysis. Supplementary data files for all the figures are also provided in the supplementary materials. [file 2282351.f1.zip › Figure 6-Go enrichment data.pdf]

ONTOLOGY	ID	GeneRatio	BgRatio	pvalue	p.adjust	qvalue	Count
BP	GO:0014065	17/80	154/18866	8.40E-20	2.71E-16	1.34E-16	17
BP	GO:0048015	17/80	192/18866	3.81E-18	5.33E-15	2.64E-15	17
BP	GO:0048017	17/80	196/18866	5.43E-18	5.33E-15	2.64E-15	17
BP	GO:0018209	20/80	333/18866	6.60E-18	5.33E-15	2.64E-15	20
BP	GO:0018105	19/80	310/18866	3.39E-17	2.19E-14	1.08E-14	19
BP	GO:0032496	19/80	334/18866	1.35E-16	7.27E-14	3.60E-14	19
BP	GO:0014066	14/80	127/18866	1.95E-16	9.00E-14	4.46E-14	14
BP	GO:0071902	19/80	345/18866	2.46E-16	9.92E-14	4.91E-14	19
BP	GO:0002237	19/80	356/18866	4.38E-16	1.57E-13	7.78E-14	19
BP	GO:0042110	21/80	483/18866	6.09E-16	1.97E-13	9.73E-14	21
BP	GO:0043406	17/80	264/18866	8.33E-16	2.45E-13	1.21E-13	17
BP	GO:0014068	12/80	88/18866	2.27E-15	6.11E-13	3.02E-13	12
BP	GO:0006979	20/80	458/18866	3.09E-15	7.68E-13	3.80E-13	20
BP	GO:0043405	18/80	342/18866	3.67E-15	8.46E-13	4.19E-13	18
BP	GO:0050727	19/80	425/18866	1.11E-14	2.38E-12	1.18E-12	19
BP	GO:0046777	15/80	237/18866	6.32E-14	1.22E-11	6.05E-12	15
BP	GO:0072593	16/80	288/18866	6.43E-14	1.22E-11	6.05E-12	16
BP	GO:0060326	16/80	311/18866	2.11E-13	3.78E-11	1.87E-11	16
BP	GO:0022407	18/80	439/18866	2.67E-13	4.54E-11	2.25E-11	18
BP	GO:1903037	16/80	329/18866	5.00E-13	8.07E-11	4.00E-11	16
BP	GO:0000302	14/80	235/18866	1.07E-12	1.64E-10	8.14E-11	14
BP	GO:0033002	14/80	244/18866	1.78E-12	2.62E-10	1.30E-10	14
BP	GO:0062197	16/80	360/18866	1.97E-12	2.77E-10	1.37E-10	16
BP	GO:0045785	17/80	428/18866	2.23E-12	3.00E-10	1.49E-10	17
BP	GO:0007159	16/80	364/18866	2.33E-12	3.01E-10	1.49E-10	16
BP	GO:2000377	13/80	200/18866	2.44E-12	3.03E-10	1.50E-10	13
BP	GO:0034599	15/80	310/18866	3.11E-12	3.72E-10	1.84E-10	15
BP	GO:0018108	16/80	374/18866	3.52E-12	3.98E-10	1.97E-10	16
BP	GO:0070661	15/80	313/18866	3.57E-12	3.98E-10	1.97E-10	15
BP	GO:0018212	16/80	377/18866	3.97E-12	4.27E-10	2.11E-10	16
BP	GO:0007568	15/80	319/18866	4.69E-12	4.88E-10	2.42E-10	15
BP	GO:0032147	15/80	331/18866	7.94E-12	8.01E-10	3.96E-10	15
BP	GO:0043491	14/80	278/18866	1.04E-11	1.02E-09	5.03E-10	14
BP	GO:0022409	14/80	279/18866	1.09E-11	1.03E-09	5.12E-10	14
BP	GO:0030595	13/80	232/18866	1.60E-11	1.47E-09	7.30E-10	13
BP	GO:1903039	13/80	235/18866	1.88E-11	1.68E-09	8.34E-10	13
BP	GO:1901214	14/80	321/18866	7.06E-11	6.16E-09	3.05E-09	14
BP	GO:0042493	15/80	397/18866	1.03E-10	8.77E-09	4.34E-09	15
BP	GO:0034614	11/80	170/18866	1.41E-10	1.16E-08	5.76E-09	11
BP	GO:0046651	13/80	283/18866	1.90E-10	1.53E-08	7.59E-09	13
BP	GO:0010506	14/80	347/18866	1.97E-10	1.55E-08	7.67E-09	14
BP	GO:0032943	13/80	286/18866	2.16E-10	1.66E-08	8.24E-09	13
BP	GO:0051897	11/80	178/18866	2.30E-10	1.71E-08	8.49E-09	11
BP	GO:0050867	15/80	421/18866	2.34E-10	1.71E-08	8.49E-09	15
BP	GO:0051222	14/80	354/18866	2.56E-10	1.83E-08	9.08E-09	14
BP	GO:0046686	8/80	64/18866	2.67E-10	1.87E-08	9.27E-09	8
BP	GO:0070997	14/80	360/18866	3.19E-10	2.19E-08	1.08E-08	14
BP	GO:0030098	14/80	368/18866	4.25E-10	2.85E-08	1.41E-08	14
BP	GO:1904951	14/80	370/18866	4.56E-10	3.00E-08	1.49E-08	14
BP	GO:0071216	12/80	246/18866	5.08E-10	3.28E-08	1.62E-08	12
BP	GO:0001819	15/80	447/18866	5.35E-10	3.39E-08	1.68E-08	15
BP	GO:0050673	15/80	453/18866	6.43E-10	3.99E-08	1.97E-08	15
BP	GO:0030217	12/80	253/18866	7.01E-10	4.19E-08	2.07E-08	12
BP	GO:0051896	12/80	253/18866	7.01E-10	4.19E-08	2.07E-08	12
BP	GO:1903426	9/80	107/18866	7.16E-10	4.20E-08	2.08E-08	9
BP	GO:0031349	14/80	385/18866	7.64E-10	4.40E-08	2.18E-08	14
BP	GO:0048872	12/80	256/18866	8.01E-10	4.54E-08	2.25E-08	12

BP	GO:0031100	8/80	75/18866	9.80E-10	5.45E-08	2.70E-08	8
BP	GO:0071222	11/80	208/18866	1.21E-09	6.62E-08	3.28E-08	11
BP	GO:0050729	10/80	158/18866	1.27E-09	6.83E-08	3.38E-08	10
BP	GO:0050863	13/80	332/18866	1.34E-09	7.10E-08	3.51E-08	13
BP	GO:0002696	14/80	406/18866	1.52E-09	7.90E-08	3.91E-08	14
BP	GO:0018107	9/80	117/18866	1.59E-09	8.17E-08	4.04E-08	9
BP	GO:0007596	13/80	343/18866	1.99E-09	1.00E-07	4.97E-08	13
BP	GO:0036092	5/80	14/18866	2.35E-09	1.16E-07	5.73E-08	5
BP	GO:0007599	13/80	348/18866	2.37E-09	1.16E-07	5.73E-08	13
BP	GO:0071219	11/80	222/18866	2.40E-09	1.16E-07	5.73E-08	11
BP	GO:0050817	13/80	349/18866	2.45E-09	1.16E-07	5.75E-08	13
BP	GO:0032731	7/80	53/18866	2.48E-09	1.16E-07	5.75E-08	7
BP	GO:0018210	9/80	126/18866	3.08E-09	1.42E-07	7.03E-08	9
BP	GO:1903409	9/80	128/18866	3.54E-09	1.61E-07	7.97E-08	9
BP	GO:0051052	13/80	365/18866	4.21E-09	1.89E-07	9.34E-08	13
BP	GO:0046427	8/80	90/18866	4.28E-09	1.89E-07	9.36E-08	8
BP	GO:0010038	13/80	366/18866	4.35E-09	1.90E-07	9.39E-08	13
BP	GO:0045862	13/80	370/18866	4.96E-09	2.13E-07	1.06E-07	13
BP	GO:0051770	5/80	16/18866	5.09E-09	2.16E-07	1.07E-07	5
BP	GO:0009314	14/80	447/18866	5.20E-09	2.18E-07	1.08E-07	14
BP	GO:1904894	8/80	93/18866	5.56E-09	2.30E-07	1.14E-07	8
BP	GO:0032732	7/80	60/18866	6.08E-09	2.48E-07	1.23E-07	7
BP	GO:0017038	10/80	200/18866	1.24E-08	4.99E-07	2.47E-07	10
BP	GO:0006606	9/80	150/18866	1.43E-08	5.70E-07	2.82E-07	9
BP	GO:0046632	8/80	106/18866	1.58E-08	6.13E-07	3.03E-07	8
BP	GO:2000379	8/80	106/18866	1.58E-08	6.13E-07	3.03E-07	8
BP	GO:0016572	6/80	40/18866	1.64E-08	6.32E-07	3.13E-07	6
BP	GO:0051767	5/80	20/18866	1.78E-08	6.68E-07	3.31E-07	5
BP	GO:0051769	5/80	20/18866	1.78E-08	6.68E-07	3.31E-07	5
BP	GO:0002262	9/80	154/18866	1.80E-08	6.68E-07	3.31E-07	9
BP	GO:0071887	8/80	108/18866	1.83E-08	6.70E-07	3.32E-07	8
BP	GO:0030258	11/80	271/18866	1.91E-08	6.94E-07	3.44E-07	11
BP	GO:0014823	7/80	71/18866	2.02E-08	7.15E-07	3.54E-07	7
BP	GO:0042531	7/80	71/18866	2.02E-08	7.15E-07	3.54E-07	7
BP	GO:1901215	10/80	212/18866	2.15E-08	7.56E-07	3.74E-07	10
BP	GO:0032652	8/80	111/18866	2.27E-08	7.87E-07	3.90E-07	8
BP	GO:0050870	10/80	214/18866	2.36E-08	8.09E-07	4.00E-07	10
BP	GO:0015980	11/80	278/18866	2.49E-08	8.45E-07	4.18E-07	11
BP	GO:0055093	5/80	22/18866	3.01E-08	1.01E-06	5.00E-07	5
BP	GO:0007259	9/80	164/18866	3.11E-08	1.04E-06	5.12E-07	9
BP	GO:0051251	12/80	357/18866	3.33E-08	1.09E-06	5.38E-07	12
BP	GO:0097529	10/80	222/18866	3.34E-08	1.09E-06	5.38E-07	10
BP	GO:0051100	9/80	169/18866	4.03E-08	1.30E-06	6.45E-07	9
BP	GO:0048732	13/80	443/18866	4.18E-08	1.34E-06	6.61E-07	13
BP	GO:0051170	9/80	171/18866	4.47E-08	1.40E-06	6.93E-07	9
BP	GO:0032612	8/80	121/18866	4.47E-08	1.40E-06	6.93E-07	8
BP	GO:1900182	7/80	80/18866	4.67E-08	1.44E-06	7.15E-07	7
BP	GO:0050714	9/80	172/18866	4.70E-08	1.44E-06	7.15E-07	9
BP	GO:0048660	9/80	173/18866	4.94E-08	1.50E-06	7.44E-07	9
BP	GO:0097696	9/80	174/18866	5.19E-08	1.57E-06	7.75E-07	9
BP	GO:0048659	9/80	175/18866	5.45E-08	1.63E-06	8.07E-07	9
BP	GO:0009306	13/80	462/18866	6.82E-08	2.02E-06	1.00E-06	13
BP	GO:0035592	13/80	463/18866	6.99E-08	2.05E-06	1.02E-06	13
BP	GO:0010507	7/80	85/18866	7.14E-08	2.08E-06	1.03E-06	7
BP	GO:0042509	7/80	86/18866	7.74E-08	2.23E-06	1.10E-06	7
BP	GO:0071692	13/80	470/18866	8.32E-08	2.38E-06	1.18E-06	13
BP	GO:0001936	9/80	184/18866	8.39E-08	2.38E-06	1.18E-06	9
BP	GO:0031331	12/80	390/18866	8.74E-08	2.45E-06	1.21E-06	12

BP	GO:0045333	9/80	187/18866	9.64E-08	2.68E-06	1.33E-06	9
BP	GO:0007260	7/80	89/18866	9.83E-08	2.71E-06	1.34E-06	7
BP	GO:0050678	12/80	395/18866	1.00E-07	2.74E-06	1.35E-06	12
BP	GO:0009416	11/80	319/18866	1.01E-07	2.74E-06	1.35E-06	11
BP	GO:0009895	11/80	323/18866	1.14E-07	3.08E-06	1.52E-06	11
BP	GO:0032655	6/80	55/18866	1.18E-07	3.15E-06	1.56E-06	6
BP	GO:0071496	11/80	326/18866	1.26E-07	3.31E-06	1.64E-06	11
BP	GO:0002793	9/80	193/18866	1.26E-07	3.31E-06	1.64E-06	9
BP	GO:0036296	5/80	29/18866	1.32E-07	3.42E-06	1.69E-06	5
BP	GO:2000108	5/80	29/18866	1.32E-07	3.42E-06	1.69E-06	5
BP	GO:0042098	9/80	195/18866	1.38E-07	3.50E-06	1.73E-06	9
BP	GO:0050731	9/80	195/18866	1.38E-07	3.50E-06	1.73E-06	9
BP	GO:0009411	8/80	140/18866	1.39E-07	3.51E-06	1.74E-06	8
BP	GO:0032615	6/80	57/18866	1.47E-07	3.67E-06	1.82E-06	6
BP	GO:0008202	11/80	332/18866	1.51E-07	3.75E-06	1.85E-06	11
BP	GO:0046425	8/80	142/18866	1.55E-07	3.82E-06	1.89E-06	8
BP	GO:0050730	10/80	263/18866	1.63E-07	3.98E-06	1.97E-06	10
BP	GO:0001935	9/80	199/18866	1.64E-07	3.98E-06	1.97E-06	9
BP	GO:0051054	9/80	200/18866	1.71E-07	4.12E-06	2.04E-06	9
BP	GO:0031330	10/80	265/18866	1.75E-07	4.19E-06	2.07E-06	10
BP	GO:0032651	7/80	97/18866	1.78E-07	4.20E-06	2.08E-06	7
BP	GO:0031099	9/80	201/18866	1.78E-07	4.20E-06	2.08E-06	9
BP	GO:0042542	8/80	146/18866	1.92E-07	4.46E-06	2.21E-06	8
BP	GO:0046631	8/80	146/18866	1.92E-07	4.46E-06	2.21E-06	8
BP	GO:0042116	7/80	101/18866	2.36E-07	5.43E-06	2.69E-06	7
BP	GO:0045927	10/80	274/18866	2.39E-07	5.46E-06	2.70E-06	10
BP	GO:0002294	6/80	62/18866	2.44E-07	5.56E-06	2.75E-06	6
BP	GO:0009612	9/80	209/18866	2.48E-07	5.58E-06	2.76E-06	9
BP	GO:1904892	8/80	151/18866	2.49E-07	5.58E-06	2.76E-06	8
BP	GO:0034504	10/80	277/18866	2.64E-07	5.83E-06	2.89E-06	10
BP	GO:0002287	6/80	63/18866	2.69E-07	5.83E-06	2.89E-06	6
BP	GO:0002293	6/80	63/18866	2.69E-07	5.83E-06	2.89E-06	6
BP	GO:0046622	6/80	63/18866	2.69E-07	5.83E-06	2.89E-06	6
BP	GO:0048661	7/80	103/18866	2.70E-07	5.83E-06	2.89E-06	7
BP	GO:0050708	11/80	352/18866	2.71E-07	5.83E-06	2.89E-06	11
BP	GO:0000187	8/80	156/18866	3.20E-07	6.83E-06	3.38E-06	8
BP	GO:0032091	7/80	106/18866	3.28E-07	6.93E-06	3.43E-06	7
BP	GO:0032611	7/80	106/18866	3.28E-07	6.93E-06	3.43E-06	7
BP	GO:0032735	5/80	35/18866	3.55E-07	7.44E-06	3.68E-06	5
BP	GO:0032675	8/80	159/18866	3.70E-07	7.70E-06	3.81E-06	8
BP	GO:0043434	12/80	447/18866	3.79E-07	7.84E-06	3.88E-06	12
BP	GO:0033138	7/80	109/18866	3.97E-07	8.17E-06	4.04E-06	7
BP	GO:0051098	11/80	367/18866	4.10E-07	8.38E-06	4.15E-06	11
BP	GO:0009896	12/80	454/18866	4.47E-07	9.02E-06	4.46E-06	12
BP	GO:0050804	12/80	454/18866	4.47E-07	9.02E-06	4.46E-06	12
BP	GO:0002526	7/80	111/18866	4.50E-07	9.02E-06	4.46E-06	7
BP	GO:0051090	12/80	455/18866	4.57E-07	9.06E-06	4.48E-06	12
BP	GO:0099177	12/80	455/18866	4.57E-07	9.06E-06	4.48E-06	12
BP	GO:0031960	8/80	164/18866	4.68E-07	9.22E-06	4.56E-06	8
BP	GO:0034250	8/80	167/18866	5.38E-07	1.05E-05	5.21E-06	8
BP	GO:0002292	6/80	71/18866	5.53E-07	1.07E-05	5.32E-06	6
BP	GO:0010001	9/80	230/18866	5.57E-07	1.08E-05	5.33E-06	9
BP	GO:0002791	11/80	381/18866	5.94E-07	1.14E-05	5.65E-06	11
BP	GO:0032635	8/80	170/18866	6.15E-07	1.18E-05	5.82E-06	8
BP	GO:1904019	7/80	117/18866	6.44E-07	1.22E-05	6.05E-06	7
BP	GO:0042063	10/80	307/18866	6.77E-07	1.28E-05	6.33E-06	10
BP	GO:0042307	5/80	40/18866	7.08E-07	1.33E-05	6.57E-06	5
BP	GO:0046034	10/80	311/18866	7.62E-07	1.42E-05	7.03E-06	10

BP	GO:0070663	9/80	240/18866	7.94E-07	1.47E-05	7.29E-06	9
BP	GO:0016241	8/80	176/18866	8.00E-07	1.48E-05	7.31E-06	8
BP	GO:1903532	10/80	313/18866	8.07E-07	1.48E-05	7.33E-06	10
BP	GO:0070482	11/80	396/18866	8.68E-07	1.58E-05	7.83E-06	11
BP	GO:1904591	5/80	42/18866	9.09E-07	1.65E-05	8.16E-06	5
BP	GO:0043367	6/80	78/18866	9.68E-07	1.75E-05	8.64E-06	6
BP	GO:0034612	10/80	320/18866	9.87E-07	1.77E-05	8.76E-06	10
BP	GO:0045124	5/80	43/18866	1.02E-06	1.83E-05	9.05E-06	5
BP	GO:0071621	7/80	127/18866	1.12E-06	1.98E-05	9.79E-06	7
BP	GO:1900180	7/80	127/18866	1.12E-06	1.98E-05	9.79E-06	7
BP	GO:2000045	8/80	185/18866	1.17E-06	2.05E-05	1.01E-05	8
BP	GO:0042113	10/80	328/18866	1.23E-06	2.15E-05	1.07E-05	10
BP	GO:0002285	8/80	187/18866	1.26E-06	2.19E-05	1.09E-05	8
BP	GO:0071214	10/80	331/18866	1.34E-06	2.30E-05	1.14E-05	10
BP	GO:0104004	10/80	331/18866	1.34E-06	2.30E-05	1.14E-05	10
BP	GO:0010508	7/80	131/18866	1.38E-06	2.36E-05	1.17E-05	7
BP	GO:0014855	6/80	83/18866	1.40E-06	2.38E-05	1.18E-05	6
BP	GO:0010863	5/80	46/18866	1.44E-06	2.44E-05	1.21E-05	5
BP	GO:1903829	10/80	338/18866	1.62E-06	2.72E-05	1.35E-05	10
BP	GO:0051047	10/80	340/18866	1.70E-06	2.85E-05	1.41E-05	10
BP	GO:0001776	6/80	86/18866	1.72E-06	2.86E-05	1.41E-05	6
BP	GO:2000106	6/80	86/18866	1.72E-06	2.86E-05	1.41E-05	6
BP	GO:0045930	10/80	341/18866	1.75E-06	2.88E-05	1.43E-05	10
BP	GO:0045727	7/80	136/18866	1.78E-06	2.91E-05	1.44E-05	7
BP	GO:0055023	5/80	48/18866	1.79E-06	2.91E-05	1.44E-05	5
BP	GO:1900274	5/80	48/18866	1.79E-06	2.91E-05	1.44E-05	5
BP	GO:0032755	6/80	88/18866	1.97E-06	3.19E-05	1.58E-05	6
BP	GO:0045058	5/80	49/18866	1.99E-06	3.19E-05	1.58E-05	5
BP	GO:0048545	10/80	346/18866	1.99E-06	3.19E-05	1.58E-05	10
BP	GO:0051900	4/80	22/18866	2.07E-06	3.29E-05	1.63E-05	4
BP	GO:0046850	5/80	50/18866	2.20E-06	3.47E-05	1.72E-05	5
BP	GO:1903580	5/80	50/18866	2.20E-06	3.47E-05	1.72E-05	5
BP	GO:0006260	9/80	273/18866	2.31E-06	3.61E-05	1.79E-05	9
BP	GO:0048638	10/80	353/18866	2.39E-06	3.72E-05	1.84E-05	10
BP	GO:0000002	4/80	23/18866	2.50E-06	3.86E-05	1.91E-05	4
BP	GO:1904996	4/80	23/18866	2.50E-06	3.86E-05	1.91E-05	4
BP	GO:1900407	6/80	92/18866	2.56E-06	3.94E-05	1.95E-05	6
BP	GO:1902806	8/80	206/18866	2.61E-06	3.99E-05	1.97E-05	8
BP	GO:0000186	5/80	52/18866	2.69E-06	4.07E-05	2.01E-05	5
BP	GO:0060421	5/80	52/18866	2.69E-06	4.07E-05	2.01E-05	5
BP	GO:0033135	7/80	145/18866	2.72E-06	4.10E-05	2.03E-05	7
BP	GO:0002532	6/80	93/18866	2.73E-06	4.10E-05	2.03E-05	6
BP	GO:0051384	7/80	147/18866	2.98E-06	4.42E-05	2.19E-05	7
BP	GO:0042738	4/80	24/18866	2.99E-06	4.42E-05	2.19E-05	4
BP	GO:0051882	4/80	24/18866	2.99E-06	4.42E-05	2.19E-05	4
BP	GO:0032868	9/80	283/18866	3.10E-06	4.56E-05	2.26E-05	9
BP	GO:0050679	8/80	211/18866	3.12E-06	4.57E-05	2.26E-05	8
BP	GO:0097530	7/80	150/18866	3.41E-06	4.98E-05	2.47E-05	7
BP	GO:0035710	6/80	97/18866	3.49E-06	5.03E-05	2.49E-05	6
BP	GO:0036473	6/80	97/18866	3.49E-06	5.03E-05	2.49E-05	6
BP	GO:0042775	6/80	97/18866	3.49E-06	5.03E-05	2.49E-05	6
BP	GO:0042773	6/80	98/18866	3.71E-06	5.32E-05	2.63E-05	6
BP	GO:0050670	8/80	219/18866	4.10E-06	5.86E-05	2.90E-05	8
BP	GO:0042737	4/80	26/18866	4.17E-06	5.94E-05	2.94E-05	4
BP	GO:0046854	5/80	57/18866	4.26E-06	6.03E-05	2.98E-05	5
BP	GO:0032944	8/80	221/18866	4.39E-06	6.16E-05	3.05E-05	8
BP	GO:0071241	8/80	221/18866	4.39E-06	6.16E-05	3.05E-05	8
BP	GO:1902882	6/80	101/18866	4.42E-06	6.18E-05	3.06E-05	6

BP	GO:0060249	11/80	469/18866	4.45E-06	6.20E-05	3.07E-05	11
BP	GO:0019722	8/80	222/18866	4.54E-06	6.29E-05	3.11E-05	8
BP	GO:0071356	9/80	297/18866	4.59E-06	6.33E-05	3.13E-05	9
BP	GO:0030168	7/80	158/18866	4.81E-06	6.60E-05	3.27E-05	7
BP	GO:0031667	11/80	473/18866	4.83E-06	6.60E-05	3.27E-05	11
BP	GO:0072539	4/80	27/18866	4.88E-06	6.65E-05	3.29E-05	4
BP	GO:0042306	5/80	59/18866	5.06E-06	6.86E-05	3.39E-05	5
BP	GO:0032653	5/80	60/18866	5.50E-06	7.33E-05	3.63E-05	5
BP	GO:0042093	5/80	60/18866	5.50E-06	7.33E-05	3.63E-05	5
BP	GO:0061900	5/80	60/18866	5.50E-06	7.33E-05	3.63E-05	5
BP	GO:1903428	5/80	60/18866	5.50E-06	7.33E-05	3.63E-05	5
BP	GO:0043467	7/80	162/18866	5.68E-06	7.48E-05	3.70E-05	7
BP	GO:0002360	4/80	28/18866	5.68E-06	7.48E-05	3.70E-05	4
BP	GO:0032800	4/80	28/18866	5.68E-06	7.48E-05	3.70E-05	4
BP	GO:0050920	8/80	229/18866	5.70E-06	7.48E-05	3.70E-05	8
BP	GO:0019233	6/80	106/18866	5.85E-06	7.65E-05	3.79E-05	6
BP	GO:0045576	5/80	61/18866	5.97E-06	7.77E-05	3.84E-05	5
BP	GO:0010518	5/80	62/18866	6.47E-06	8.32E-05	4.12E-05	5
BP	GO:0055025	5/80	62/18866	6.47E-06	8.32E-05	4.12E-05	5
BP	GO:1904589	5/80	62/18866	6.47E-06	8.32E-05	4.12E-05	5
BP	GO:0045787	10/80	396/18866	6.60E-06	8.45E-05	4.18E-05	10
BP	GO:0097305	8/80	234/18866	6.68E-06	8.52E-05	4.22E-05	8
BP	GO:0002286	6/80	109/18866	6.88E-06	8.74E-05	4.33E-05	6
BP	GO:0042129	7/80	167/18866	6.93E-06	8.77E-05	4.34E-05	7
BP	GO:0032613	5/80	63/18866	7.00E-06	8.83E-05	4.37E-05	5
BP	GO:0046879	9/80	314/18866	7.19E-06	9.02E-05	4.46E-05	9
BP	GO:0045931	7/80	168/18866	7.21E-06	9.02E-05	4.46E-05	7
BP	GO:0009141	6/80	110/18866	7.25E-06	9.03E-05	4.47E-05	6
BP	GO:0045453	5/80	64/18866	7.57E-06	9.40E-05	4.65E-05	5
BP	GO:0001938	6/80	113/18866	8.47E-06	0.000105	5.18E-05	6
BP	GO:0045348	3/80	10/18866	8.62E-06	0.000106	5.23E-05	3
BP	GO:0051901	3/80	10/18866	8.62E-06	0.000106	5.23E-05	3
BP	GO:0072538	4/80	31/18866	8.64E-06	0.000106	5.23E-05	4
BP	GO:0046824	5/80	66/18866	8.82E-06	0.000107	5.32E-05	5
BP	GO:0009914	9/80	323/18866	9.02E-06	0.000109	5.42E-05	9
BP	GO:0042593	8/80	245/18866	9.34E-06	0.000113	5.59E-05	8
BP	GO:0045123	5/80	67/18866	9.50E-06	0.000114	5.66E-05	5
BP	GO:0033500	8/80	246/18866	9.63E-06	0.000116	5.72E-05	8
BP	GO:0010634	7/80	176/18866	9.77E-06	0.000116	5.76E-05	7
BP	GO:0022904	6/80	116/18866	9.84E-06	0.000116	5.76E-05	6
BP	GO:0046620	6/80	116/18866	9.84E-06	0.000116	5.76E-05	6
BP	GO:0097421	4/80	32/18866	9.85E-06	0.000116	5.76E-05	4
BP	GO:0045428	5/80	68/18866	1.02E-05	0.00012	5.96E-05	5
BP	GO:0033028	4/80	33/18866	1.12E-05	0.000131	6.49E-05	4
BP	GO:1901796	7/80	180/18866	1.13E-05	0.000132	6.55E-05	7
BP	GO:0002534	5/80	70/18866	1.18E-05	0.000137	6.79E-05	5
BP	GO:0035747	3/80	11/18866	1.18E-05	0.000137	6.79E-05	3
BP	GO:0090316	7/80	182/18866	1.22E-05	0.000141	6.96E-05	7
BP	GO:0030218	6/80	121/18866	1.25E-05	0.000144	7.12E-05	6
BP	GO:0016242	4/80	34/18866	1.26E-05	0.000144	7.12E-05	4
BP	GO:0042759	4/80	34/18866	1.26E-05	0.000144	7.12E-05	4
BP	GO:0050715	4/80	34/18866	1.26E-05	0.000144	7.12E-05	4
BP	GO:0022900	7/80	184/18866	1.31E-05	0.000148	7.34E-05	7
BP	GO:0030072	8/80	257/18866	1.32E-05	0.00015	7.41E-05	8
BP	GO:0046834	5/80	72/18866	1.35E-05	0.000152	7.53E-05	5
BP	GO:0050663	5/80	72/18866	1.35E-05	0.000152	7.53E-05	5
BP	GO:0019216	10/80	431/18866	1.38E-05	0.000154	7.62E-05	10
BP	GO:0030099	10/80	431/18866	1.38E-05	0.000154	7.62E-05	10

BP	GO:0010517	5/80	73/18866	1.45E-05	0.000159	7.88E-05	5
BP	GO:0070227	5/80	73/18866	1.45E-05	0.000159	7.88E-05	5
BP	GO:0072401	5/80	73/18866	1.45E-05	0.000159	7.88E-05	5
BP	GO:0072422	5/80	73/18866	1.45E-05	0.000159	7.88E-05	5
BP	GO:0048639	7/80	187/18866	1.45E-05	0.000159	7.88E-05	7
BP	GO:1903578	6/80	125/18866	1.51E-05	0.000165	8.18E-05	6
BP	GO:0051881	5/80	74/18866	1.55E-05	0.000168	8.33E-05	5
BP	GO:0072395	5/80	74/18866	1.55E-05	0.000168	8.33E-05	5
BP	GO:1904181	3/80	12/18866	1.57E-05	0.00017	8.43E-05	3
BP	GO:2000134	6/80	126/18866	1.58E-05	0.000171	8.44E-05	6
BP	GO:0043368	4/80	36/18866	1.59E-05	0.000171	8.48E-05	4
BP	GO:0033157	8/80	264/18866	1.61E-05	0.000172	8.52E-05	8
BP	GO:0035924	5/80	75/18866	1.65E-05	0.000177	8.75E-05	5
BP	GO:0046883	8/80	267/18866	1.74E-05	0.000185	9.16E-05	8
BP	GO:0072331	8/80	267/18866	1.74E-05	0.000185	9.16E-05	8
BP	GO:0060193	5/80	76/18866	1.76E-05	0.000187	9.24E-05	5
BP	GO:1904994	4/80	37/18866	1.78E-05	0.000188	9.29E-05	4
BP	GO:0034101	6/80	129/18866	1.81E-05	0.00019	9.40E-05	6
BP	GO:0006913	9/80	354/18866	1.87E-05	0.000195	9.67E-05	9
BP	GO:0150076	5/80	77/18866	1.88E-05	0.000195	9.67E-05	5
BP	GO:1903201	5/80	77/18866	1.88E-05	0.000195	9.67E-05	5
BP	GO:0051091	8/80	270/18866	1.89E-05	0.000195	9.67E-05	8
BP	GO:1901988	8/80	270/18866	1.89E-05	0.000195	9.67E-05	8
BP	GO:0032733	4/80	38/18866	1.98E-05	0.000203	0.000101	4
BP	GO:0060045	4/80	38/18866	1.98E-05	0.000203	0.000101	4
BP	GO:0071276	4/80	38/18866	1.98E-05	0.000203	0.000101	4
BP	GO:0051169	9/80	357/18866	2.00E-05	0.000204	0.000101	9
BP	GO:0006264	3/80	13/18866	2.04E-05	0.000207	0.000103	3
BP	GO:1902807	6/80	132/18866	2.06E-05	0.000209	0.000103	6
BP	GO:0001666	9/80	359/18866	2.09E-05	0.000211	0.000104	9
BP	GO:0010948	9/80	359/18866	2.09E-05	0.000211	0.000104	9
BP	GO:0006809	5/80	79/18866	2.13E-05	0.000214	0.000106	5
BP	GO:0071260	5/80	79/18866	2.13E-05	0.000214	0.000106	5
BP	GO:0042770	6/80	133/18866	2.15E-05	0.000215	0.000106	6
BP	GO:0042092	4/80	39/18866	2.20E-05	0.000219	0.000109	4
BP	GO:1901654	7/80	200/18866	2.24E-05	0.000222	0.00011	7
BP	GO:0055021	5/80	80/18866	2.26E-05	0.000224	0.000111	5
BP	GO:0010952	7/80	201/18866	2.31E-05	0.000228	0.000113	7
BP	GO:0010631	9/80	365/18866	2.38E-05	0.000234	0.000116	9
BP	GO:0045740	4/80	40/18866	2.44E-05	0.000239	0.000118	4
BP	GO:0090132	9/80	368/18866	2.54E-05	0.000247	0.000122	9
BP	GO:0030183	6/80	137/18866	2.54E-05	0.000247	0.000122	6
BP	GO:1903708	7/80	204/18866	2.54E-05	0.000247	0.000122	7
BP	GO:0051709	3/80	14/18866	2.58E-05	0.00025	0.000124	3
BP	GO:0038083	4/80	41/18866	2.69E-05	0.00026	0.000129	4
BP	GO:0036293	9/80	371/18866	2.71E-05	0.00026	0.000129	9
BP	GO:0048708	5/80	83/18866	2.71E-05	0.00026	0.000129	5
BP	GO:0046209	5/80	84/18866	2.87E-05	0.000275	0.000136	5
BP	GO:0090130	9/80	374/18866	2.88E-05	0.000275	0.000136	9
BP	GO:0000082	8/80	287/18866	2.92E-05	0.000278	0.000138	8
BP	GO:0001889	6/80	141/18866	2.99E-05	0.000284	0.00014	6
BP	GO:0045844	5/80	85/18866	3.04E-05	0.000287	0.000142	5
BP	GO:0048636	5/80	85/18866	3.04E-05	0.000287	0.000142	5
BP	GO:0043393	7/80	211/18866	3.15E-05	0.000297	0.000147	7
BP	GO:1901863	5/80	86/18866	3.22E-05	0.0003	0.000148	5
BP	GO:0045346	3/80	15/18866	3.22E-05	0.0003	0.000148	3
BP	GO:0045779	3/80	15/18866	3.22E-05	0.0003	0.000148	3
BP	GO:2001028	3/80	15/18866	3.22E-05	0.0003	0.000148	3

BP	GO:0045598	6/80	143/18866	3.24E-05	0.0003	0.000149	6
BP	GO:0002685	7/80	212/18866	3.25E-05	0.000301	0.000149	7
BP	GO:0030073	7/80	213/18866	3.35E-05	0.000308	0.000152	7
BP	GO:0090276	7/80	213/18866	3.35E-05	0.000308	0.000152	7
BP	GO:0050921	6/80	144/18866	3.36E-05	0.000308	0.000152	6
BP	GO:0061008	6/80	144/18866	3.36E-05	0.000308	0.000152	6
BP	GO:0006909	9/80	382/18866	3.40E-05	0.000308	0.000152	9
BP	GO:0009144	5/80	87/18866	3.40E-05	0.000308	0.000152	5
BP	GO:0060420	5/80	87/18866	3.40E-05	0.000308	0.000152	5
BP	GO:2001057	5/80	87/18866	3.40E-05	0.000308	0.000152	5
BP	GO:0038127	6/80	145/18866	3.50E-05	0.000315	0.000156	6
BP	GO:0043281	7/80	215/18866	3.55E-05	0.00032	0.000158	7
BP	GO:0006066	9/80	385/18866	3.61E-05	0.000324	0.00016	9
BP	GO:1901987	10/80	486/18866	3.85E-05	0.000344	0.00017	10
BP	GO:0007623	7/80	218/18866	3.88E-05	0.000346	0.000171	7
BP	GO:0045429	4/80	45/18866	3.91E-05	0.000346	0.000171	4
BP	GO:0001780	3/80	16/18866	3.95E-05	0.000346	0.000171	3
BP	GO:0002295	3/80	16/18866	3.95E-05	0.000346	0.000171	3
BP	GO:0002523	3/80	16/18866	3.95E-05	0.000346	0.000171	3
BP	GO:0002830	3/80	16/18866	3.95E-05	0.000346	0.000171	3
BP	GO:0006206	3/80	16/18866	3.95E-05	0.000346	0.000171	3
BP	GO:0045342	3/80	16/18866	3.95E-05	0.000346	0.000171	3
BP	GO:0000075	7/80	219/18866	4.00E-05	0.000349	0.000173	7
BP	GO:0006119	6/80	149/18866	4.08E-05	0.000355	0.000176	6
BP	GO:0090068	8/80	302/18866	4.19E-05	0.000364	0.00018	8
BP	GO:0034103	5/80	91/18866	4.22E-05	0.000364	0.00018	5
BP	GO:0046849	5/80	91/18866	4.22E-05	0.000364	0.00018	5
BP	GO:0070665	6/80	150/18866	4.23E-05	0.000364	0.00018	6
BP	GO:0017144	4/80	46/18866	4.27E-05	0.000366	0.000181	4
BP	GO:1904407	4/80	46/18866	4.27E-05	0.000366	0.000181	4
BP	GO:0032388	7/80	222/18866	4.36E-05	0.000372	0.000184	7
BP	GO:0000077	6/80	151/18866	4.39E-05	0.000373	0.000185	6
BP	GO:0006261	6/80	151/18866	4.39E-05	0.000373	0.000185	6
BP	GO:0006631	9/80	396/18866	4.50E-05	0.000381	0.000189	9
BP	GO:0043303	4/80	47/18866	4.65E-05	0.000391	0.000194	4
BP	GO:0046638	4/80	47/18866	4.65E-05	0.000391	0.000194	4
BP	GO:0070849	4/80	47/18866	4.65E-05	0.000391	0.000194	4
BP	GO:0032677	5/80	93/18866	4.69E-05	0.000393	0.000195	5
BP	GO:0045834	6/80	153/18866	4.73E-05	0.000395	0.000196	6
BP	GO:0046851	3/80	17/18866	4.78E-05	0.000399	0.000197	3
BP	GO:1904035	5/80	94/18866	4.94E-05	0.000411	0.000203	5
BP	GO:0016236	8/80	310/18866	5.04E-05	0.000417	0.000207	8
BP	GO:0044843	8/80	310/18866	5.04E-05	0.000417	0.000207	8
BP	GO:0002279	4/80	48/18866	5.06E-05	0.000418	0.000207	4
BP	GO:0002448	4/80	49/18866	5.49E-05	0.00045	0.000223	4
BP	GO:0002673	4/80	49/18866	5.49E-05	0.00045	0.000223	4
BP	GO:0003254	4/80	49/18866	5.49E-05	0.00045	0.000223	4
BP	GO:0070230	3/80	18/18866	5.72E-05	0.000468	0.000231	3
BP	GO:0071674	5/80	97/18866	5.74E-05	0.000468	0.000232	5
BP	GO:0001774	4/80	50/18866	5.95E-05	0.000477	0.000236	4
BP	GO:0002269	4/80	50/18866	5.95E-05	0.000477	0.000236	4
BP	GO:0002711	4/80	50/18866	5.95E-05	0.000477	0.000236	4
BP	GO:0045981	4/80	50/18866	5.95E-05	0.000477	0.000236	4
BP	GO:0101023	4/80	50/18866	5.95E-05	0.000477	0.000236	4
BP	GO:1900544	4/80	50/18866	5.95E-05	0.000477	0.000236	4
BP	GO:1905562	4/80	50/18866	5.95E-05	0.000477	0.000236	4
BP	GO:0048010	5/80	98/18866	6.03E-05	0.000482	0.000238	5
BP	GO:0001503	9/80	412/18866	6.10E-05	0.000486	0.000241	9

BP	GO:2001233	9/80	413/18866	6.22E-05	0.000494	0.000245	9
BP	GO:0031570	6/80	161/18866	6.28E-05	0.000498	0.000246	6
BP	GO:1902652	6/80	162/18866	6.50E-05	0.000514	0.000254	6
BP	GO:0070301	5/80	100/18866	6.64E-05	0.000524	0.000259	5
BP	GO:0009267	6/80	163/18866	6.72E-05	0.000529	0.000262	6
BP	GO:0043373	3/80	19/18866	6.77E-05	0.000531	0.000263	3
BP	GO:0070989	3/80	19/18866	6.77E-05	0.000531	0.000263	3
BP	GO:2000116	7/80	239/18866	6.94E-05	0.000541	0.000268	7
BP	GO:0032637	5/80	101/18866	6.96E-05	0.000541	0.000268	5
BP	GO:0060191	5/80	101/18866	6.96E-05	0.000541	0.000268	5
BP	GO:0055024	5/80	102/18866	7.30E-05	0.000566	0.00028	5
BP	GO:0061756	4/80	53/18866	7.50E-05	0.00058	0.000287	4
BP	GO:0030593	5/80	103/18866	7.64E-05	0.00059	0.000292	5
BP	GO:0071375	8/80	330/18866	7.81E-05	0.000602	0.000298	8
BP	GO:0002320	3/80	20/18866	7.95E-05	0.000609	0.000302	3
BP	GO:2001169	3/80	20/18866	7.95E-05	0.000609	0.000302	3
BP	GO:0002367	5/80	104/18866	8.00E-05	0.000611	0.000302	5
BP	GO:0043279	5/80	104/18866	8.00E-05	0.000611	0.000302	5
BP	GO:0032757	4/80	54/18866	8.07E-05	0.000614	0.000304	4
BP	GO:0044070	5/80	105/18866	8.37E-05	0.000635	0.000314	5
BP	GO:0045621	5/80	105/18866	8.37E-05	0.000635	0.000314	5
BP	GO:0032102	9/80	433/18866	8.93E-05	0.000675	0.000334	9
BP	GO:0055017	5/80	107/18866	9.16E-05	0.000691	0.000342	5
BP	GO:0009636	7/80	250/18866	9.20E-05	0.000691	0.000342	7
BP	GO:0002363	3/80	21/18866	9.24E-05	0.000691	0.000342	3
BP	GO:0006123	3/80	21/18866	9.24E-05	0.000691	0.000342	3
BP	GO:0019646	3/80	21/18866	9.24E-05	0.000691	0.000342	3
BP	GO:0010332	4/80	56/18866	9.31E-05	0.000693	0.000343	4
BP	GO:0070228	4/80	56/18866	9.31E-05	0.000693	0.000343	4
BP	GO:1901991	7/80	251/18866	9.43E-05	0.0007	0.000347	7
BP	GO:0031668	7/80	253/18866	9.91E-05	0.000734	0.000363	7
BP	GO:0042743	4/80	57/18866	9.98E-05	0.000734	0.000363	4
BP	GO:0072431	4/80	57/18866	9.98E-05	0.000734	0.000363	4
BP	GO:1902400	4/80	57/18866	9.98E-05	0.000734	0.000363	4
BP	GO:0016042	8/80	343/18866	0.000102	0.00075	0.000371	8
BP	GO:0006925	3/80	22/18866	0.000107	0.00077	0.000381	3
BP	GO:0034104	3/80	22/18866	0.000107	0.00077	0.000381	3
BP	GO:0042359	3/80	22/18866	0.000107	0.00077	0.000381	3
BP	GO:0043369	3/80	22/18866	0.000107	0.00077	0.000381	3
BP	GO:0019369	4/80	58/18866	0.000107	0.00077	0.000381	4
BP	GO:0035306	4/80	58/18866	0.000107	0.00077	0.000381	4
BP	GO:1900408	4/80	58/18866	0.000107	0.00077	0.000381	4
BP	GO:1903202	4/80	58/18866	0.000107	0.00077	0.000381	4
BP	GO:1901342	9/80	444/18866	0.000108	0.000776	0.000384	9
BP	GO:0042742	8/80	348/18866	0.000113	0.000805	0.000399	8
BP	GO:0046822	5/80	112/18866	0.000114	0.000805	0.000399	5
BP	GO:0031663	4/80	59/18866	0.000114	0.000805	0.000399	4
BP	GO:0043030	4/80	59/18866	0.000114	0.000805	0.000399	4
BP	GO:0050707	4/80	59/18866	0.000114	0.000805	0.000399	4
BP	GO:0060043	4/80	59/18866	0.000114	0.000805	0.000399	4
BP	GO:0072413	4/80	59/18866	0.000114	0.000805	0.000399	4
BP	GO:1902402	4/80	59/18866	0.000114	0.000805	0.000399	4
BP	GO:1902403	4/80	59/18866	0.000114	0.000805	0.000399	4
BP	GO:1901990	9/80	448/18866	0.000116	0.000813	0.000402	9
BP	GO:0001676	5/80	113/18866	0.000119	0.000832	0.000412	5
BP	GO:0006417	9/80	450/18866	0.00012	0.000833	0.000413	9
BP	GO:0010950	6/80	181/18866	0.00012	0.000833	0.000413	6
BP	GO:0045619	6/80	181/18866	0.00012	0.000833	0.000413	6

BP	GO:0050796	6/80	181/18866	0.00012	0.000833	0.000413	6
BP	GO:0050994	4/80	60/18866	0.000122	0.000842	0.000417	4
BP	GO:1902883	4/80	60/18866	0.000122	0.000842	0.000417	4
BP	GO:1901032	3/80	23/18866	0.000122	0.000842	0.000417	3
BP	GO:1903206	3/80	23/18866	0.000122	0.000842	0.000417	3
BP	GO:1903798	3/80	23/18866	0.000122	0.000842	0.000417	3
BP	GO:0001959	6/80	183/18866	0.000127	0.000872	0.000432	6
BP	GO:1903034	6/80	183/18866	0.000127	0.000872	0.000432	6
BP	GO:0060419	5/80	115/18866	0.000129	0.000881	0.000436	5
BP	GO:0006644	9/80	455/18866	0.00013	0.000886	0.000439	9
BP	GO:0019932	9/80	456/18866	0.000132	0.000897	0.000444	9
BP	GO:0062012	9/80	456/18866	0.000132	0.000897	0.000444	9
BP	GO:0009165	7/80	266/18866	0.000135	0.000917	0.000454	7
BP	GO:0032386	8/80	358/18866	0.000137	0.000929	0.00046	8
BP	GO:1903429	3/80	24/18866	0.000139	0.000937	0.000464	3
BP	GO:2000637	3/80	24/18866	0.000139	0.000937	0.000464	3
BP	GO:0051341	5/80	117/18866	0.00014	0.000937	0.000464	5
BP	GO:0072676	5/80	117/18866	0.00014	0.000937	0.000464	5
BP	GO:0001818	8/80	360/18866	0.000143	0.000955	0.000473	8
BP	GO:1901293	7/80	269/18866	0.000145	0.000966	0.000478	7
BP	GO:0006661	5/80	118/18866	0.000145	0.000966	0.000478	5
BP	GO:0006805	5/80	118/18866	0.000145	0.000966	0.000478	5
BP	GO:0007569	5/80	118/18866	0.000145	0.000966	0.000478	5
BP	GO:0031571	4/80	63/18866	0.000148	0.000977	0.000483	4
BP	GO:0046635	4/80	63/18866	0.000148	0.000977	0.000483	4
BP	GO:0052547	9/80	466/18866	0.000155	0.001026	0.000508	9
BP	GO:0044783	4/80	64/18866	0.000157	0.001027	0.000509	4
BP	GO:0044819	4/80	64/18866	0.000157	0.001027	0.000509	4
BP	GO:0046637	4/80	64/18866	0.000157	0.001027	0.000509	4
BP	GO:0032647	3/80	25/18866	0.000158	0.001027	0.000509	3
BP	GO:0060148	3/80	25/18866	0.000158	0.001027	0.000509	3
BP	GO:0070920	3/80	25/18866	0.000158	0.001027	0.000509	3
BP	GO:2001026	3/80	25/18866	0.000158	0.001027	0.000509	3
BP	GO:0010821	6/80	191/18866	0.000161	0.001041	0.000515	6
BP	GO:0035265	6/80	191/18866	0.000161	0.001041	0.000515	6
BP	GO:1900542	5/80	121/18866	0.000164	0.001058	0.000524	5
BP	GO:0031640	4/80	65/18866	0.000167	0.001076	0.000533	4
BP	GO:0006690	5/80	122/18866	0.00017	0.001089	0.000539	5
BP	GO:0042752	5/80	122/18866	0.00017	0.001089	0.000539	5
BP	GO:1990266	5/80	122/18866	0.00017	0.001089	0.000539	5
BP	GO:0071248	6/80	193/18866	0.00017	0.001089	0.000539	6
BP	GO:0002460	8/80	370/18866	0.000172	0.001099	0.000544	8
BP	GO:0006140	5/80	123/18866	0.000177	0.001124	0.000557	5
BP	GO:0071466	5/80	123/18866	0.000177	0.001124	0.000557	5
BP	GO:0032768	4/80	66/18866	0.000177	0.001124	0.000557	4
BP	GO:0006721	5/80	124/18866	0.000183	0.001163	0.000576	5
BP	GO:0007565	6/80	196/18866	0.000185	0.001165	0.000577	6
BP	GO:0050728	6/80	196/18866	0.000185	0.001165	0.000577	6
BP	GO:0060759	6/80	196/18866	0.000185	0.001165	0.000577	6
BP	GO:0007040	4/80	67/18866	0.000188	0.001173	0.000581	4
BP	GO:0072678	4/80	67/18866	0.000188	0.001173	0.000581	4
BP	GO:0080171	4/80	67/18866	0.000188	0.001173	0.000581	4
BP	GO:1900015	4/80	67/18866	0.000188	0.001173	0.000581	4
BP	GO:0032479	5/80	125/18866	0.00019	0.001189	0.000589	5
BP	GO:0043112	6/80	198/18866	0.000195	0.001217	0.000602	6
BP	GO:0001667	9/80	481/18866	0.000197	0.001224	0.000606	9
BP	GO:0032607	3/80	27/18866	0.0002	0.001236	0.000612	3
BP	GO:0048143	3/80	27/18866	0.0002	0.001236	0.000612	3

BP	GO:0032606	5/80	127/18866	0.000205	0.001266	0.000627	5
BP	GO:1905477	5/80	127/18866	0.000205	0.001266	0.000627	5
BP	GO:0060038	4/80	69/18866	0.00021	0.001295	0.000641	4
BP	GO:2000191	3/80	28/18866	0.000223	0.00137	0.000678	3
BP	GO:0009410	5/80	130/18866	0.000229	0.001403	0.000694	5
BP	GO:0097193	7/80	290/18866	0.00023	0.001405	0.000696	7
BP	GO:1902105	7/80	290/18866	0.00023	0.001405	0.000696	7
BP	GO:0042594	6/80	206/18866	0.000242	0.001476	0.000731	6
BP	GO:0051604	7/80	293/18866	0.000245	0.001489	0.000737	7
BP	GO:0006282	5/80	132/18866	0.000245	0.001489	0.000737	5
BP	GO:0010595	5/80	132/18866	0.000245	0.001489	0.000737	5
BP	GO:0033032	3/80	29/18866	0.000248	0.001498	0.000741	3
BP	GO:0043032	3/80	29/18866	0.000248	0.001498	0.000741	3
BP	GO:1903706	9/80	498/18866	0.000255	0.001536	0.00076	9
BP	GO:0002709	4/80	73/18866	0.000261	0.001573	0.000779	4
BP	GO:0043280	5/80	134/18866	0.000263	0.001582	0.000783	5
BP	GO:0002573	6/80	210/18866	0.000268	0.001606	0.000795	6
BP	GO:0006109	6/80	210/18866	0.000268	0.001606	0.000795	6
BP	GO:0043457	3/80	30/18866	0.000274	0.001637	0.00081	3
BP	GO:1903205	3/80	30/18866	0.000274	0.001637	0.00081	3
BP	GO:0002703	6/80	211/18866	0.000275	0.001637	0.00081	6
BP	GO:0032481	4/80	74/18866	0.000275	0.001637	0.00081	4
BP	GO:1901653	8/80	398/18866	0.000282	0.001669	0.000826	8
BP	GO:0050671	5/80	136/18866	0.000282	0.001669	0.000826	5
BP	GO:0010632	7/80	301/18866	0.000288	0.001704	0.000843	7
BP	GO:0032418	4/80	75/18866	0.00029	0.001704	0.000844	4
BP	GO:0043627	4/80	75/18866	0.00029	0.001704	0.000844	4
BP	GO:0050805	4/80	75/18866	0.00029	0.001704	0.000844	4
BP	GO:0032946	5/80	137/18866	0.000292	0.001711	0.000847	5
BP	GO:0043523	6/80	214/18866	0.000297	0.001739	0.000861	6
BP	GO:0002705	5/80	138/18866	0.000302	0.001763	0.000873	5
BP	GO:0002675	3/80	31/18866	0.000303	0.001765	0.000874	3
BP	GO:0002828	3/80	31/18866	0.000303	0.001765	0.000874	3
BP	GO:0045765	8/80	403/18866	0.000306	0.001782	0.000882	8
BP	GO:0048511	7/80	305/18866	0.000312	0.001812	0.000897	7
BP	GO:0002440	7/80	306/18866	0.000318	0.001845	0.000913	7
BP	GO:1902749	6/80	217/18866	0.00032	0.00185	0.000916	6
BP	GO:0032355	5/80	141/18866	0.000333	0.001914	0.000948	5
BP	GO:0008210	3/80	32/18866	0.000333	0.001914	0.000948	3
BP	GO:0035767	3/80	32/18866	0.000333	0.001914	0.000948	3
BP	GO:0038128	3/80	32/18866	0.000333	0.001914	0.000948	3
BP	GO:0042310	4/80	78/18866	0.000337	0.001932	0.000956	4
BP	GO:0002687	5/80	142/18866	0.000344	0.001969	0.000975	5
BP	GO:0031669	6/80	221/18866	0.000352	0.002014	0.000997	6
BP	GO:0009205	4/80	79/18866	0.000354	0.002014	0.000997	4
BP	GO:0043536	4/80	79/18866	0.000354	0.002014	0.000997	4
BP	GO:0043552	3/80	33/18866	0.000365	0.002077	0.001028	3
BP	GO:0006720	5/80	144/18866	0.000367	0.002077	0.001028	5
BP	GO:0010212	5/80	144/18866	0.000367	0.002077	0.001028	5
BP	GO:0002699	6/80	223/18866	0.00037	0.002087	0.001033	6
BP	GO:0016485	6/80	223/18866	0.00037	0.002087	0.001033	6
BP	GO:0010833	4/80	81/18866	0.000389	0.002193	0.001085	4
BP	GO:0032869	6/80	226/18866	0.000397	0.00222	0.001099	6
BP	GO:0044706	6/80	226/18866	0.000397	0.00222	0.001099	6
BP	GO:0010922	3/80	34/18866	0.0004	0.00222	0.001099	3
BP	GO:0036474	3/80	34/18866	0.0004	0.00222	0.001099	3
BP	GO:0043276	3/80	34/18866	0.0004	0.00222	0.001099	3
BP	GO:0045648	3/80	34/18866	0.0004	0.00222	0.001099	3

BP	GO:0045907	3/80	34/18866	0.0004	0.00222	0.001099	3
BP	GO:0070102	3/80	34/18866	0.0004	0.00222	0.001099	3
BP	GO:0031644	5/80	148/18866	0.000416	0.002307	0.001142	5
BP	GO:0031145	4/80	83/18866	0.000427	0.002365	0.001171	4
BP	GO:0043524	5/80	149/18866	0.000429	0.002371	0.001174	5
BP	GO:0046474	6/80	230/18866	0.000436	0.002396	0.001186	6
BP	GO:0097191	6/80	230/18866	0.000436	0.002396	0.001186	6
BP	GO:0009112	3/80	35/18866	0.000436	0.002396	0.001186	3
BP	GO:0045580	5/80	150/18866	0.000442	0.002428	0.001202	5
BP	GO:0061041	5/80	151/18866	0.000456	0.002492	0.001234	5
BP	GO:2001056	5/80	151/18866	0.000456	0.002492	0.001234	5
BP	GO:0050851	7/80	325/18866	0.000457	0.002492	0.001234	7
BP	GO:0070371	7/80	325/18866	0.000457	0.002492	0.001234	7
BP	GO:0009266	6/80	233/18866	0.000467	0.002537	0.001256	6
BP	GO:0009199	4/80	85/18866	0.000468	0.002537	0.001256	4
BP	GO:2000779	4/80	85/18866	0.000468	0.002537	0.001256	4
BP	GO:0007050	6/80	234/18866	0.000477	0.002585	0.00128	6
BP	GO:0002822	5/80	153/18866	0.000484	0.00261	0.001292	5
BP	GO:0008203	5/80	153/18866	0.000484	0.00261	0.001292	5
BP	GO:0016202	5/80	153/18866	0.000484	0.00261	0.001292	5
BP	GO:0045444	6/80	235/18866	0.000488	0.002622	0.001298	6
BP	GO:1904018	6/80	235/18866	0.000488	0.002622	0.001298	6
BP	GO:0097756	4/80	86/18866	0.000489	0.002622	0.001298	4
BP	GO:0002706	5/80	154/18866	0.000499	0.002666	0.00132	5
BP	GO:0062013	5/80	154/18866	0.000499	0.002666	0.00132	5
BP	GO:0052548	8/80	434/18866	0.000501	0.002675	0.001324	8
BP	GO:0009060	4/80	87/18866	0.000511	0.002717	0.001345	4
BP	GO:0032436	4/80	87/18866	0.000511	0.002717	0.001345	4
BP	GO:0090218	3/80	37/18866	0.000514	0.00273	0.001351	3
BP	GO:0001890	5/80	156/18866	0.000529	0.0028	0.001386	5
BP	GO:1901861	5/80	156/18866	0.000529	0.0028	0.001386	5
BP	GO:0048634	5/80	157/18866	0.000545	0.002877	0.001424	5
BP	GO:0001892	4/80	89/18866	0.000557	0.002936	0.001453	4
BP	GO:0038093	6/80	241/18866	0.000558	0.002936	0.001453	6
BP	GO:1902107	5/80	159/18866	0.000577	0.003033	0.001501	5
BP	GO:0060416	3/80	39/18866	0.000601	0.003155	0.001562	3
BP	GO:0033273	4/80	91/18866	0.000606	0.003168	0.001568	4
BP	GO:0051899	4/80	91/18866	0.000606	0.003168	0.001568	4
BP	GO:0051402	6/80	245/18866	0.000608	0.003175	0.001572	6
BP	GO:0000723	5/80	161/18866	0.000611	0.003184	0.001576	5
BP	GO:0045582	4/80	92/18866	0.000631	0.003286	0.001626	4
BP	GO:1903427	3/80	40/18866	0.000648	0.003368	0.001667	3
BP	GO:1901992	4/80	94/18866	0.000684	0.003552	0.001758	4
BP	GO:0033574	3/80	41/18866	0.000697	0.003605	0.001784	3
BP	GO:0071548	3/80	41/18866	0.000697	0.003605	0.001784	3
BP	GO:0007093	5/80	166/18866	0.000701	0.00361	0.001787	5
BP	GO:0009408	5/80	166/18866	0.000701	0.00361	0.001787	5
BP	GO:2001242	5/80	166/18866	0.000701	0.00361	0.001787	5
BP	GO:0097194	4/80	95/18866	0.000712	0.003661	0.001812	4
BP	GO:0002819	5/80	168/18866	0.00074	0.003798	0.00188	5
BP	GO:0006509	3/80	42/18866	0.000748	0.003809	0.001886	3
BP	GO:0048246	3/80	42/18866	0.000748	0.003809	0.001886	3
BP	GO:0051602	3/80	42/18866	0.000748	0.003809	0.001886	3
BP	GO:1900371	3/80	42/18866	0.000748	0.003809	0.001886	3
BP	GO:1904037	3/80	42/18866	0.000748	0.003809	0.001886	3
BP	GO:0016125	5/80	169/18866	0.00076	0.003858	0.00191	5
BP	GO:0038095	5/80	169/18866	0.00076	0.003858	0.00191	5
BP	GO:0046634	4/80	97/18866	0.00077	0.003903	0.001932	4

BP	GO:0002676	2/80	10/18866	0.000782	0.003936	0.001949	2
BP	GO:0014041	2/80	10/18866	0.000782	0.003936	0.001949	2
BP	GO:0045792	2/80	10/18866	0.000782	0.003936	0.001949	2
BP	GO:1903431	2/80	10/18866	0.000782	0.003936	0.001949	2
BP	GO:2001243	4/80	98/18866	0.0008	0.003988	0.001974	4
BP	GO:0007584	5/80	171/18866	0.000801	0.003988	0.001974	5
BP	GO:0030307	5/80	171/18866	0.000801	0.003988	0.001974	5
BP	GO:0008631	3/80	43/18866	0.000802	0.003988	0.001974	3
BP	GO:0030808	3/80	43/18866	0.000802	0.003988	0.001974	3
BP	GO:0046688	3/80	43/18866	0.000802	0.003988	0.001974	3
BP	GO:0071364	3/80	43/18866	0.000802	0.003988	0.001974	3
BP	GO:1901031	3/80	43/18866	0.000802	0.003988	0.001974	3
BP	GO:0016570	8/80	468/18866	0.000821	0.004075	0.002017	8
BP	GO:0002244	5/80	172/18866	0.000823	0.00408	0.00202	5
BP	GO:0050830	4/80	99/18866	0.000831	0.004116	0.002038	4
BP	GO:0002697	8/80	470/18866	0.000843	0.00417	0.002064	8
BP	GO:0001894	6/80	261/18866	0.000846	0.004173	0.002066	6
BP	GO:0035307	3/80	44/18866	0.000858	0.004222	0.00209	3
BP	GO:0060612	3/80	44/18866	0.000858	0.004222	0.00209	3
BP	GO:0050848	4/80	100/18866	0.000863	0.004241	0.0021	4
BP	GO:0032200	5/80	174/18866	0.000867	0.004245	0.002101	5
BP	GO:0051099	5/80	174/18866	0.000867	0.004245	0.002101	5
BP	GO:1901568	5/80	175/18866	0.000889	0.004349	0.002153	5
BP	GO:0042102	4/80	101/18866	0.000896	0.004362	0.002159	4
BP	GO:0044773	4/80	101/18866	0.000896	0.004362	0.002159	4
BP	GO:0045639	4/80	101/18866	0.000896	0.004362	0.002159	4
BP	GO:0006775	3/80	45/18866	0.000916	0.004435	0.002195	3
BP	GO:0014002	3/80	45/18866	0.000916	0.004435	0.002195	3
BP	GO:0034198	3/80	45/18866	0.000916	0.004435	0.002195	3
BP	GO:0090311	3/80	45/18866	0.000916	0.004435	0.002195	3
BP	GO:2000060	4/80	102/18866	0.00093	0.004492	0.002224	4
BP	GO:0002328	2/80	11/18866	0.000953	0.004556	0.002255	2
BP	GO:0051712	2/80	11/18866	0.000953	0.004556	0.002255	2
BP	GO:0070106	2/80	11/18866	0.000953	0.004556	0.002255	2
BP	GO:0070673	2/80	11/18866	0.000953	0.004556	0.002255	2
BP	GO:0070757	2/80	11/18866	0.000953	0.004556	0.002255	2
BP	GO:1903800	2/80	11/18866	0.000953	0.004556	0.002255	2
BP	GO:2000551	2/80	11/18866	0.000953	0.004556	0.002255	2
BP	GO:0048771	5/80	178/18866	0.000959	0.004582	0.002268	5
BP	GO:0031641	3/80	46/18866	0.000977	0.004641	0.002297	3
BP	GO:0048538	3/80	46/18866	0.000977	0.004641	0.002297	3
BP	GO:0002429	8/80	481/18866	0.000979	0.004641	0.002297	8
BP	GO:0002757	8/80	481/18866	0.000979	0.004641	0.002297	8
BP	GO:0016569	8/80	481/18866	0.000979	0.004641	0.002297	8
BP	GO:0048469	5/80	179/18866	0.000984	0.004657	0.002305	5
BP	GO:0031348	6/80	269/18866	0.000988	0.004671	0.002312	6
BP	GO:0002456	4/80	104/18866	0.000999	0.004717	0.002335	4
BP	GO:0045017	6/80	270/18866	0.001007	0.004748	0.00235	6
BP	GO:0042326	8/80	484/18866	0.001019	0.004795	0.002374	8
BP	GO:0008654	6/80	271/18866	0.001027	0.004825	0.002388	6
BP	GO:0002824	4/80	105/18866	0.001036	0.004855	0.002403	4
BP	GO:0006953	3/80	47/18866	0.001041	0.004855	0.002403	3
BP	GO:0010569	3/80	47/18866	0.001041	0.004855	0.002403	3
BP	GO:0014075	3/80	47/18866	0.001041	0.004855	0.002403	3
BP	GO:0035722	3/80	47/18866	0.001041	0.004855	0.002403	3
BP	GO:0043312	8/80	487/18866	0.00106	0.004938	0.002444	8
BP	GO:0044839	6/80	273/18866	0.001066	0.004961	0.002456	6
BP	GO:0002708	4/80	106/18866	0.001073	0.004976	0.002463	4

BP	GO:0071156	4/80	106/18866	0.001073	0.004976	0.002463	4
BP	GO:0002283	8/80	490/18866	0.001103	0.005107	0.002528	8
BP	GO:0042220	3/80	48/18866	0.001107	0.005111	0.00253	3
BP	GO:1990928	3/80	48/18866	0.001107	0.005111	0.00253	3
BP	GO:0000018	4/80	107/18866	0.001111	0.005123	0.002536	4
BP	GO:0046488	5/80	185/18866	0.00114	0.005191	0.00257	5
BP	GO:0002551	2/80	12/18866	0.00114	0.005191	0.00257	2
BP	GO:0032494	2/80	12/18866	0.00114	0.005191	0.00257	2
BP	GO:0032621	2/80	12/18866	0.00114	0.005191	0.00257	2
BP	GO:0035404	2/80	12/18866	0.00114	0.005191	0.00257	2
BP	GO:0042368	2/80	12/18866	0.00114	0.005191	0.00257	2
BP	GO:0072540	2/80	12/18866	0.00114	0.005191	0.00257	2
BP	GO:0150065	2/80	12/18866	0.00114	0.005191	0.00257	2
BP	GO:2000105	2/80	12/18866	0.00114	0.005191	0.00257	2
BP	GO:0008593	4/80	108/18866	0.00115	0.005214	0.002581	4
BP	GO:0044774	4/80	108/18866	0.00115	0.005214	0.002581	4
BP	GO:1902750	4/80	108/18866	0.00115	0.005214	0.002581	4
BP	GO:0045165	6/80	278/18866	0.001171	0.005299	0.002623	6
BP	GO:0035196	3/80	49/18866	0.001175	0.005299	0.002623	3
BP	GO:0060711	3/80	49/18866	0.001175	0.005299	0.002623	3
BP	GO:0071349	3/80	49/18866	0.001175	0.005299	0.002623	3
BP	GO:0071478	5/80	188/18866	0.001224	0.005511	0.002728	5
BP	GO:0002821	4/80	110/18866	0.001231	0.005519	0.002732	4
BP	GO:1901800	4/80	110/18866	0.001231	0.005519	0.002732	4
BP	GO:1901989	4/80	110/18866	0.001231	0.005519	0.002732	4
BP	GO:0045646	3/80	50/18866	0.001247	0.005566	0.002755	3
BP	GO:0070671	3/80	50/18866	0.001247	0.005566	0.002755	3
BP	GO:1903727	3/80	50/18866	0.001247	0.005566	0.002755	3
BP	GO:0034440	4/80	111/18866	0.001273	0.005675	0.002809	4
BP	GO:0022408	5/80	191/18866	0.001313	0.005839	0.002891	5
BP	GO:0006275	4/80	112/18866	0.001316	0.005839	0.002891	4
BP	GO:0038084	3/80	51/18866	0.001321	0.005839	0.002891	3
BP	GO:0048146	3/80	51/18866	0.001321	0.005839	0.002891	3
BP	GO:0048255	3/80	51/18866	0.001321	0.005839	0.002891	3
BP	GO:0071354	3/80	51/18866	0.001321	0.005839	0.002891	3
BP	GO:0002679	2/80	13/18866	0.001344	0.005885	0.002913	2
BP	GO:0010623	2/80	13/18866	0.001344	0.005885	0.002913	2
BP	GO:0010870	2/80	13/18866	0.001344	0.005885	0.002913	2
BP	GO:0014842	2/80	13/18866	0.001344	0.005885	0.002913	2
BP	GO:0031053	2/80	13/18866	0.001344	0.005885	0.002913	2
BP	GO:0097531	2/80	13/18866	0.001344	0.005885	0.002913	2
BP	GO:1903236	2/80	13/18866	0.001344	0.005885	0.002913	2
BP	GO:0043542	6/80	286/18866	0.001353	0.005912	0.002926	6
BP	GO:0051403	6/80	286/18866	0.001353	0.005912	0.002926	6
BP	GO:0050999	3/80	52/18866	0.001397	0.006095	0.003017	3
BP	GO:1905475	5/80	195/18866	0.001439	0.006268	0.003103	5
BP	GO:0002040	5/80	196/18866	0.001472	0.006395	0.003166	5
BP	GO:0042176	7/80	397/18866	0.001472	0.006395	0.003166	7
BP	GO:0006754	3/80	54/18866	0.001559	0.006675	0.003304	3
BP	GO:0031050	3/80	54/18866	0.001559	0.006675	0.003304	3
BP	GO:0070918	3/80	54/18866	0.001559	0.006675	0.003304	3
BP	GO:0007100	2/80	14/18866	0.001563	0.006675	0.003304	2
BP	GO:0014841	2/80	14/18866	0.001563	0.006675	0.003304	2
BP	GO:0014854	2/80	14/18866	0.001563	0.006675	0.003304	2
BP	GO:0014857	2/80	14/18866	0.001563	0.006675	0.003304	2
BP	GO:0032042	2/80	14/18866	0.001563	0.006675	0.003304	2
BP	GO:0046512	2/80	14/18866	0.001563	0.006675	0.003304	2
BP	GO:0046520	2/80	14/18866	0.001563	0.006675	0.003304	2

BP	GO:0060397	2/80	14/18866	0.001563	0.006675	0.003304	2
BP	GO:0072672	2/80	14/18866	0.001563	0.006675	0.003304	2
BP	GO:1903799	2/80	14/18866	0.001563	0.006675	0.003304	2
BP	GO:0033559	4/80	118/18866	0.001595	0.006801	0.003367	4
BP	GO:0010389	5/80	200/18866	0.001609	0.00685	0.003391	5
BP	GO:0016233	3/80	55/18866	0.001643	0.006974	0.003452	3
BP	GO:0070741	3/80	55/18866	0.001643	0.006974	0.003452	3
BP	GO:0001763	5/80	201/18866	0.001644	0.006974	0.003452	5
BP	GO:0006310	6/80	299/18866	0.001696	0.007184	0.003556	6
BP	GO:0031098	6/80	300/18866	0.001725	0.007297	0.003612	6
BP	GO:0006977	3/80	56/18866	0.001731	0.007305	0.003616	3
BP	GO:0016447	3/80	56/18866	0.001731	0.007305	0.003616	3
BP	GO:0002831	7/80	409/18866	0.001744	0.007349	0.003638	7
BP	GO:0120254	4/80	121/18866	0.001749	0.00736	0.003643	4
BP	GO:0002281	2/80	15/18866	0.001799	0.00745	0.003688	2
BP	GO:0014856	2/80	15/18866	0.001799	0.00745	0.003688	2
BP	GO:0020027	2/80	15/18866	0.001799	0.00745	0.003688	2
BP	GO:0035745	2/80	15/18866	0.001799	0.00745	0.003688	2
BP	GO:0042362	2/80	15/18866	0.001799	0.00745	0.003688	2
BP	GO:0048308	2/80	15/18866	0.001799	0.00745	0.003688	2
BP	GO:0048313	2/80	15/18866	0.001799	0.00745	0.003688	2
BP	GO:0051299	2/80	15/18866	0.001799	0.00745	0.003688	2
BP	GO:0060391	2/80	15/18866	0.001799	0.00745	0.003688	2
BP	GO:1903960	2/80	15/18866	0.001799	0.00745	0.003688	2
BP	GO:2000402	2/80	15/18866	0.001799	0.00745	0.003688	2
BP	GO:2001171	2/80	15/18866	0.001799	0.00745	0.003688	2
BP	GO:0021782	4/80	122/18866	0.001802	0.00745	0.003688	4
BP	GO:0045471	4/80	122/18866	0.001802	0.00745	0.003688	4
BP	GO:1905517	3/80	57/18866	0.001822	0.007521	0.003723	3
BP	GO:0050852	5/80	206/18866	0.001831	0.007549	0.003737	5
BP	GO:0007173	4/80	123/18866	0.001857	0.007646	0.003785	4
BP	GO:0034284	5/80	207/18866	0.00187	0.007681	0.003802	5
BP	GO:0046890	5/80	207/18866	0.00187	0.007681	0.003802	5
BP	GO:0070372	6/80	306/18866	0.001906	0.007787	0.003855	6
BP	GO:0045766	5/80	208/18866	0.00191	0.007787	0.003855	5
BP	GO:0002688	4/80	124/18866	0.001913	0.007787	0.003855	4
BP	GO:0031929	4/80	124/18866	0.001913	0.007787	0.003855	4
BP	GO:0051101	4/80	124/18866	0.001913	0.007787	0.003855	4
BP	GO:0043489	3/80	58/18866	0.001915	0.007787	0.003855	3
BP	GO:0043551	3/80	58/18866	0.001915	0.007787	0.003855	3
BP	GO:1902373	3/80	58/18866	0.001915	0.007787	0.003855	3
BP	GO:0045088	6/80	307/18866	0.001937	0.007866	0.003894	6
BP	GO:0007098	4/80	125/18866	0.00197	0.007968	0.003944	4
BP	GO:0008637	4/80	125/18866	0.00197	0.007968	0.003944	4
BP	GO:0035270	4/80	125/18866	0.00197	0.007968	0.003944	4
BP	GO:0034764	5/80	210/18866	0.001992	0.008046	0.003983	5
BP	GO:0046456	3/80	59/18866	0.002012	0.008108	0.004014	3
BP	GO:0048008	3/80	59/18866	0.002012	0.008108	0.004014	3
BP	GO:0070555	5/80	211/18866	0.002033	0.008184	0.004051	5
BP	GO:0006268	2/80	16/18866	0.00205	0.008201	0.00406	2
BP	GO:0009263	2/80	16/18866	0.00205	0.008201	0.00406	2
BP	GO:1900451	2/80	16/18866	0.00205	0.008201	0.00406	2
BP	GO:1902969	2/80	16/18866	0.00205	0.008201	0.00406	2
BP	GO:1905050	2/80	16/18866	0.00205	0.008201	0.00406	2
BP	GO:0030278	5/80	212/18866	0.002076	0.008292	0.004105	5
BP	GO:1903052	4/80	127/18866	0.002087	0.008328	0.004122	4
BP	GO:0019229	3/80	60/18866	0.002111	0.008404	0.00416	3
BP	GO:0043388	3/80	60/18866	0.002111	0.008404	0.00416	3

BP	GO:0043161	7/80	424/18866	0.002137	0.008497	0.004206	7
BP	GO:0006006	5/80	214/18866	0.002162	0.008584	0.004249	5
BP	GO:0002576	4/80	129/18866	0.002209	0.008726	0.004319	4
BP	GO:1901657	4/80	129/18866	0.002209	0.008726	0.004319	4
BP	GO:0031343	3/80	61/18866	0.002214	0.008726	0.004319	3
BP	GO:0033619	3/80	61/18866	0.002214	0.008726	0.004319	3
BP	GO:1903078	3/80	61/18866	0.002214	0.008726	0.004319	3
BP	GO:1904356	3/80	61/18866	0.002214	0.008726	0.004319	3
BP	GO:0050665	2/80	17/18866	0.002317	0.009097	0.004503	2
BP	GO:0051969	2/80	17/18866	0.002317	0.009097	0.004503	2
BP	GO:1904355	2/80	17/18866	0.002317	0.009097	0.004503	2
BP	GO:0070527	3/80	62/18866	0.002319	0.009097	0.004503	3
BP	GO:0019218	4/80	131/18866	0.002336	0.00913	0.00452	4
BP	GO:0032434	4/80	131/18866	0.002336	0.00913	0.00452	4
BP	GO:0046887	4/80	131/18866	0.002336	0.00913	0.00452	4
BP	GO:0051656	7/80	432/18866	0.002374	0.009265	0.004586	7
BP	GO:0046486	7/80	434/18866	0.002436	0.009496	0.0047	7
BP	GO:0071333	4/80	134/18866	0.002536	0.009853	0.004878	4
BP	GO:0042130	3/80	64/18866	0.00254	0.009853	0.004878	3
BP	GO:0046173	3/80	64/18866	0.00254	0.009853	0.004878	3
BP	GO:0060135	3/80	64/18866	0.00254	0.009853	0.004878	3
BP	GO:0009200	2/80	18/18866	0.0026	0.010026	0.004963	2
BP	GO:0033194	2/80	18/18866	0.0026	0.010026	0.004963	2
BP	GO:0034501	2/80	18/18866	0.0026	0.010026	0.004963	2
BP	GO:0035994	2/80	18/18866	0.0026	0.010026	0.004963	2
BP	GO:0150078	2/80	18/18866	0.0026	0.010026	0.004963	2
BP	GO:0050853	4/80	135/18866	0.002605	0.010035	0.004968	4
BP	GO:0006687	3/80	65/18866	0.002654	0.010189	0.005044	3
BP	GO:0009206	3/80	65/18866	0.002654	0.010189	0.005044	3
BP	GO:0048247	3/80	65/18866	0.002654	0.010189	0.005044	3
BP	GO:0031023	4/80	136/18866	0.002676	0.010245	0.005072	4
BP	GO:0071331	4/80	136/18866	0.002676	0.010245	0.005072	4
BP	GO:0045732	5/80	225/18866	0.002686	0.010272	0.005085	5
BP	GO:0006163	7/80	442/18866	0.002697	0.010302	0.005099	7
BP	GO:0042391	7/80	443/18866	0.002731	0.01042	0.005158	7
BP	GO:2001020	5/80	226/18866	0.002737	0.010433	0.005164	5
BP	GO:0042552	4/80	137/18866	0.002747	0.010433	0.005165	4
BP	GO:0071326	4/80	137/18866	0.002747	0.010433	0.005165	4
BP	GO:1903038	4/80	137/18866	0.002747	0.010433	0.005165	4
BP	GO:0001933	7/80	444/18866	0.002765	0.010455	0.005175	7
BP	GO:0002562	3/80	66/18866	0.002772	0.010455	0.005175	3
BP	GO:0009145	3/80	66/18866	0.002772	0.010455	0.005175	3
BP	GO:0016444	3/80	66/18866	0.002772	0.010455	0.005175	3
BP	GO:0016445	3/80	66/18866	0.002772	0.010455	0.005175	3
BP	GO:2000378	3/80	66/18866	0.002772	0.010455	0.005175	3
BP	GO:0002548	3/80	67/18866	0.002894	0.010727	0.00531	3
BP	GO:0032890	3/80	67/18866	0.002894	0.010727	0.00531	3
BP	GO:0043550	3/80	67/18866	0.002894	0.010727	0.00531	3
BP	GO:0046513	3/80	67/18866	0.002894	0.010727	0.00531	3
BP	GO:1903672	3/80	67/18866	0.002894	0.010727	0.00531	3
BP	GO:0002433	4/80	139/18866	0.002895	0.010727	0.00531	4
BP	GO:0007272	4/80	139/18866	0.002895	0.010727	0.00531	4
BP	GO:0008366	4/80	139/18866	0.002895	0.010727	0.00531	4
BP	GO:0038096	4/80	139/18866	0.002895	0.010727	0.00531	4
BP	GO:0006670	2/80	19/18866	0.002898	0.010727	0.00531	2
BP	GO:0010759	2/80	19/18866	0.002898	0.010727	0.00531	2
BP	GO:0032930	2/80	19/18866	0.002898	0.010727	0.00531	2
BP	GO:0033189	2/80	19/18866	0.002898	0.010727	0.00531	2

BP	GO:0045780	2/80	19/18866	0.002898	0.010727	0.00531	2
BP	GO:0046852	2/80	19/18866	0.002898	0.010727	0.00531	2
BP	GO:0060252	2/80	19/18866	0.002898	0.010727	0.00531	2
BP	GO:0043254	7/80	449/18866	0.002942	0.010879	0.005385	7
BP	GO:0060968	4/80	140/18866	0.00297	0.010958	0.005424	4
BP	GO:0072655	4/80	140/18866	0.00297	0.010958	0.005424	4
BP	GO:0048738	5/80	231/18866	0.003007	0.011033	0.005461	5
BP	GO:0048568	7/80	451/18866	0.003016	0.011033	0.005461	7
BP	GO:0002637	3/80	68/18866	0.003018	0.011033	0.005461	3
BP	GO:0032642	3/80	68/18866	0.003018	0.011033	0.005461	3
BP	GO:0040014	3/80	68/18866	0.003018	0.011033	0.005461	3
BP	GO:0072577	3/80	68/18866	0.003018	0.011033	0.005461	3
BP	GO:1902369	3/80	68/18866	0.003018	0.011033	0.005461	3
BP	GO:1904377	3/80	68/18866	0.003018	0.011033	0.005461	3
BP	GO:0009743	5/80	233/18866	0.00312	0.011375	0.005631	5
BP	GO:2001234	5/80	233/18866	0.00312	0.011375	0.005631	5
BP	GO:0030879	4/80	142/18866	0.003126	0.011375	0.005631	4
BP	GO:0038094	4/80	142/18866	0.003126	0.011375	0.005631	4
BP	GO:0008286	4/80	143/18866	0.003205	0.011542	0.005713	4
BP	GO:0035296	4/80	143/18866	0.003205	0.011542	0.005713	4
BP	GO:0097746	4/80	143/18866	0.003205	0.011542	0.005713	4
BP	GO:0002544	2/80	20/18866	0.003211	0.011542	0.005713	2
BP	GO:0002726	2/80	20/18866	0.003211	0.011542	0.005713	2
BP	GO:0002902	2/80	20/18866	0.003211	0.011542	0.005713	2
BP	GO:0019373	2/80	20/18866	0.003211	0.011542	0.005713	2
BP	GO:0031998	2/80	20/18866	0.003211	0.011542	0.005713	2
BP	GO:0032303	2/80	20/18866	0.003211	0.011542	0.005713	2
BP	GO:0032727	2/80	20/18866	0.003211	0.011542	0.005713	2
BP	GO:0060965	2/80	20/18866	0.003211	0.011542	0.005713	2
BP	GO:0071695	5/80	235/18866	0.003237	0.011621	0.005753	5
BP	GO:0002218	4/80	144/18866	0.003286	0.011748	0.005816	4
BP	GO:0002700	4/80	144/18866	0.003286	0.011748	0.005816	4
BP	GO:0035150	4/80	144/18866	0.003286	0.011748	0.005816	4
BP	GO:0070585	4/80	144/18866	0.003286	0.011748	0.005816	4
BP	GO:0044242	5/80	236/18866	0.003296	0.011769	0.005826	5
BP	GO:0006650	6/80	343/18866	0.00336	0.011983	0.005932	6
BP	GO:0002431	4/80	145/18866	0.003369	0.01199	0.005935	4
BP	GO:0071322	4/80	145/18866	0.003369	0.01199	0.005935	4
BP	GO:0009201	3/80	71/18866	0.003411	0.012099	0.005989	3
BP	GO:0045739	3/80	71/18866	0.003411	0.012099	0.005989	3
BP	GO:0061180	3/80	71/18866	0.003411	0.012099	0.005989	3
BP	GO:0010594	5/80	238/18866	0.003417	0.012107	0.005993	5
BP	GO:0002683	7/80	463/18866	0.003485	0.012284	0.006081	7
BP	GO:0007265	6/80	346/18866	0.003506	0.012284	0.006081	6
BP	GO:0002693	2/80	21/18866	0.003539	0.012284	0.006081	2
BP	GO:0009110	2/80	21/18866	0.003539	0.012284	0.006081	2
BP	GO:0010310	2/80	21/18866	0.003539	0.012284	0.006081	2
BP	GO:0016540	2/80	21/18866	0.003539	0.012284	0.006081	2
BP	GO:0019370	2/80	21/18866	0.003539	0.012284	0.006081	2
BP	GO:0030878	2/80	21/18866	0.003539	0.012284	0.006081	2
BP	GO:0032516	2/80	21/18866	0.003539	0.012284	0.006081	2
BP	GO:0045943	2/80	21/18866	0.003539	0.012284	0.006081	2
BP	GO:0046514	2/80	21/18866	0.003539	0.012284	0.006081	2
BP	GO:0046519	2/80	21/18866	0.003539	0.012284	0.006081	2
BP	GO:0046641	2/80	21/18866	0.003539	0.012284	0.006081	2
BP	GO:0060716	2/80	21/18866	0.003539	0.012284	0.006081	2
BP	GO:0061042	2/80	21/18866	0.003539	0.012284	0.006081	2
BP	GO:0090312	2/80	21/18866	0.003539	0.012284	0.006081	2

BP	GO:1900543	2/80	21/18866	0.003539	0.012284	0.006081	2
BP	GO:1903978	2/80	21/18866	0.003539	0.012284	0.006081	2
BP	GO:2000269	2/80	21/18866	0.003539	0.012284	0.006081	2
BP	GO:0070988	3/80	72/18866	0.003548	0.012303	0.00609	3
BP	GO:0010675	4/80	148/18866	0.003625	0.012541	0.006208	4
BP	GO:1903670	4/80	148/18866	0.003625	0.012541	0.006208	4
BP	GO:0003012	7/80	467/18866	0.003653	0.012625	0.00625	7
BP	GO:0051966	3/80	73/18866	0.003689	0.012736	0.006305	3
BP	GO:1903364	4/80	149/18866	0.003713	0.012805	0.006339	4
BP	GO:0035264	4/80	150/18866	0.003803	0.0131	0.006485	4
BP	GO:0072521	7/80	472/18866	0.003872	0.013304	0.006585	7
BP	GO:0032928	2/80	22/18866	0.003882	0.013304	0.006585	2
BP	GO:0034312	2/80	22/18866	0.003882	0.013304	0.006585	2
BP	GO:0045980	2/80	22/18866	0.003882	0.013304	0.006585	2
BP	GO:0071459	2/80	22/18866	0.003882	0.013304	0.006585	2
BP	GO:0072330	5/80	246/18866	0.003932	0.01346	0.006663	5
BP	GO:0032602	3/80	75/18866	0.003981	0.013584	0.006724	3
BP	GO:0035019	3/80	75/18866	0.003981	0.013584	0.006724	3
BP	GO:0045685	3/80	75/18866	0.003981	0.013584	0.006724	3
BP	GO:0033044	6/80	356/18866	0.004028	0.013731	0.006797	6
BP	GO:0048285	7/80	476/18866	0.004054	0.013805	0.006834	7
BP	GO:0001678	4/80	153/18866	0.00408	0.013879	0.00687	4
BP	GO:0033077	3/80	76/18866	0.004132	0.014026	0.006943	3
BP	GO:1903524	3/80	76/18866	0.004132	0.014026	0.006943	3
BP	GO:0010288	2/80	23/18866	0.00424	0.014243	0.007051	2
BP	GO:0031281	2/80	23/18866	0.00424	0.014243	0.007051	2
BP	GO:0031639	2/80	23/18866	0.00424	0.014243	0.007051	2
BP	GO:0035162	2/80	23/18866	0.00424	0.014243	0.007051	2
BP	GO:0035743	2/80	23/18866	0.00424	0.014243	0.007051	2
BP	GO:0043586	2/80	23/18866	0.00424	0.014243	0.007051	2
BP	GO:0051043	2/80	23/18866	0.00424	0.014243	0.007051	2
BP	GO:0060149	2/80	23/18866	0.00424	0.014243	0.007051	2
BP	GO:0060967	2/80	23/18866	0.00424	0.014243	0.007051	2
BP	GO:1900017	2/80	23/18866	0.00424	0.014243	0.007051	2
BP	GO:0002833	5/80	251/18866	0.004281	0.014354	0.007105	5
BP	GO:0002200	3/80	77/18866	0.004287	0.014354	0.007105	3
BP	GO:0007422	3/80	77/18866	0.004287	0.014354	0.007105	3
BP	GO:0006936	6/80	362/18866	0.004368	0.014612	0.007233	6
BP	GO:0010498	7/80	483/18866	0.004389	0.014666	0.00726	7
BP	GO:0061045	3/80	78/18866	0.004445	0.014837	0.007344	3
BP	GO:0000086	5/80	254/18866	0.004501	0.014994	0.007422	5
BP	GO:0019318	5/80	254/18866	0.004501	0.014994	0.007422	5
BP	GO:0035051	4/80	158/18866	0.004573	0.015211	0.00753	4
BP	GO:0031016	3/80	79/18866	0.004606	0.015211	0.00753	3
BP	GO:0050672	3/80	79/18866	0.004606	0.015211	0.00753	3
BP	GO:0010869	2/80	24/18866	0.004613	0.015211	0.00753	2
BP	GO:0032753	2/80	24/18866	0.004613	0.015211	0.00753	2
BP	GO:0044346	2/80	24/18866	0.004613	0.015211	0.00753	2
BP	GO:0045821	2/80	24/18866	0.004613	0.015211	0.00753	2
BP	GO:0050995	2/80	24/18866	0.004613	0.015211	0.00753	2
BP	GO:0060396	2/80	24/18866	0.004613	0.015211	0.00753	2
BP	GO:0090335	2/80	24/18866	0.004613	0.015211	0.00753	2
BP	GO:0046394	6/80	367/18866	0.004668	0.015371	0.007609	6
BP	GO:1902600	4/80	159/18866	0.004676	0.015371	0.007609	4
BP	GO:2000058	4/80	159/18866	0.004676	0.015371	0.007609	4
BP	GO:0034765	7/80	489/18866	0.004692	0.015407	0.007627	7
BP	GO:0031334	5/80	257/18866	0.004729	0.015498	0.007672	5
BP	GO:0016053	6/80	368/18866	0.004729	0.015498	0.007672	6

BP	GO:0002312	3/80	80/18866	0.004771	0.015557	0.007701	3
BP	GO:0006110	3/80	80/18866	0.004771	0.015557	0.007701	3
BP	GO:0032204	3/80	80/18866	0.004771	0.015557	0.007701	3
BP	GO:0032945	3/80	80/18866	0.004771	0.015557	0.007701	3
BP	GO:0048678	3/80	80/18866	0.004771	0.015557	0.007701	3
BP	GO:0001937	3/80	81/18866	0.00494	0.016057	0.007949	3
BP	GO:0034644	3/80	81/18866	0.00494	0.016057	0.007949	3
BP	GO:0071158	3/80	81/18866	0.00494	0.016057	0.007949	3
BP	GO:0002092	2/80	25/18866	0.005001	0.016126	0.007982	2
BP	GO:0014829	2/80	25/18866	0.005001	0.016126	0.007982	2
BP	GO:0060259	2/80	25/18866	0.005001	0.016126	0.007982	2
BP	GO:0060330	2/80	25/18866	0.005001	0.016126	0.007982	2
BP	GO:0060334	2/80	25/18866	0.005001	0.016126	0.007982	2
BP	GO:0071378	2/80	25/18866	0.005001	0.016126	0.007982	2
BP	GO:1905564	2/80	25/18866	0.005001	0.016126	0.007982	2
BP	GO:2000679	2/80	25/18866	0.005001	0.016126	0.007982	2
BP	GO:0006665	4/80	163/18866	0.005104	0.016418	0.008127	4
BP	GO:0009142	3/80	82/18866	0.005112	0.016418	0.008127	3
BP	GO:0022617	3/80	82/18866	0.005112	0.016418	0.008127	3
BP	GO:0030193	3/80	82/18866	0.005112	0.016418	0.008127	3
BP	GO:0061448	5/80	262/18866	0.005127	0.01645	0.008143	5
BP	GO:0045637	5/80	263/18866	0.005209	0.016684	0.008259	5
BP	GO:0043535	4/80	164/18866	0.005215	0.016684	0.008259	4
BP	GO:0046165	4/80	164/18866	0.005215	0.016684	0.008259	4
BP	GO:0002718	3/80	83/18866	0.005287	0.016865	0.008348	3
BP	GO:0048145	3/80	83/18866	0.005287	0.016865	0.008348	3
BP	GO:1900046	3/80	83/18866	0.005287	0.016865	0.008348	3
BP	GO:0006959	6/80	377/18866	0.005311	0.016924	0.008377	6
BP	GO:0001783	2/80	26/18866	0.005402	0.017064	0.008447	2
BP	GO:0030810	2/80	26/18866	0.005402	0.017064	0.008447	2
BP	GO:0042730	2/80	26/18866	0.005402	0.017064	0.008447	2
BP	GO:0060390	2/80	26/18866	0.005402	0.017064	0.008447	2
BP	GO:0097066	2/80	26/18866	0.005402	0.017064	0.008447	2
BP	GO:1900373	2/80	26/18866	0.005402	0.017064	0.008447	2
BP	GO:1903649	2/80	26/18866	0.005402	0.017064	0.008447	2
BP	GO:1904353	2/80	26/18866	0.005402	0.017064	0.008447	2
BP	GO:1905523	2/80	26/18866	0.005402	0.017064	0.008447	2
BP	GO:0045913	3/80	84/18866	0.005467	0.017233	0.00853	3
BP	GO:0048144	3/80	84/18866	0.005467	0.017233	0.00853	3
BP	GO:0006970	3/80	85/18866	0.00565	0.017774	0.008799	3
BP	GO:0072527	3/80	85/18866	0.00565	0.017774	0.008799	3
BP	GO:0009651	2/80	27/18866	0.005819	0.018218	0.009018	2
BP	GO:0010971	2/80	27/18866	0.005819	0.018218	0.009018	2
BP	GO:0090140	2/80	27/18866	0.005819	0.018218	0.009018	2
BP	GO:1902175	2/80	27/18866	0.005819	0.018218	0.009018	2
BP	GO:1903203	2/80	27/18866	0.005819	0.018218	0.009018	2
BP	GO:0006919	3/80	86/18866	0.005836	0.018219	0.009019	3
BP	GO:0034109	3/80	86/18866	0.005836	0.018219	0.009019	3
BP	GO:0070664	3/80	86/18866	0.005836	0.018219	0.009019	3
BP	GO:0051781	3/80	87/18866	0.006026	0.018795	0.009304	3
BP	GO:0051302	4/80	171/18866	0.006038	0.018795	0.009304	4
BP	GO:1905952	4/80	171/18866	0.006038	0.018795	0.009304	4
BP	GO:0001906	4/80	172/18866	0.006162	0.019163	0.009486	4
BP	GO:0046889	3/80	88/18866	0.00622	0.019193	0.009501	3
BP	GO:0050818	3/80	88/18866	0.00622	0.019193	0.009501	3
BP	GO:0090559	3/80	88/18866	0.00622	0.019193	0.009501	3
BP	GO:1903312	3/80	88/18866	0.00622	0.019193	0.009501	3
BP	GO:0006972	2/80	28/18866	0.006249	0.019193	0.009501	2

BP	GO:0010818	2/80	28/18866	0.006249	0.019193	0.009501	2
BP	GO:0031645	2/80	28/18866	0.006249	0.019193	0.009501	2
BP	GO:0033561	2/80	28/18866	0.006249	0.019193	0.009501	2
BP	GO:0034311	2/80	28/18866	0.006249	0.019193	0.009501	2
BP	GO:0072376	2/80	28/18866	0.006249	0.019193	0.009501	2
BP	GO:0072378	2/80	28/18866	0.006249	0.019193	0.009501	2
BP	GO:1903579	2/80	28/18866	0.006249	0.019193	0.009501	2
BP	GO:1905563	2/80	28/18866	0.006249	0.019193	0.009501	2
BP	GO:0006633	4/80	173/18866	0.006288	0.019294	0.009551	4
BP	GO:0001659	4/80	174/18866	0.006415	0.019648	0.009726	4
BP	GO:0007033	4/80	174/18866	0.006415	0.019648	0.009726	4
BP	GO:0043470	3/80	90/18866	0.006619	0.020233	0.010015	3
BP	GO:0070542	3/80	90/18866	0.006619	0.020233	0.010015	3
BP	GO:0031063	2/80	29/18866	0.006694	0.020384	0.01009	2
BP	GO:0036336	2/80	29/18866	0.006694	0.020384	0.01009	2
BP	GO:0045822	2/80	29/18866	0.006694	0.020384	0.01009	2
BP	GO:0050901	2/80	29/18866	0.006694	0.020384	0.01009	2
BP	GO:0010959	6/80	396/18866	0.00671	0.020414	0.010105	6
BP	GO:0097306	3/80	91/18866	0.006824	0.020742	0.010267	3
BP	GO:0031341	3/80	92/18866	0.007033	0.021356	0.010571	3
BP	GO:0001822	5/80	283/18866	0.007059	0.021415	0.010601	5
BP	GO:0009150	6/80	401/18866	0.007119	0.021517	0.010651	6
BP	GO:0005979	2/80	30/18866	0.007152	0.021517	0.010651	2
BP	GO:0010758	2/80	30/18866	0.007152	0.021517	0.010651	2
BP	GO:0010962	2/80	30/18866	0.007152	0.021517	0.010651	2
BP	GO:0036475	2/80	30/18866	0.007152	0.021517	0.010651	2
BP	GO:0043372	2/80	30/18866	0.007152	0.021517	0.010651	2
BP	GO:0044030	2/80	30/18866	0.007152	0.021517	0.010651	2
BP	GO:0060055	2/80	30/18866	0.007152	0.021517	0.010651	2
BP	GO:1902751	2/80	30/18866	0.007152	0.021517	0.010651	2
BP	GO:0007411	5/80	284/18866	0.007162	0.021525	0.010655	5
BP	GO:0032088	3/80	93/18866	0.007245	0.021736	0.010759	3
BP	GO:1903035	3/80	93/18866	0.007245	0.021736	0.010759	3
BP	GO:0097485	5/80	285/18866	0.007266	0.021778	0.01078	5
BP	GO:0008361	4/80	181/18866	0.007357	0.022029	0.010905	4
BP	GO:0034308	3/80	94/18866	0.007461	0.022301	0.011039	3
BP	GO:0045778	3/80	94/18866	0.007461	0.022301	0.011039	3
BP	GO:0021700	5/80	287/18866	0.007477	0.022329	0.011053	5
BP	GO:0071346	4/80	182/18866	0.007498	0.02237	0.011073	4
BP	GO:0006356	2/80	31/18866	0.007625	0.022642	0.011208	2
BP	GO:0032770	2/80	31/18866	0.007625	0.022642	0.011208	2
BP	GO:0045737	2/80	31/18866	0.007625	0.022642	0.011208	2
BP	GO:0070229	2/80	31/18866	0.007625	0.022642	0.011208	2
BP	GO:1903792	2/80	31/18866	0.007625	0.022642	0.011208	2
BP	GO:1903725	3/80	95/18866	0.007682	0.02279	0.011282	3
BP	GO:0002695	4/80	184/18866	0.007787	0.023081	0.011425	4
BP	GO:0002690	3/80	96/18866	0.007905	0.023369	0.011568	3
BP	GO:0010972	3/80	96/18866	0.007905	0.023369	0.011568	3
BP	GO:0035249	3/80	96/18866	0.007905	0.023369	0.011568	3
BP	GO:0072001	5/80	292/18866	0.008025	0.0237	0.011732	5
BP	GO:0043488	4/80	186/18866	0.008082	0.023738	0.01175	4
BP	GO:0002724	2/80	32/18866	0.008111	0.023738	0.01175	2
BP	GO:0032673	2/80	32/18866	0.008111	0.023738	0.01175	2
BP	GO:0046320	2/80	32/18866	0.008111	0.023738	0.01175	2
BP	GO:0051968	2/80	32/18866	0.008111	0.023738	0.01175	2
BP	GO:1903959	2/80	32/18866	0.008111	0.023738	0.01175	2
BP	GO:0006476	3/80	97/18866	0.008133	0.023738	0.01175	3
BP	GO:0042100	3/80	97/18866	0.008133	0.023738	0.01175	3

BP	GO:0050764	3/80	97/18866	0.008133	0.023738	0.01175	3
BP	GO:0120162	3/80	97/18866	0.008133	0.023738	0.01175	3
BP	GO:1901184	3/80	97/18866	0.008133	0.023738	0.01175	3
BP	GO:1901216	3/80	97/18866	0.008133	0.023738	0.01175	3
BP	GO:1901655	3/80	97/18866	0.008133	0.023738	0.01175	3
BP	GO:0061138	4/80	187/18866	0.008233	0.024007	0.011884	4
BP	GO:0030316	3/80	98/18866	0.008365	0.024352	0.012055	3
BP	GO:0050768	5/80	295/18866	0.008366	0.024352	0.012055	5
BP	GO:0009259	6/80	416/18866	0.008454	0.024564	0.012159	6
BP	GO:0010639	6/80	416/18866	0.008454	0.024564	0.012159	6
BP	GO:0005996	5/80	296/18866	0.008482	0.024602	0.012178	5
BP	GO:0031647	5/80	296/18866	0.008482	0.024602	0.012178	5
BP	GO:0043534	4/80	189/18866	0.008539	0.024744	0.012249	4
BP	GO:0090277	3/80	99/18866	0.0086	0.024816	0.012284	3
BP	GO:0002691	2/80	33/18866	0.00861	0.024816	0.012284	2
BP	GO:0030149	2/80	33/18866	0.00861	0.024816	0.012284	2
BP	GO:0042755	2/80	33/18866	0.00861	0.024816	0.012284	2
BP	GO:0046640	2/80	33/18866	0.00861	0.024816	0.012284	2
BP	GO:1905048	2/80	33/18866	0.00861	0.024816	0.012284	2
BP	GO:0003018	4/80	190/18866	0.008695	0.025039	0.012395	4
BP	GO:0006672	3/80	100/18866	0.008839	0.025408	0.012577	3
BP	GO:0045807	3/80	100/18866	0.008839	0.025408	0.012577	3
BP	GO:0007162	5/80	301/18866	0.00908	0.02604	0.01289	5
BP	GO:0015908	3/80	101/18866	0.009083	0.02604	0.01289	3
BP	GO:0007202	2/80	34/18866	0.009124	0.02604	0.01289	2
BP	GO:0032148	2/80	34/18866	0.009124	0.02604	0.01289	2
BP	GO:0032212	2/80	34/18866	0.009124	0.02604	0.01289	2
BP	GO:0033198	2/80	34/18866	0.009124	0.02604	0.01289	2
BP	GO:0043094	2/80	34/18866	0.009124	0.02604	0.01289	2
BP	GO:0055094	2/80	34/18866	0.009124	0.02604	0.01289	2
BP	GO:0061136	4/80	193/18866	0.009175	0.026163	0.012951	4
BP	GO:1903522	5/80	303/18866	0.009326	0.026534	0.013135	5
BP	GO:0000079	3/80	102/18866	0.00933	0.026534	0.013135	3
BP	GO:1903076	3/80	102/18866	0.00933	0.026534	0.013135	3
BP	GO:0046578	4/80	194/18866	0.009338	0.026535	0.013135	4
BP	GO:0007219	4/80	195/18866	0.009504	0.026934	0.013333	4
BP	GO:0043487	4/80	195/18866	0.009504	0.026934	0.013333	4
BP	GO:0050864	4/80	195/18866	0.009504	0.026934	0.013333	4
BP	GO:0019693	6/80	427/18866	0.009542	0.026994	0.013362	6
BP	GO:0002042	3/80	103/18866	0.009581	0.026994	0.013362	3
BP	GO:0034766	3/80	103/18866	0.009581	0.026994	0.013362	3
BP	GO:0060079	3/80	103/18866	0.009581	0.026994	0.013362	3
BP	GO:1901570	3/80	103/18866	0.009581	0.026994	0.013362	3
BP	GO:0006471	2/80	35/18866	0.00965	0.026994	0.013362	2
BP	GO:0006691	2/80	35/18866	0.00965	0.026994	0.013362	2
BP	GO:0007435	2/80	35/18866	0.00965	0.026994	0.013362	2
BP	GO:0009262	2/80	35/18866	0.00965	0.026994	0.013362	2
BP	GO:0032633	2/80	35/18866	0.00965	0.026994	0.013362	2
BP	GO:0060251	2/80	35/18866	0.00965	0.026994	0.013362	2
BP	GO:0060674	2/80	35/18866	0.00965	0.026994	0.013362	2
BP	GO:0071312	2/80	35/18866	0.00965	0.026994	0.013362	2
BP	GO:0097009	2/80	35/18866	0.00965	0.026994	0.013362	2
BP	GO:1904031	2/80	35/18866	0.00965	0.026994	0.013362	2
BP	GO:0009116	3/80	104/18866	0.009836	0.027431	0.013578	3
BP	GO:0120034	3/80	104/18866	0.009836	0.027431	0.013578	3
BP	GO:0006164	4/80	197/18866	0.00984	0.027431	0.013578	4
BP	GO:0009749	4/80	197/18866	0.00984	0.027431	0.013578	4
BP	GO:0048259	3/80	105/18866	0.010094	0.028042	0.013881	3

BP	GO:0062014	3/80	105/18866	0.010094	0.028042	0.013881	3
BP	GO:2001022	3/80	105/18866	0.010094	0.028042	0.013881	3
BP	GO:0001893	2/80	36/18866	0.01019	0.028042	0.013881	2
BP	GO:0002701	2/80	36/18866	0.01019	0.028042	0.013881	2
BP	GO:0031572	2/80	36/18866	0.01019	0.028042	0.013881	2
BP	GO:0034405	2/80	36/18866	0.01019	0.028042	0.013881	2
BP	GO:0042554	2/80	36/18866	0.01019	0.028042	0.013881	2
BP	GO:0046633	2/80	36/18866	0.01019	0.028042	0.013881	2
BP	GO:0051973	2/80	36/18866	0.01019	0.028042	0.013881	2
BP	GO:0070873	2/80	36/18866	0.01019	0.028042	0.013881	2
BP	GO:0071402	2/80	36/18866	0.01019	0.028042	0.013881	2
BP	GO:2000310	2/80	36/18866	0.01019	0.028042	0.013881	2
BP	GO:2000516	2/80	36/18866	0.01019	0.028042	0.013881	2
BP	GO:2000781	2/80	36/18866	0.01019	0.028042	0.013881	2
BP	GO:0019395	3/80	106/18866	0.010357	0.028453	0.014085	3
BP	GO:1904029	3/80	106/18866	0.010357	0.028453	0.014085	3
BP	GO:0030148	3/80	107/18866	0.010624	0.029112	0.014411	3
BP	GO:0030330	3/80	107/18866	0.010624	0.029112	0.014411	3
BP	GO:0035601	3/80	107/18866	0.010624	0.029112	0.014411	3
BP	GO:0009746	4/80	202/18866	0.010716	0.02919	0.014449	4
BP	GO:0034341	4/80	202/18866	0.010716	0.02919	0.014449	4
BP	GO:0032717	2/80	37/18866	0.010743	0.02919	0.014449	2
BP	GO:0045730	2/80	37/18866	0.010743	0.02919	0.014449	2
BP	GO:0060969	2/80	37/18866	0.010743	0.02919	0.014449	2
BP	GO:0090050	2/80	37/18866	0.010743	0.02919	0.014449	2
BP	GO:0090322	2/80	37/18866	0.010743	0.02919	0.014449	2
BP	GO:0098926	2/80	37/18866	0.010743	0.02919	0.014449	2
BP	GO:1904358	2/80	37/18866	0.010743	0.02919	0.014449	2
BP	GO:2000273	2/80	37/18866	0.010743	0.02919	0.014449	2
BP	GO:0032508	3/80	108/18866	0.010894	0.029577	0.014641	3
BP	GO:0006939	3/80	109/18866	0.011169	0.030298	0.014998	3
BP	GO:0048608	6/80	443/18866	0.011299	0.030446	0.015071	6
BP	GO:0006862	2/80	38/18866	0.011309	0.030446	0.015071	2
BP	GO:0032094	2/80	38/18866	0.011309	0.030446	0.015071	2
BP	GO:0032885	2/80	38/18866	0.011309	0.030446	0.015071	2
BP	GO:0043029	2/80	38/18866	0.011309	0.030446	0.015071	2
BP	GO:0046466	2/80	38/18866	0.011309	0.030446	0.015071	2
BP	GO:0051930	2/80	38/18866	0.011309	0.030446	0.015071	2
BP	GO:0071392	2/80	38/18866	0.011309	0.030446	0.015071	2
BP	GO:1903523	2/80	38/18866	0.011309	0.030446	0.015071	2
BP	GO:0006664	3/80	110/18866	0.011448	0.030751	0.015222	3
BP	GO:1904659	3/80	110/18866	0.011448	0.030751	0.015222	3
BP	GO:0071383	4/80	206/18866	0.011451	0.030751	0.015222	4
BP	GO:0051961	5/80	319/18866	0.01147	0.030777	0.015235	5
BP	GO:0050866	4/80	207/18866	0.01164	0.031207	0.015448	4
BP	GO:0051149	3/80	111/18866	0.01173	0.031372	0.015529	3
BP	GO:0098732	3/80	111/18866	0.01173	0.031372	0.015529	3
BP	GO:1903509	3/80	111/18866	0.01173	0.031372	0.015529	3
BP	GO:0061458	6/80	447/18866	0.011771	0.031455	0.015571	6
BP	GO:0061013	4/80	208/18866	0.01183	0.031531	0.015608	4
BP	GO:0072522	4/80	208/18866	0.01183	0.031531	0.015608	4
BP	GO:0007431	2/80	39/18866	0.011887	0.031531	0.015608	2
BP	GO:0010831	2/80	39/18866	0.011887	0.031531	0.015608	2
BP	GO:0014037	2/80	39/18866	0.011887	0.031531	0.015608	2
BP	GO:0032660	2/80	39/18866	0.011887	0.031531	0.015608	2
BP	GO:0046326	2/80	39/18866	0.011887	0.031531	0.015608	2
BP	GO:0051931	2/80	39/18866	0.011887	0.031531	0.015608	2
BP	GO:1901068	2/80	39/18866	0.011887	0.031531	0.015608	2

BP	GO:0007204	5/80	322/18866	0.011906	0.031555	0.01562	5
BP	GO:0099565	3/80	112/18866	0.012017	0.031812	0.015747	3
BP	GO:0035303	4/80	209/18866	0.012023	0.031812	0.015747	4
BP	GO:0051235	5/80	324/18866	0.012203	0.032248	0.015963	5
BP	GO:0002377	4/80	210/18866	0.012218	0.032248	0.015963	4
BP	GO:0030100	4/80	210/18866	0.012218	0.032248	0.015963	4
BP	GO:1903555	3/80	113/18866	0.012308	0.032459	0.016067	3
BP	GO:0031396	4/80	211/18866	0.012415	0.032714	0.016194	4
BP	GO:0010613	2/80	40/18866	0.012479	0.03275	0.016212	2
BP	GO:0010939	2/80	40/18866	0.012479	0.03275	0.016212	2
BP	GO:0030501	2/80	40/18866	0.012479	0.03275	0.016212	2
BP	GO:0045746	2/80	40/18866	0.012479	0.03275	0.016212	2
BP	GO:1902895	2/80	40/18866	0.012479	0.03275	0.016212	2
BP	GO:0008645	3/80	114/18866	0.012602	0.03302	0.016345	3
BP	GO:0016101	3/80	114/18866	0.012602	0.03302	0.016345	3
BP	GO:0006643	4/80	212/18866	0.012614	0.033022	0.016346	4
BP	GO:0006898	5/80	328/18866	0.012813	0.033467	0.016566	5
BP	GO:0007254	4/80	213/18866	0.012814	0.033467	0.016566	4
BP	GO:0045089	4/80	213/18866	0.012814	0.033467	0.016566	4
BP	GO:0006874	6/80	456/18866	0.012886	0.033612	0.016638	6
BP	GO:0006096	3/80	115/18866	0.012901	0.033612	0.016638	3
BP	GO:0031623	3/80	115/18866	0.012901	0.033612	0.016638	3
BP	GO:0014742	2/80	41/18866	0.013083	0.033976	0.016819	2
BP	GO:0051354	2/80	41/18866	0.013083	0.033976	0.016819	2
BP	GO:0070317	2/80	41/18866	0.013083	0.033976	0.016819	2
BP	GO:0072528	2/80	41/18866	0.013083	0.033976	0.016819	2
BP	GO:0001655	5/80	330/18866	0.013125	0.034058	0.016859	5
BP	GO:0006757	3/80	116/18866	0.013204	0.034152	0.016906	3
BP	GO:0015749	3/80	116/18866	0.013204	0.034152	0.016906	3
BP	GO:0032392	3/80	116/18866	0.013204	0.034152	0.016906	3
BP	GO:0060964	3/80	116/18866	0.013204	0.034152	0.016906	3
BP	GO:0070374	4/80	215/18866	0.013222	0.034173	0.016916	4
BP	GO:0000266	2/80	42/18866	0.0137	0.035294	0.017471	2
BP	GO:0098760	2/80	42/18866	0.0137	0.035294	0.017471	2
BP	GO:0098761	2/80	42/18866	0.0137	0.035294	0.017471	2
BP	GO:1901658	2/80	42/18866	0.0137	0.035294	0.017471	2
BP	GO:0034219	3/80	118/18866	0.013821	0.03555	0.017598	3
BP	GO:0050868	3/80	118/18866	0.013821	0.03555	0.017598	3
BP	GO:0010822	3/80	119/18866	0.014136	0.036303	0.01797	3
BP	GO:0071706	3/80	119/18866	0.014136	0.036303	0.01797	3
BP	GO:0002369	2/80	43/18866	0.014329	0.036681	0.018157	2
BP	GO:0010470	2/80	43/18866	0.014329	0.036681	0.018157	2
BP	GO:0050850	2/80	43/18866	0.014329	0.036681	0.018157	2
BP	GO:0150077	2/80	43/18866	0.014329	0.036681	0.018157	2
BP	GO:0060147	3/80	120/18866	0.014455	0.036945	0.018288	3
BP	GO:0060966	3/80	120/18866	0.014455	0.036945	0.018288	3
BP	GO:0055074	6/80	468/18866	0.014485	0.036992	0.018312	6
BP	GO:0010906	3/80	121/18866	0.014778	0.037711	0.018667	3
BP	GO:0017145	2/80	44/18866	0.014971	0.038051	0.018836	2
BP	GO:0032620	2/80	44/18866	0.014971	0.038051	0.018836	2
BP	GO:0032881	2/80	44/18866	0.014971	0.038051	0.018836	2
BP	GO:0033003	2/80	44/18866	0.014971	0.038051	0.018836	2
BP	GO:1905521	2/80	44/18866	0.014971	0.038051	0.018836	2
BP	GO:0010721	5/80	343/18866	0.015282	0.038811	0.019212	5
BP	GO:1903050	4/80	225/18866	0.015386	0.039045	0.019328	4
BP	GO:1904375	3/80	123/18866	0.015437	0.039144	0.019377	3
BP	GO:0010828	2/80	45/18866	0.015624	0.039433	0.01952	2
BP	GO:0031018	2/80	45/18866	0.015624	0.039433	0.01952	2

BP	GO:0032722	2/80	45/18866	0.015624	0.039433	0.01952	2
BP	GO:0034105	2/80	45/18866	0.015624	0.039433	0.01952	2
BP	GO:0035094	2/80	45/18866	0.015624	0.039433	0.01952	2
BP	GO:0070316	2/80	45/18866	0.015624	0.039433	0.01952	2
BP	GO:0022612	3/80	124/18866	0.015772	0.039744	0.019674	3
BP	GO:0046031	3/80	124/18866	0.015772	0.039744	0.019674	3
BP	GO:0034605	3/80	125/18866	0.016111	0.040568	0.020081	3
BP	GO:0005978	2/80	46/18866	0.01629	0.040826	0.02021	2
BP	GO:0009250	2/80	46/18866	0.01629	0.040826	0.02021	2
BP	GO:0032309	2/80	46/18866	0.01629	0.040826	0.02021	2
BP	GO:0032692	2/80	46/18866	0.01629	0.040826	0.02021	2
BP	GO:0045023	2/80	46/18866	0.01629	0.040826	0.02021	2
BP	GO:0051646	2/80	46/18866	0.01629	0.040826	0.02021	2
BP	GO:0048762	4/80	229/18866	0.01631	0.040845	0.020219	4
BP	GO:0009615	5/80	349/18866	0.016352	0.040919	0.020255	5
BP	GO:0001649	4/80	231/18866	0.016785	0.04197	0.020775	4
BP	GO:0008542	2/80	47/18866	0.016968	0.042263	0.02092	2
BP	GO:0030225	2/80	47/18866	0.016968	0.042263	0.02092	2
BP	GO:0032480	2/80	47/18866	0.016968	0.042263	0.02092	2
BP	GO:0042551	2/80	47/18866	0.016968	0.042263	0.02092	2
BP	GO:0042572	2/80	47/18866	0.016968	0.042263	0.02092	2
BP	GO:0050769	6/80	485/18866	0.016982	0.042264	0.020921	6
BP	GO:0014013	3/80	128/18866	0.017154	0.042628	0.021101	3
BP	GO:0034763	3/80	128/18866	0.017154	0.042628	0.021101	3
BP	GO:0071482	3/80	129/18866	0.01751	0.043479	0.021523	3
BP	GO:0009409	2/80	48/18866	0.017657	0.043543	0.021554	2
BP	GO:0033628	2/80	48/18866	0.017657	0.043543	0.021554	2
BP	GO:0043370	2/80	48/18866	0.017657	0.043543	0.021554	2
BP	GO:0051972	2/80	48/18866	0.017657	0.043543	0.021554	2
BP	GO:0060324	2/80	48/18866	0.017657	0.043543	0.021554	2
BP	GO:0070169	2/80	48/18866	0.017657	0.043543	0.021554	2
BP	GO:0110151	2/80	48/18866	0.017657	0.043543	0.021554	2
BP	GO:1904036	2/80	48/18866	0.017657	0.043543	0.021554	2
BP	GO:2001239	2/80	48/18866	0.017657	0.043543	0.021554	2
BP	GO:0001505	4/80	235/18866	0.017761	0.043752	0.021658	4
BP	GO:0016049	6/80	490/18866	0.017769	0.043752	0.021658	6
BP	GO:0051480	5/80	357/18866	0.017857	0.043935	0.021748	5
BP	GO:0072503	6/80	492/18866	0.018091	0.044477	0.022017	6
BP	GO:0006766	3/80	131/18866	0.018234	0.044794	0.022174	3
BP	GO:0032872	4/80	237/18866	0.018262	0.044829	0.022191	4
BP	GO:0002686	2/80	49/18866	0.018359	0.044896	0.022224	2
BP	GO:0031279	2/80	49/18866	0.018359	0.044896	0.022224	2
BP	GO:0070231	2/80	49/18866	0.018359	0.044896	0.022224	2
BP	GO:1900087	2/80	49/18866	0.018359	0.044896	0.022224	2
BP	GO:2000107	2/80	49/18866	0.018359	0.044896	0.022224	2
BP	GO:0019751	3/80	132/18866	0.018602	0.045457	0.022502	3
BP	GO:0006165	3/80	133/18866	0.018975	0.046184	0.022862	3
BP	GO:0032886	4/80	240/18866	0.01903	0.046184	0.022862	4
BP	GO:0070302	4/80	240/18866	0.01903	0.046184	0.022862	4
BP	GO:0001961	2/80	50/18866	0.019072	0.046184	0.022862	2
BP	GO:0007528	2/80	50/18866	0.019072	0.046184	0.022862	2
BP	GO:0010718	2/80	50/18866	0.019072	0.046184	0.022862	2
BP	GO:0014009	2/80	50/18866	0.019072	0.046184	0.022862	2
BP	GO:0030195	2/80	50/18866	0.019072	0.046184	0.022862	2
BP	GO:0050873	2/80	50/18866	0.019072	0.046184	0.022862	2
BP	GO:0071715	2/80	50/18866	0.019072	0.046184	0.022862	2
BP	GO:1901571	2/80	50/18866	0.019072	0.046184	0.022862	2
BP	GO:1904707	2/80	50/18866	0.019072	0.046184	0.022862	2

BP	GO:0034754	3/80	134/18866	0.019351	0.046756	0.023144	3
BP	GO:0045995	3/80	134/18866	0.019351	0.046756	0.023144	3
BP	GO:0046683	3/80	134/18866	0.019351	0.046756	0.023144	3
BP	GO:1903320	4/80	242/18866	0.019553	0.047208	0.023368	4
BP	GO:0002449	5/80	366/18866	0.019656	0.04737	0.023449	5
BP	GO:0032368	3/80	135/18866	0.019732	0.04737	0.023449	3
BP	GO:0046939	3/80	135/18866	0.019732	0.04737	0.023449	3
BP	GO:0002204	2/80	51/18866	0.019796	0.04737	0.023449	2
BP	GO:0002208	2/80	51/18866	0.019796	0.04737	0.023449	2
BP	GO:0032206	2/80	51/18866	0.019796	0.04737	0.023449	2
BP	GO:0035272	2/80	51/18866	0.019796	0.04737	0.023449	2
BP	GO:0045190	2/80	51/18866	0.019796	0.04737	0.023449	2
BP	GO:0071675	2/80	51/18866	0.019796	0.04737	0.023449	2
BP	GO:1900047	2/80	51/18866	0.019796	0.04737	0.023449	2
BP	GO:1902893	2/80	51/18866	0.019796	0.04737	0.023449	2
BP	GO:1903573	2/80	51/18866	0.019796	0.04737	0.023449	2
BP	GO:0009135	3/80	136/18866	0.020116	0.048065	0.023793	3
BP	GO:0009179	3/80	136/18866	0.020116	0.048065	0.023793	3
BP	GO:0045861	5/80	369/18866	0.020282	0.048424	0.023971	5
BP	GO:0045839	2/80	52/18866	0.020532	0.048806	0.024159	2
BP	GO:0048260	2/80	52/18866	0.020532	0.048806	0.024159	2
BP	GO:0071622	2/80	52/18866	0.020532	0.048806	0.024159	2
BP	GO:0090329	2/80	52/18866	0.020532	0.048806	0.024159	2
BP	GO:1903557	2/80	52/18866	0.020532	0.048806	0.024159	2
BP	GO:2000677	2/80	52/18866	0.020532	0.048806	0.024159	2
BP	GO:0000724	3/80	138/18866	0.020898	0.049639	0.024572	3
CC	GO:0005942	5/80	29/19559	1.11E-07	2.45E-05	1.49E-05	5
CC	GO:0019898	10/80	306/19559	4.75E-07	5.25E-05	3.20E-05	10
CC	GO:0061695	9/80	253/19559	9.17E-07	6.75E-05	4.12E-05	9
CC	GO:0031983	9/80	328/19559	7.66E-06	0.000423	0.000258	9
CC	GO:0070069	4/80	34/19559	1.10E-05	0.000484	0.000295	4
CC	GO:0005743	10/80	489/19559	2.99E-05	0.001102	0.000672	10
CC	GO:0034774	8/80	322/19559	5.12E-05	0.001121	0.000683	8
CC	GO:0060205	8/80	326/19559	5.59E-05	0.001121	0.000683	8
CC	GO:0000781	6/80	164/19559	5.71E-05	0.001121	0.000683	6
CC	GO:0070469	5/80	101/19559	5.88E-05	0.001121	0.000683	5
CC	GO:0045121	8/80	329/19559	5.96E-05	0.001121	0.000683	8
CC	GO:0098857	8/80	330/19559	6.09E-05	0.001121	0.000683	8
CC	GO:0045277	3/80	20/19559	7.14E-05	0.001214	0.00074	3
CC	GO:0098589	8/80	343/19559	7.97E-05	0.001258	0.000767	8
CC	GO:0098687	8/80	350/19559	9.17E-05	0.001325	0.000808	8
CC	GO:0042629	3/80	22/19559	9.59E-05	0.001325	0.000808	3
CC	GO:0101002	5/80	124/19559	0.000155	0.001907	0.001162	5
CC	GO:1904813	5/80	124/19559	0.000155	0.001907	0.001162	5
CC	GO:0005901	4/80	82/19559	0.000356	0.004144	0.002526	4
CC	GO:0098803	4/80	85/19559	0.000409	0.004515	0.002753	4
CC	GO:0005775	5/80	173/19559	0.000719	0.00726	0.004426	5
CC	GO:0000307	3/80	43/19559	0.000723	0.00726	0.004426	3
CC	GO:0000794	4/80	103/19559	0.000844	0.008109	0.004944	4
CC	GO:0098562	5/80	188/19559	0.001044	0.009614	0.005861	5
CC	GO:0009925	3/80	51/19559	0.001191	0.010129	0.006176	3
CC	GO:0044853	4/80	113/19559	0.001192	0.010129	0.006176	4
CC	GO:0005750	2/80	14/19559	0.001456	0.011494	0.007007	2
CC	GO:0045275	2/80	14/19559	0.001456	0.011494	0.007007	2
CC	GO:0031616	2/80	15/19559	0.001676	0.012365	0.007538	2
CC	GO:0000784	4/80	124/19559	0.001678	0.012365	0.007538	4
CC	GO:0061702	2/80	17/19559	0.002159	0.014535	0.008861	2
CC	GO:0072686	4/80	133/19559	0.002168	0.014535	0.008861	4

CC	GO:0000793	5/80	222/19559	0.00217	0.014535	0.008861	5
CC	GO:0031143	2/80	18/19559	0.002423	0.015747	0.0096	2
CC	GO:0005876	3/80	66/19559	0.002505	0.015817	0.009643	3
CC	GO:0031093	3/80	67/19559	0.002615	0.016052	0.009786	3
CC	GO:0045178	3/80	69/19559	0.002843	0.016982	0.010353	3
CC	GO:0005759	7/80	473/19559	0.003217	0.018709	0.011406	7
CC	GO:0098978	6/80	361/19559	0.00362	0.020513	0.012506	6
CC	GO:0005819	6/80	367/19559	0.003923	0.021673	0.013213	6
CC	GO:0009898	4/80	164/19559	0.004597	0.024536	0.014959	4
CC	GO:0099522	2/80	25/19559	0.004663	0.024536	0.014959	2
CC	GO:1902554	3/80	89/19559	0.005813	0.029875	0.018214	3
CC	GO:0031091	3/80	91/19559	0.006182	0.031051	0.018931	3
CC	GO:0030496	4/80	182/19559	0.006622	0.032522	0.019827	4
CC	GO:0005667	6/80	413/19559	0.006906	0.032991	0.020114	6
CC	GO:0005925	6/80	415/19559	0.007065	0.032991	0.020114	6
CC	GO:0043202	3/80	96/19559	0.007166	0.032991	0.020114	3
CC	GO:0000780	2/80	32/19559	0.007568	0.03379	0.020601	2
CC	GO:0030055	6/80	423/19559	0.007728	0.03379	0.020601	6
CC	GO:0031234	3/80	99/19559	0.007798	0.03379	0.020601	3
CC	GO:0032839	2/80	34/19559	0.008515	0.035926	0.021903	2
CC	GO:0005788	5/80	308/19559	0.008616	0.035926	0.021903	5
CC	GO:1902911	3/80	104/19559	0.008923	0.036516	0.022263	3
CC	GO:0051233	2/80	36/19559	0.009512	0.03822	0.023302	2
MF	GO:0004674	19/80	435/18352	2.75E-14	8.57E-12	6.34E-12	19
MF	GO:0004713	11/80	135/18352	1.54E-11	2.40E-09	1.77E-09	11
MF	GO:0035173	6/80	17/18352	6.75E-11	7.02E-09	5.19E-09	6
MF	GO:0004714	8/80	61/18352	2.23E-10	1.74E-08	1.28E-08	8
MF	GO:0016303	5/80	10/18352	3.44E-10	2.14E-08	1.58E-08	5
MF	GO:0035004	5/80	12/18352	1.07E-09	5.58E-08	4.12E-08	5
MF	GO:0019199	8/80	80/18352	2.05E-09	9.14E-08	6.75E-08	8
MF	GO:0016307	5/80	16/18352	5.83E-09	2.02E-07	1.49E-07	5
MF	GO:0052742	5/80	16/18352	5.83E-09	2.02E-07	1.49E-07	5
MF	GO:0004712	6/80	45/18352	4.03E-08	1.26E-06	9.30E-07	6
MF	GO:0020037	8/80	138/18352	1.53E-07	4.32E-06	3.19E-06	8
MF	GO:0019902	9/80	194/18352	1.66E-07	4.32E-06	3.19E-06	9
MF	GO:0046906	8/80	148/18352	2.63E-07	6.30E-06	4.66E-06	8
MF	GO:0051219	6/80	85/18352	1.89E-06	4.20E-05	3.11E-05	6
MF	GO:0005126	9/80	271/18352	2.71E-06	5.65E-05	4.17E-05	9
MF	GO:0019903	7/80	149/18352	3.91E-06	7.62E-05	5.63E-05	7
MF	GO:0016725	3/80	10/18352	9.36E-06	0.000172	0.000127	3
MF	GO:0016712	4/80	35/18352	1.58E-05	0.000274	0.000203	4
MF	GO:0008353	3/80	12/18352	1.71E-05	0.00028	0.000207	3
MF	GO:0008395	4/80	38/18352	2.21E-05	0.000344	0.000254	4
MF	GO:0019838	6/80	136/18352	2.84E-05	0.000422	0.000312	6
MF	GO:0042169	4/80	41/18352	3.00E-05	0.000425	0.000314	4
MF	GO:0004708	3/80	18/18352	6.21E-05	0.000842	0.000622	3
MF	GO:0004707	3/80	20/18352	8.62E-05	0.001121	0.000828	3
MF	GO:0009055	5/80	111/18352	0.000124	0.001546	0.001142	5
MF	GO:0070330	3/80	25/18352	0.000171	0.002054	0.001518	3
MF	GO:0004129	3/80	28/18352	0.000242	0.002599	0.00192	3
MF	GO:0015002	3/80	28/18352	0.000242	0.002599	0.00192	3
MF	GO:0016676	3/80	28/18352	0.000242	0.002599	0.00192	3
MF	GO:0004693	3/80	29/18352	0.000269	0.002619	0.001935	3
MF	GO:0016675	3/80	29/18352	0.000269	0.002619	0.001935	3
MF	GO:0097472	3/80	29/18352	0.000269	0.002619	0.001935	3
MF	GO:0030332	3/80	30/18352	0.000297	0.002813	0.002078	3
MF	GO:0002020	5/80	137/18352	0.000331	0.003034	0.002242	5
MF	GO:0019825	3/80	36/18352	0.000513	0.004477	0.003308	3

MF	GO:0005506	5/80	151/18352	0.000517	0.004477	0.003308	5
MF	GO:0005125	6/80	235/18352	0.000564	0.004756	0.003514	6
MF	GO:0001784	3/80	42/18352	0.00081	0.006604	0.00488	3
MF	GO:0043560	2/80	10/18352	0.000826	0.006604	0.00488	2
MF	GO:0004497	4/80	101/18352	0.000992	0.007549	0.005578	4
MF	GO:0016709	3/80	45/18352	0.000992	0.007549	0.005578	3
MF	GO:0004715	3/80	46/18352	0.001058	0.007859	0.005807	3
MF	GO:0017171	5/80	191/18352	0.001482	0.010752	0.007944	5
MF	GO:0045309	3/80	53/18352	0.001597	0.011326	0.008368	3
MF	GO:0035259	2/80	15/18352	0.001899	0.012882	0.009518	2
MF	GO:0097153	2/80	15/18352	0.001899	0.012882	0.009518	2
MF	GO:0008392	2/80	16/18352	0.002164	0.014368	0.010616	2
MF	GO:0005507	3/80	60/18352	0.002283	0.014838	0.010963	3
MF	GO:0008391	2/80	20/18352	0.003388	0.02079	0.015361	2
MF	GO:0070182	2/80	20/18352	0.003388	0.02079	0.015361	2
MF	GO:0016811	3/80	69/18352	0.003398	0.02079	0.015361	3
MF	GO:0042379	3/80	70/18352	0.003539	0.021237	0.015691	3
MF	GO:0004709	2/80	24/18352	0.004867	0.028651	0.021169	2
MF	GO:0016702	2/80	25/18352	0.005275	0.029588	0.021861	2
MF	GO:0048018	7/80	487/18352	0.005322	0.029588	0.021861	7
MF	GO:0035258	3/80	81/18352	0.005331	0.029588	0.021861	3
MF	GO:0008083	4/80	162/18352	0.0055	0.029588	0.021861	4
MF	GO:0016705	4/80	162/18352	0.0055	0.029588	0.021861	4
MF	GO:0030546	7/80	492/18352	0.005621	0.029634	0.021896	7
MF	GO:0016701	2/80	26/18352	0.005699	0.029634	0.021896	2
MF	GO:0004252	4/80	169/18352	0.006377	0.032618	0.0241	4
MF	GO:0051427	4/80	177/18352	0.007488	0.03768	0.027841	4
MF	GO:0008236	4/80	187/18352	0.009046	0.044255	0.032698	4
MF	GO:0001530	2/80	33/18352	0.009078	0.044255	0.032698	2
MF	GO:0008022	4/80	189/18352	0.009382	0.045032	0.033272	4
MF	GO:0016922	3/80	101/18352	0.009786	0.046261	0.034181	3
MF	GO:0008144	3/80	104/18352	0.010595	0.049336	0.036452	3
