# Supplementary material for: Identifying and Targeting Prediction of the PI3K-AKT Signaling Pathway in Drug-Induced Thrombocytopenia in Infected Patients Receiving Linezolid Therapy: A Network Pharmacology-Based Analysis
Source: J Healthc Eng. 2022 Oct 15;2022:2282351. doi: 10.1155/2022/2282351 (PMC9588367; doi:10.1155/2022/2282351)
Supplement: Supplementary Materials — Supplementary Table 1 and experimental dataset are provided for MCODE cluster analysis. Supplementary data files for all the figures are also provided in the supplementary materials. [file 2282351.f1.zip › Figure 7-KEGG data.pdf]

ID	GeneRatio	BgRatio	pvalue	p.adjust	qvalue	Count
hsa01521	14/82	79/8142	2.57E-14	5.71E-12	1.76E-12	14
hsa05230	13/82	70/8142	1.23E-13	1.36E-11	4.19E-12	13
hsa05212	13/82	76/8142	3.76E-13	2.78E-11	8.58E-12	13
hsa05417	18/82	215/8142	2.52E-12	1.16E-10	3.56E-11	18
hsa04151	22/82	354/8142	2.69E-12	1.16E-10	3.56E-11	22
hsa05235	13/82	89/8142	3.12E-12	1.16E-10	3.56E-11	13
hsa01522	13/82	98/8142	1.11E-11	3.52E-10	1.09E-10	13
hsa04933	13/82	100/8142	1.45E-11	4.01E-10	1.24E-10	13
hsa04625	13/82	104/8142	2.41E-11	5.95E-10	1.83E-10	13
hsa04068	14/82	131/8142	3.36E-11	7.45E-10	2.30E-10	14
hsa04066	13/82	109/8142	4.43E-11	8.94E-10	2.75E-10	13
hsa05208	17/82	223/8142	5.00E-11	9.25E-10	2.85E-10	17
hsa05167	16/82	194/8142	6.11E-11	9.81E-10	3.02E-10	16
hsa05135	14/82	137/8142	6.19E-11	9.81E-10	3.02E-10	14
hsa05223	11/82	72/8142	9.98E-11	1.48E-09	4.55E-10	11
hsa05164	15/82	171/8142	1.08E-10	1.50E-09	4.63E-10	15
hsa05215	12/82	97/8142	1.70E-10	2.22E-09	6.85E-10	12
hsa04218	14/82	156/8142	3.58E-10	4.41E-09	1.36E-09	14
hsa04660	12/82	104/8142	3.90E-10	4.56E-09	1.41E-09	12
hsa04014	16/82	232/8142	8.84E-10	9.81E-09	3.02E-09	16
hsa05221	10/82	67/8142	9.53E-10	1.01E-08	3.11E-09	10
hsa04664	10/82	68/8142	1.11E-09	1.12E-08	3.45E-09	10
hsa04917	10/82	70/8142	1.49E-09	1.44E-08	4.43E-09	10
hsa05218	10/82	72/8142	1.98E-09	1.83E-08	5.64E-09	10
hsa04914	11/82	102/8142	4.62E-09	3.94E-08	1.22E-08	11
hsa05142	11/82	102/8142	4.62E-09	3.94E-08	1.22E-08	11
hsa04630	13/82	162/8142	6.43E-09	5.10E-08	1.57E-08	13
hsa05161	13/82	162/8142	6.43E-09	5.10E-08	1.57E-08	13
hsa04012	10/82	85/8142	1.04E-08	7.96E-08	2.45E-08	10
hsa05415	14/82	203/8142	1.13E-08	8.39E-08	2.58E-08	14
hsa04668	11/82	112/8142	1.26E-08	8.92E-08	2.75E-08	11
hsa05205	14/82	205/8142	1.29E-08	8.92E-08	2.75E-08	14
hsa05224	12/82	147/8142	2.14E-08	1.44E-07	4.44E-08	12
hsa04935	11/82	119/8142	2.38E-08	1.56E-07	4.80E-08	11
hsa04932	12/82	155/8142	3.89E-08	2.46E-07	7.60E-08	12
hsa05163	14/82	225/8142	4.21E-08	2.59E-07	7.99E-08	14
hsa05160	12/82	157/8142	4.49E-08	2.69E-07	8.29E-08	12
hsa05214	9/82	75/8142	4.99E-08	2.92E-07	8.99E-08	9
hsa05171	14/82	232/8142	6.19E-08	3.52E-07	1.09E-07	14
hsa05213	8/82	58/8142	9.54E-08	5.29E-07	1.63E-07	8
hsa04659	10/82	108/8142	1.07E-07	5.78E-07	1.78E-07	10
hsa04370	8/82	59/8142	1.09E-07	5.78E-07	1.78E-07	8
hsa05418	11/82	139/8142	1.20E-07	6.20E-07	1.91E-07	11
hsa04015	13/82	210/8142	1.43E-07	7.24E-07	2.23E-07	13
hsa05132	14/82	249/8142	1.49E-07	7.29E-07	2.25E-07	14
hsa05145	10/82	112/8142	1.51E-07	7.29E-07	2.25E-07	10
hsa05210	9/82	86/8142	1.67E-07	7.90E-07	2.43E-07	9
hsa04072	11/82	148/8142	2.28E-07	1.06E-06	3.26E-07	11
hsa05226	11/82	149/8142	2.45E-07	1.11E-06	3.42E-07	11
hsa04613	12/82	190/8142	3.66E-07	1.63E-06	5.01E-07	12

hsa05231	9/82	98/8142	5.19E-07	2.26E-06	6.96E-07	9
hsa04510	12/82	201/8142	6.72E-07	2.87E-06	8.84E-07	12
hsa05169	12/82	202/8142	7.08E-07	2.97E-06	9.14E-07	12
hsa05133	8/82	76/8142	8.10E-07	3.33E-06	1.03E-06	8
hsa04620	9/82	104/8142	8.63E-07	3.48E-06	1.07E-06	9
hsa05010	16/82	384/8142	1.05E-06	4.16E-06	1.28E-06	16
hsa05162	10/82	139/8142	1.14E-06	4.45E-06	1.37E-06	10
hsa05170	12/82	212/8142	1.19E-06	4.54E-06	1.40E-06	12
hsa04550	10/82	143/8142	1.48E-06	5.58E-06	1.72E-06	10
hsa05152	11/82	180/8142	1.63E-06	6.02E-06	1.85E-06	11
hsa04071	9/82	119/8142	2.69E-06	9.79E-06	3.02E-06	9
hsa05020	13/82	273/8142	2.86E-06	1.02E-05	3.16E-06	13
hsa05219	6/82	41/8142	2.94E-06	1.04E-05	3.20E-06	6
hsa04062	11/82	192/8142	3.06E-06	1.06E-05	3.27E-06	11
hsa04380	9/82	128/8142	4.93E-06	1.68E-05	5.18E-06	9
hsa04926	9/82	129/8142	5.25E-06	1.75E-05	5.40E-06	9
hsa05211	7/82	69/8142	5.29E-06	1.75E-05	5.40E-06	7
hsa05131	12/82	247/8142	5.85E-06	1.90E-05	5.84E-06	12
hsa04930	6/82	46/8142	5.89E-06	1.90E-05	5.84E-06	6
hsa05225	10/82	168/8142	6.36E-06	2.02E-05	6.22E-06	10
hsa04010	13/82	294/8142	6.46E-06	2.02E-05	6.23E-06	13
hsa05207	11/82	212/8142	7.96E-06	2.45E-05	7.57E-06	11
hsa04210	9/82	136/8142	8.10E-06	2.46E-05	7.60E-06	9
hsa05144	6/82	50/8142	9.68E-06	2.90E-05	8.95E-06	6
hsa05220	7/82	76/8142	1.01E-05	3.00E-05	9.24E-06	7
hsa04140	9/82	141/8142	1.09E-05	3.18E-05	9.79E-06	9
hsa04931	8/82	108/8142	1.17E-05	3.36E-05	1.04E-05	8
hsa05166	11/82	222/8142	1.23E-05	3.51E-05	1.08E-05	11
hsa04725	8/82	113/8142	1.63E-05	4.58E-05	1.41E-05	8
hsa05022	16/82	476/8142	1.67E-05	4.60E-05	1.42E-05	16
hsa04662	7/82	82/8142	1.68E-05	4.60E-05	1.42E-05	7
hsa04722	8/82	119/8142	2.38E-05	6.44E-05	1.98E-05	8
hsa05143	5/82	37/8142	3.10E-05	8.30E-05	2.56E-05	5
hsa04611	8/82	124/8142	3.20E-05	8.47E-05	2.61E-05	8
hsa04213	6/82	62/8142	3.40E-05	8.88E-05	2.74E-05	6
hsa05203	10/82	204/8142	3.47E-05	8.97E-05	2.76E-05	10
hsa04929	6/82	64/8142	4.08E-05	0.0001035	3.19E-05	6
hsa04657	7/82	94/8142	4.10E-05	0.0001035	3.19E-05	7
hsa04650	8/82	131/8142	4.76E-05	0.0001187	3.66E-05	8
hsa04666	7/82	97/8142	5.02E-05	0.0001239	3.82E-05	7
hsa04910	8/82	137/8142	6.55E-05	0.0001597	4.92E-05	8
hsa01524	6/82	73/8142	8.62E-05	0.0002081	6.41E-05	6
hsa05017	8/82	143/8142	8.86E-05	0.0002113	6.51E-05	8
hsa04621	9/82	184/8142	8.95E-05	0.0002113	6.51E-05	9
hsa04217	8/82	159/8142	0.0001856	0.0004338	0.0001337	8
hsa04919	7/82	121/8142	0.0002034	0.0004704	0.000145	7
hsa04211	6/82	89/8142	0.000259	0.0005928	0.0001827	6
hsa04658	6/82	92/8142	0.0003103	0.0006958	0.0002145	6
hsa05222	6/82	92/8142	0.0003103	0.0006958	0.0002145	6
hsa05165	11/82	331/8142	0.0004475	0.0009934	0.0003062	11
hsa04915	7/82	138/8142	0.0004551	0.0010003	0.0003083	7

hsa05321	5/82	65/8142	0.0004715	0.0010262	0.0003163	5
hsa04960	4/82	37/8142	0.0004902	0.0010565	0.0003256	4
hsa00970	5/82	66/8142	0.0005061	0.0010804	0.000333	5
hsa05146	6/82	102/8142	0.000541	0.0011439	0.0003525	6
hsa04920	5/82	69/8142	0.0006217	0.001302	0.0004013	5
hsa04020	9/82	240/8142	0.0006498	0.0013482	0.0004155	9
hsa04520	5/82	71/8142	0.000709	0.0014574	0.0004492	5
hsa00562	5/82	73/8142	0.0008052	0.001625	0.0005008	5
hsa04115	5/82	73/8142	0.0008052	0.001625	0.0005008	5
hsa04150	7/82	156/8142	0.0009456	0.0018911	0.0005829	7
hsa04726	6/82	115/8142	0.0010192	0.0020166	0.0006215	6
hsa05140	5/82	77/8142	0.0010265	0.0020166	0.0006215	5
hsa05206	10/82	310/8142	0.0010398	0.0020249	0.0006241	10
hsa04973	4/82	47/8142	0.0012265	0.0023677	0.0007297	4
hsa04714	8/82	232/8142	0.0022341	0.0042757	0.0013178	8
hsa04923	4/82	56/8142	0.0023584	0.0044749	0.0013792	4
hsa04912	5/82	93/8142	0.0023822	0.0044817	0.0013813	5
hsa05134	4/82	57/8142	0.0025172	0.0046959	0.0014473	4
hsa04371	6/82	139/8142	0.002687	0.004971	0.0015321	6
hsa00591	3/82	29/8142	0.0029788	0.0054472	0.0016788	3
hsa04750	5/82	98/8142	0.0029935	0.0054472	0.0016788	5
hsa04640	5/82	99/8142	0.0031282	0.005646	0.0017401	5
hsa05014	10/82	364/8142	0.0034101	0.0061052	0.0018817	10
hsa05130	7/82	197/8142	0.003584	0.0063652	0.0019618	7
hsa05012	8/82	266/8142	0.0051481	0.0090705	0.0027956	8
hsa05120	4/82	70/8142	0.0052941	0.0092542	0.0028522	4
hsa00982	4/82	72/8142	0.0058509	0.0101476	0.0031275	4
hsa04810	7/82	218/8142	0.0062183	0.0107013	0.0032982	7
hsa04024	7/82	221/8142	0.006689	0.0114227	0.0035205	7
hsa04152	5/82	120/8142	0.0070767	0.0119925	0.0036961	5
hsa05100	4/82	77/8142	0.0074106	0.0124632	0.0038412	4
hsa04110	5/82	126/8142	0.0086552	0.0144471	0.0044526	5
hsa04360	6/82	182/8142	0.0098827	0.0163728	0.0050461	6
hsa04114	5/82	131/8142	0.0101452	0.0166833	0.0051418	5
hsa05168	11/82	495/8142	0.0103899	0.0169137	0.0052129	11
hsa04610	4/82	85/8142	0.0104377	0.0169137	0.0052129	4
hsa00190	5/82	134/8142	0.0111193	0.0178875	0.005513	5
hsa04260	4/82	87/8142	0.0113027	0.0180518	0.0055636	4
hsa05016	8/82	306/8142	0.0115928	0.0183829	0.0056657	8
hsa04540	4/82	88/8142	0.011752	0.0185032	0.0057027	4
hsa05202	6/82	193/8142	0.0129456	0.0202388	0.0062377	6
hsa04936	5/82	142/8142	0.0140256	0.021774	0.0067108	5
hsa05323	4/82	93/8142	0.0141708	0.0218466	0.0067332	4
hsa04070	4/82	97/8142	0.016318	0.0249835	0.0077	4
hsa04061	4/82	100/8142	0.0180558	0.0274547	0.0084616	4
hsa04921	5/82	154/8142	0.0192835	0.0291221	0.0089755	5
hsa04928	4/82	106/8142	0.0218674	0.0328011	0.0101094	4
hsa00590	3/82	61/8142	0.023316	0.0347393	0.0107067	3
hsa04623	3/82	63/8142	0.0253626	0.0375366	0.0115689	3
hsa04022	5/82	167/8142	0.0262921	0.0386546	0.0119135	5
hsa04670	4/82	114/8142	0.0276656	0.0404064	0.0124534	4

hsa05204	3/82	69/8142	0.0320649	0.0465256	0.0143393		3
hsa04622	3/82	70/8142	0.0332636	0.0479514	0.0147788		3
