# Supplementary material for: Identifying and Targeting Prediction of the PI3K-AKT Signaling Pathway in Drug-Induced Thrombocytopenia in Infected Patients Receiving Linezolid Therapy: A Network Pharmacology-Based Analysis
Source: J Healthc Eng. 2022 Oct 15;2022:2282351. doi: 10.1155/2022/2282351 (PMC9588367; doi:10.1155/2022/2282351)
Supplement: Supplementary Materials — Supplementary Table 1 and experimental dataset are provided for MCODE cluster analysis. Supplementary data files for all the figures are also provided in the supplementary materials. [file 2282351.f1.zip › Figure 8-Drug-target-pathway network data.pdf]

EGFR tyrosine kinase inhibitor resistance	IL6
EGFR tyrosine kinase inhibitor resistance	STAT3
EGFR tyrosine kinase inhibitor resistance	KDR
EGFR tyrosine kinase inhibitor resistance	PIK3CB
EGFR tyrosine kinase inhibitor resistance	MET
EGFR tyrosine kinase inhibitor resistance	RAF1
EGFR tyrosine kinase inhibitor resistance	JAK2
EGFR tyrosine kinase inhibitor resistance	PIK3CD
EGFR tyrosine kinase inhibitor resistance	PIK3CA
EGFR tyrosine kinase inhibitor resistance	MAPK1
EGFR tyrosine kinase inhibitor resistance	AKT1
EGFR tyrosine kinase inhibitor resistance	ERBB2
EGFR tyrosine kinase inhibitor resistance	EGFR
EGFR tyrosine kinase inhibitor resistance	MTOR
Central carbon metabolism in cancer	PIK3CB
Central carbon metabolism in cancer	MET
Central carbon metabolism in cancer	RAF1
Central carbon metabolism in cancer	PIK3CD
Central carbon metabolism in cancer	PIK3CA
Central carbon metabolism in cancer	MAPK1
Central carbon metabolism in cancer	AKT1
Central carbon metabolism in cancer	FGFR1
Central carbon metabolism in cancer	FLT3
Central carbon metabolism in cancer	ERBB2
Central carbon metabolism in cancer	EGFR
Central carbon metabolism in cancer	MTOR
Central carbon metabolism in cancer	KIT
Pancreatic cancer	STAT3
Pancreatic cancer	PIK3CB
Pancreatic cancer	RAF1
Pancreatic cancer	PIK3CD
Pancreatic cancer	PIK3CA
Pancreatic cancer	MAPK1
Pancreatic cancer	AKT1
Pancreatic cancer	ERBB2
Pancreatic cancer	EGFR
Pancreatic cancer	MTOR
Pancreatic cancer	CDK4
Pancreatic cancer	MAPK8
Pancreatic cancer	RAD51
Lipid and atherosclerosis	CYP2C9
Lipid and atherosclerosis	IL6
Lipid and atherosclerosis	CCL2
Lipid and atherosclerosis	NLRP3

Lipid and atherosclerosis	HSPD1
Lipid and atherosclerosis	STAT3
Lipid and atherosclerosis	MAPK14
Lipid and atherosclerosis	PIK3CB
Lipid and atherosclerosis	JAK2
Lipid and atherosclerosis	PIK3CD
Lipid and atherosclerosis	PIK3CA
Lipid and atherosclerosis	MAPK1
Lipid and atherosclerosis	TBK1
Lipid and atherosclerosis	CASP7
Lipid and atherosclerosis	AKT1
Lipid and atherosclerosis	CASP1
Lipid and atherosclerosis	SELE
Lipid and atherosclerosis	MAPK8
PI3K-Akt signaling pathway	IL6
PI3K-Akt signaling pathway	EPO
PI3K-Akt signaling pathway	KDR
PI3K-Akt signaling pathway	PIK3CB
PI3K-Akt signaling pathway	SYK
PI3K-Akt signaling pathway	MET
PI3K-Akt signaling pathway	RAF1
PI3K-Akt signaling pathway	JAK2
PI3K-Akt signaling pathway	PIK3CD
PI3K-Akt signaling pathway	PIK3CG
PI3K-Akt signaling pathway	PIK3CA
PI3K-Akt signaling pathway	MAPK1
PI3K-Akt signaling pathway	AKT1
PI3K-Akt signaling pathway	FLT1
PI3K-Akt signaling pathway	FGFR1
PI3K-Akt signaling pathway	FLT3
PI3K-Akt signaling pathway	CDK2
PI3K-Akt signaling pathway	ERBB2
PI3K-Akt signaling pathway	EGFR
PI3K-Akt signaling pathway	MTOR
PI3K-Akt signaling pathway	CDK4
PI3K-Akt signaling pathway	KIT
PD-L1 expression and PD-1 checkpoint pathway in cancer	IFNG
PD-L1 expression and PD-1 checkpoint pathway in cancer	STAT3
PD-L1 expression and PD-1 checkpoint pathway in cancer	MAPK14
PD-L1 expression and PD-1 checkpoint pathway in cancer	PIK3CB
PD-L1 expression and PD-1 checkpoint pathway in cancer	RAF1
PD-L1 expression and PD-1 checkpoint pathway in cancer	JAK2
PD-L1 expression and PD-1 checkpoint pathway in cancer	PIK3CD
PD-L1 expression and PD-1 checkpoint pathway in cancer	PIK3CA

PD-L1 expression and PD-1 checkpoint pathway in cancer	MAPK1
PD-L1 expression and PD-1 checkpoint pathway in cancer	AKT1
PD-L1 expression and PD-1 checkpoint pathway in cancer	EGFR
PD-L1 expression and PD-1 checkpoint pathway in cancer	MTOR
PD-L1 expression and PD-1 checkpoint pathway in cancer	ZAP70
Endocrine resistance	CYP2D6
Endocrine resistance	MAPK14
Endocrine resistance	PIK3CB
Endocrine resistance	RAF1
Endocrine resistance	PIK3CD
Endocrine resistance	PIK3CA
Endocrine resistance	MAPK1
Endocrine resistance	AKT1
Endocrine resistance	ERBB2
Endocrine resistance	EGFR
Endocrine resistance	MTOR
Endocrine resistance	CDK4
Endocrine resistance	MAPK8
AGE-RAGE signaling pathway in diabetic complications	IL6
AGE-RAGE signaling pathway in diabetic complications	CCL2
AGE-RAGE signaling pathway in diabetic complications	STAT3
AGE-RAGE signaling pathway in diabetic complications	MAPK14
AGE-RAGE signaling pathway in diabetic complications	PIK3CB
AGE-RAGE signaling pathway in diabetic complications	JAK2
AGE-RAGE signaling pathway in diabetic complications	PIK3CD
AGE-RAGE signaling pathway in diabetic complications	PIK3CA
AGE-RAGE signaling pathway in diabetic complications	MAPK1
AGE-RAGE signaling pathway in diabetic complications	AKT1
AGE-RAGE signaling pathway in diabetic complications	CDK4
AGE-RAGE signaling pathway in diabetic complications	SELE
AGE-RAGE signaling pathway in diabetic complications	MAPK8
C-type lectin receptor signaling pathway	IL6
C-type lectin receptor signaling pathway	IL10
C-type lectin receptor signaling pathway	NLRP3
C-type lectin receptor signaling pathway	MAPK14
C-type lectin receptor signaling pathway	PIK3CB
C-type lectin receptor signaling pathway	SYK
C-type lectin receptor signaling pathway	RAF1
C-type lectin receptor signaling pathway	PIK3CD
C-type lectin receptor signaling pathway	PIK3CA
C-type lectin receptor signaling pathway	MAPK1
C-type lectin receptor signaling pathway	AKT1
C-type lectin receptor signaling pathway	CASP1
C-type lectin receptor signaling pathway	MAPK8

FoxO signaling pathway	IL6
FoxO signaling pathway	IL10
FoxO signaling pathway	CAT
FoxO signaling pathway	STAT3
FoxO signaling pathway	MAPK14
FoxO signaling pathway	PIK3CB
FoxO signaling pathway	RAF1
FoxO signaling pathway	PIK3CD
FoxO signaling pathway	PIK3CA
FoxO signaling pathway	MAPK1
FoxO signaling pathway	AKT1
FoxO signaling pathway	CDK2
FoxO signaling pathway	EGFR
FoxO signaling pathway	MAPK8
HIF-1 signaling pathway	IL6
HIF-1 signaling pathway	IFNG
HIF-1 signaling pathway	EPO
HIF-1 signaling pathway	STAT3
HIF-1 signaling pathway	PIK3CB
HIF-1 signaling pathway	PIK3CD
HIF-1 signaling pathway	PIK3CA
HIF-1 signaling pathway	MAPK1
HIF-1 signaling pathway	AKT1
HIF-1 signaling pathway	FLT1
HIF-1 signaling pathway	ERBB2
HIF-1 signaling pathway	EGFR
HIF-1 signaling pathway	MTOR
Chemical carcinogenesis - reactive oxygen species	COX2
Chemical carcinogenesis - reactive oxygen species	SLC25A4
Chemical carcinogenesis - reactive oxygen species	CAT
Chemical carcinogenesis - reactive oxygen species	COX1
Chemical carcinogenesis - reactive oxygen species	ND6
Chemical carcinogenesis - reactive oxygen species	COX3
Chemical carcinogenesis - reactive oxygen species	CYTB
Chemical carcinogenesis - reactive oxygen species	MAPK14
Chemical carcinogenesis - reactive oxygen species	PIK3CB
Chemical carcinogenesis - reactive oxygen species	MET
Chemical carcinogenesis - reactive oxygen species	RAF1
Chemical carcinogenesis - reactive oxygen species	PIK3CD
Chemical carcinogenesis - reactive oxygen species	PIK3CA
Chemical carcinogenesis - reactive oxygen species	MAPK1
Chemical carcinogenesis - reactive oxygen species	AKT1
Chemical carcinogenesis - reactive oxygen species	EGFR
Chemical carcinogenesis - reactive oxygen species	MAPK8

Kaposi sarcoma-associated herpesvirus infection	IL6
Kaposi sarcoma-associated herpesvirus infection	STAT3
Kaposi sarcoma-associated herpesvirus infection	MAPK14
Kaposi sarcoma-associated herpesvirus infection	PIK3CB
Kaposi sarcoma-associated herpesvirus infection	SYK
Kaposi sarcoma-associated herpesvirus infection	RAF1
Kaposi sarcoma-associated herpesvirus infection	JAK2
Kaposi sarcoma-associated herpesvirus infection	PIK3CD
Kaposi sarcoma-associated herpesvirus infection	PIK3CG
Kaposi sarcoma-associated herpesvirus infection	PIK3CA
Kaposi sarcoma-associated herpesvirus infection	MAPK1
Kaposi sarcoma-associated herpesvirus infection	TBK1
Kaposi sarcoma-associated herpesvirus infection	AKT1
Kaposi sarcoma-associated herpesvirus infection	MTOR
Kaposi sarcoma-associated herpesvirus infection	CDK4
Kaposi sarcoma-associated herpesvirus infection	MAPK8
Yersinia infection	IL6
Yersinia infection	CCL2
Yersinia infection	IL10
Yersinia infection	NLRP3
Yersinia infection	MAPK14
Yersinia infection	PIK3CB
Yersinia infection	PIK3CD
Yersinia infection	PIK3CA
Yersinia infection	MAPK1
Yersinia infection	TBK1
Yersinia infection	AKT1
Yersinia infection	CASP1
Yersinia infection	ZAP70
Yersinia infection	MAPK8
Non-small cell lung cancer	STAT3
Non-small cell lung cancer	PIK3CB
Non-small cell lung cancer	MET
Non-small cell lung cancer	RAF1
Non-small cell lung cancer	PIK3CD
Non-small cell lung cancer	PIK3CA
Non-small cell lung cancer	MAPK1
Non-small cell lung cancer	AKT1
Non-small cell lung cancer	ERBB2
Non-small cell lung cancer	EGFR
Non-small cell lung cancer	CDK4
Influenza A	IL6
Influenza A	IFNG
Influenza A	CCL2

Influenza A	SLC25A4
Influenza A	NLRP3
Influenza A	PIK3CB
Influenza A	RAF1
Influenza A	JAK2
Influenza A	PIK3CD
Influenza A	PIK3CA
Influenza A	MAPK1
Influenza A	TBK1
Influenza A	AKT1
Influenza A	CASP1
Influenza A	CDK4
Prostate cancer	PIK3CB
Prostate cancer	RAF1
Prostate cancer	PIK3CD
Prostate cancer	PIK3CA
Prostate cancer	PLAT
Prostate cancer	MAPK1
Prostate cancer	AKT1
Prostate cancer	FGFR1
Prostate cancer	CDK2
Prostate cancer	ERBB2
Prostate cancer	EGFR
Prostate cancer	MTOR
Cellular senescence	IL6
Cellular senescence	SLC25A4
Cellular senescence	MAPK14
Cellular senescence	PIK3CB
Cellular senescence	CHEK1
Cellular senescence	RAF1
Cellular senescence	PIK3CD
Cellular senescence	PIK3CA
Cellular senescence	MAPK1
Cellular senescence	AKT1
Cellular senescence	CDK2
Cellular senescence	MTOR
Cellular senescence	CDK1
Cellular senescence	CDK4
T cell receptor signaling pathway	IFNG
T cell receptor signaling pathway	IL10
T cell receptor signaling pathway	MAPK14
T cell receptor signaling pathway	PIK3CB
T cell receptor signaling pathway	RAF1
T cell receptor signaling pathway	PIK3CD

T cell receptor signaling pathway	PIK3CA
T cell receptor signaling pathway	MAPK1
T cell receptor signaling pathway	AKT1
T cell receptor signaling pathway	CDK4
T cell receptor signaling pathway	ZAP70
T cell receptor signaling pathway	MAPK8
Ras signaling pathway	KDR
Ras signaling pathway	PIK3CB
Ras signaling pathway	MET
Ras signaling pathway	RAF1
Ras signaling pathway	PIK3CD
Ras signaling pathway	PIK3CA
Ras signaling pathway	MAPK1
Ras signaling pathway	TBK1
Ras signaling pathway	AKT1
Ras signaling pathway	FLT1
Ras signaling pathway	FGFR1
Ras signaling pathway	FLT3
Ras signaling pathway	EGFR
Ras signaling pathway	ZAP70
Ras signaling pathway	KIT
Ras signaling pathway	MAPK8
Linezolid	CYP3A4
Linezolid	CYP2D6
Linezolid	CYP2C9
Linezolid	F5
Linezolid	POLG
Linezolid	TYMP
Linezolid	ALB
Linezolid	IL6
Linezolid	COX2
Linezolid	PIK3C2A
Linezolid	CYP2C19
Linezolid	IFNG
Linezolid	CCL2
Linezolid	IL10
Linezolid	RRM2B
Linezolid	SLC25A4
Linezolid	DGUOK
Linezolid	FANCI
Linezolid	MPV17
Linezolid	TWNK
Linezolid	TRNM
Linezolid	PPBP

Linezolid	NLRP3
Linezolid	TRNL1
Linezolid	TRNK
Linezolid	TRNF
Linezolid	CAT
Linezolid	HSPD1
Linezolid	EPO
Linezolid	COX1
Linezolid	ND6
Linezolid	COX3
Linezolid	CYTB
Linezolid	TRNS2
Linezolid	F10
Linezolid	STAT3
Linezolid	GBA
Linezolid	MAPK14
Linezolid	PRKDC
Linezolid	RORC
Linezolid	KDR
Linezolid	PIK3CB
Linezolid	CHEK1
Linezolid	P2RX7
Linezolid	SYK
Linezolid	MET
Linezolid	LRRK2
Linezolid	RAF1
Linezolid	JAK2
Linezolid	PARP1
Linezolid	ASAH1
Linezolid	CNR2
Linezolid	PIK3CD
Linezolid	PIK3CG
Linezolid	PIK3CA
Linezolid	ALOX5
Linezolid	PLAT
Linezolid	KLKB1
Linezolid	MAPK1
Linezolid	CD38
Linezolid	TBK1
Linezolid	CASP7
Linezolid	AKT1
Linezolid	CASP1
Linezolid	PABPC1
Linezolid	FLT1

Linezolid FGFR1
Linezolid FLT3
Linezolid ACHE
Linezolid ELANE
Linezolid CDK2
Linezolid IDO1
Linezolid ERBB2
Linezolid EGFR
Linezolid MTOR
Linezolid AURKB
Linezolid CDK1
Linezolid CDK4
Linezolid NAAA
Linezolid AURKA
Linezolid ZAP70
Linezolid SELE
Linezolid KIT
Linezolid MAPK8
Linezolid RAD51
Thrombocytopenia CYP3A4
Thrombocytopenia CYP2D6
Thrombocytopenia CYP2C9
Thrombocytopenia F5
Thrombocytopenia POLG
Thrombocytopenia TYMP
Thrombocytopenia ALB
Thrombocytopenia IL6
Thrombocytopenia COX2
Thrombocytopenia PIK3C2A
Thrombocytopenia CYP2C19
Thrombocytopenia IFNG
Thrombocytopenia CCL2
Thrombocytopenia IL10
Thrombocytopenia RRM2B
Thrombocytopenia SLC25A4
Thrombocytopenia DGUOK
Thrombocytopenia FANCI
Thrombocytopenia MPV17
Thrombocytopenia TWNK
Thrombocytopenia TRNM
Thrombocytopenia PPBP
Thrombocytopenia NLRP3
Thrombocytopenia TRNL1
Thrombocytopenia TRNK

Thrombocytopenia	TRNF
Thrombocytopenia	CAT
Thrombocytopenia	HSPD1
Thrombocytopenia	EPO
Thrombocytopenia	COX1
Thrombocytopenia	ND6
Thrombocytopenia	COX3
Thrombocytopenia	CYTB
Thrombocytopenia	TRNS2
Thrombocytopenia	F10
Thrombocytopenia	STAT3
Thrombocytopenia	GBA
Thrombocytopenia	MAPK14
Thrombocytopenia	PRKDC
Thrombocytopenia	RORC
Thrombocytopenia	KDR
Thrombocytopenia	PIK3CB
Thrombocytopenia	CHEK1
Thrombocytopenia	P2RX7
Thrombocytopenia	SYK
Thrombocytopenia	MET
Thrombocytopenia	LRRK2
Thrombocytopenia	RAF1
Thrombocytopenia	JAK2
Thrombocytopenia	PARP1
Thrombocytopenia	ASAH1
Thrombocytopenia	CNR2
Thrombocytopenia	PIK3CD
Thrombocytopenia	PIK3CG
Thrombocytopenia	PIK3CA
Thrombocytopenia	ALOX5
Thrombocytopenia	PLAT
Thrombocytopenia	KLKB1
Thrombocytopenia	MAPK1
Thrombocytopenia	CD38
Thrombocytopenia	TBK1
Thrombocytopenia	CASP7
Thrombocytopenia	AKT1
Thrombocytopenia	CASP1
Thrombocytopenia	PABPC1
Thrombocytopenia	FLT1
Thrombocytopenia	FGFR1
Thrombocytopenia	FLT3
Thrombocytopenia	ACHE

Thrombocytopenia	ELANE
Thrombocytopenia	CDK2
Thrombocytopenia	IDO1
Thrombocytopenia	ERBB2
Thrombocytopenia	EGFR
Thrombocytopenia	MTOR
Thrombocytopenia	AURKB
Thrombocytopenia	CDK1
Thrombocytopenia	CDK4
Thrombocytopenia	NAAA
Thrombocytopenia	AURKA
Thrombocytopenia	ZAP70
Thrombocytopenia	SELE
Thrombocytopenia	KIT
Thrombocytopenia	MAPK8
Thrombocytopenia	RAD51
