# Supplementary material for: Identifying and Targeting Prediction of the PI3K-AKT Signaling Pathway in Drug-Induced Thrombocytopenia in Infected Patients Receiving Linezolid Therapy: A Network Pharmacology-Based Analysis
Source: J Healthc Eng. 2022 Oct 15;2022:2282351. doi: 10.1155/2022/2282351 (PMC9588367; doi:10.1155/2022/2282351)
Supplement: Supplementary Materials — Supplementary Table 1 and experimental dataset are provided for MCODE cluster analysis. Supplementary data files for all the figures are also provided in the supplementary materials. [file 2282351.f1.zip › Figures 1-3 disease data.pdf]

MPL
GATA1
WAS
ANKRD26
THPO
RUNX1
ITGB3
ETV6
MYH9
GP1BA
ITGA2B
RBM8A
GP1BB
HOXA11
MECOM
CYCS
ADAMTS13
MASTL
FLI1
VWF
SLFN14
TCPT
ITGA2
SRC
FYB1
LIX1L-AS1
GFI1B
NBEAL2
GBA
IKZF5
ACTN1
WDR1
DIAPH1
GP9
THBD
MPIG6B
PF4
LOC106627981
TPP2
SEPT5-GP1BB
FCGR2A
F3
CFH
DGKE

WIPF1
CD46
FOXP3
JAK2
IL10
CD109
IL11
C3
FAS
FLT3
TUBB1
TERT
STAT3
CFI
SOCS1
CTLA4
NPM1
FCGR2C
KRAS
ARPC1B
FASLG
F2
F11
CD40LG
SELP
CASP10
KIT
P2RY12
STIM1
SAMD9L
SMAD4
SBDS
GP6
FCGR2B
TNFRSF13B
CFHR1
CHEK2
CFHR3
IL3
KCNE2
SALL4
CDC42
SERPINC1
CD36

CD19
MED12L
CSF3
GATA2
HLA-DRB1
CFHR5
TFRC
STAT1
IL6
RNASEH2B
MMACHC
TNF
IFIH1
TERC
ERCC6L2
ZFPM1
F10
PTPN11
ITK
ACBD5
SAMHD1
APOE
LRBA
TNFRSF13C
RNASEH2A
FLNA
ETS1
CFB
GNE
MIR155
CD4
F5
TP53
ABCG5
RNASEH2C
NOP10
IFNA2
FCGR3A
PRKCD
STAT2
CSF2
SMPD1
STXBP2
APOH

PRKACG
ABCG8
TET2
GP5
IFNG
CBFB
TNFSF13B
RASGRP1
CTC1
PLAT
PDGFRA
RPS19
JBS
TREX1
CD209
ADAR
IL2
PRF1
ACP5
CFHR4
EPO
EFCAB13-DT
COL4A5
HP
ADA
IFNA1
HLA-DRB3
ACTB
CXCL8
CD34
F8
ALB
TNFAIP3
CR2
CALR
IL18
LYST
KMT2A
NFKB2
DNMT3A
TINF2
CD79A
ICAM1
CD81

PTPN22
EFL1
DKC1
MS4A1
HLA-B
SYK
CEBPA
CCR6
KITLG
NRAS
BAAT
LIG4
IL1B
PAX5
NFKB1
TGFB1
MYSM1
ICOSLG
TLR4
USP18
ATM
BCR
MAPK1
NBN
ZAP70
CBL
RPL35A
PSMB4
PECAM1
SAMD9
ATRIP
RASGRP2
PTPRJ
BRAF
SERPINE1
CD8A
ENG
G6PC3
IL17A
MTHFR
RTEL1
ORAI1
RHD
CCL2

PPBP
ITGAM
PDCD1
ATRIP-TREX1
DNAJC21
NHP2
CXCL12
FGA
ABCB1
GJB2
IL1A
FCGR1A
BLK
ANXA5
OSTM1
HLA-A
RFXANK
IL4
ZNF85
ICOS
TNFSF12
NUP214
IL7R
STN1
FANCA
MBL2
CXCL10
STAT5B
MIR223
U2AF1
C1GALT1C1
THBS1
MYH7
CRP
IL1RN
TLR2
CRKL
FYN
FANCC
F2R
PICALM
MLLT10
PSMB9
ITGAL

GPT
PNP
F7
CASP3
FANCD2
ACP1
CIITA
FANCF
ALG8
RAG2
WFS1
SELL
SRP54
CHIC2
PLG
SLX4
FANCB
MIR126
GATA3
MIR15A
IL7
BRCA2
APBB1
HAVCR2
SLC17A5
F9
SPATA5
IFNB1
LYN
PCCA
BRCA1
SELE
MAP2K1
FANCG
KLF1
CD47
FIP1L1
AZU1
TBX1
ACVRL1
FANCE
TLR7
TALDO1
SETBP1

LPP
SH3GL1
IFNG-AS1
BTK
IGHG1
TLR9
MAP2K2
LUC7L2
TOP1
TBX5
ERBB2
COMT
MAGT1
CPB2
PTEN
MRTFA
SPARC
RAD51
IL17F
ABCG2
VPS11
KARS1
MIR203A
ATPLS
CSF3R
CD40
AP1B1
ITPA
CDK4
LTA
AP3B1
GALC
PARN
OCLN
PLCG2
LCK
MIR146A
ASXL1
KIF15
CD274
JMJD1C
PCCB
PROS1
ARHGEF1

MGMT
NLRP3
IRF4
PML
MCFD2
MIR210
BRIP1
EPOR
DYNC2LI1
PIK3C2A
MMP9
ANGPT2
ANGPT1
TRIM21
ZBTB16
INSL6
RARA
MAD2L2
UBE2T
NFE2
LAT
SRP72
MVK
SEC24C
HIRA
RREB1
CDC40
ARVCF
UFD1
SIRT1
BCL2
CD44
LOC117038795
HCK
MIR106B
MIR181A2
KMT2D
FANCL
ARID5B
TCIRG1
IRF2BP2
DHFR
RTEL1-TNFRSF6B
UQCRFS1

FARS2
SCARF2
RSPH14
FANCI
FANCM
HAX1
RAD51C
FGR
PFAS
STX11
MIRLET7C
WRAP53
GZMB
IL6ST
DNMT3B
SPP1
GPR89A
GPR89B
ZNF627
JAK3
RPL5
FWS
SLC35A1
USB1
GNAZ
EIF4A3
TBX6
UPF2
POLR3C
UPF3A
CARHSP1
RAB36
C16orf72
LINC01699
TBL1XR1
HPSE
TNFRSF1A
HRAS
EDN1
SH2B3
TPMT
CCND1
RAG1
SRP19

SRPRA
MIR16-1
DEL22Q11.2
TBX21
CD86
PIGA
AANAT
ASMT
HIF1A
TNFRSF8
TBXAS1
HPS5
PSMB8
GNA14
VPS45
HBG1
IL9
RPS14
C4B
RBSN
MIR34A
MIR19A
AOPEP
FANCD2OS
LOC107303338
BDPLT9
SCARB2
KDM6A
PALB2
SLC46A1
POU2F3
ALOX12
FMN1
MTOR
PDGFRB
NPC1
HBB
ERCC4
XRCC2
RFWD3
ELANE
HBG2
FCGRT
NIPBL

UNC13D
VRK2
CRBN
RPL15
FGG
TECTA
PRKAR1A
LARS2
NUMA1
BCOR
NABP1
HELLPAR
BCHE
PTPRC
LOC107980440
DPYD
COL5A1
PIK3CD
SH2D1A
MYH11
TET2-AS1
RPL11
PSAP
XIAP
MYH14
MYO7A
LIPA
ABL1
GIMAP5
TTC7A
MIRLET7D
MIRLET7A3
FCGR3B
SOS1
LOC107963955
DGUOK
RPS10
RPS24
RFX5
RFXAP
TPM4
CLCN7
MYD88
TCN2

FLT1
AGGF1
SLC35D3
SIRPA
SRSF2
MIR25
SMARCAL1
PDGFB
COL4A4
ABCA1
C4A
NOTCH1
RPS20
IRAK1
TYK2
SARM1
PIGM
PIGW
HSCB
MAL
ZNF276
ZNFX1
MIR143
MIR29A
MIR93
GIMAP1-GIMAP5
LARS2-AS1
LOC112486223
LOC112486220
MYO15A
BCL6
MMUT
CR1
CASP8
LOC107133510
MIR17
MYO3A
DIAPH3
TRIOBP
ESPN
PTPRQ
ACTRT2
PLPPR5
RECK

TUBB
CYP2C19
PLAU
IKZF1
IL2RA
VEGFA
DDX41
AGK
NHEJ1
CD52
MIR21
LOC106099062
DCLRE1C
NOS3
NLRC4
LMBRD1
LRRC56
MMAB
MMAA
RYR1
SLC19A2
RRAS2
MSH3
ASAH1
NEU1
ALDH2
RPS17
RPL26
RPS26
RPS29
LZTR1
RELA
TNFSF11
ADA2
COL5A2
GNB1
ERBB3
STAT4
SF3B1
MIR152
DOCK6
RPS28
IDH2
IDH1

RMND5B
LOC110806306
RAF1
EGF
ALG2
CD28
RMRP
GYPA
IRF5
F13A1
LBR
BLOC1S6
RPS27
RPL31
ARHGAP31
EOGT
TSR2
RPS15
MIR424
HLCS
ALG12
ACD
ABCD4
PLEK
C5
CD55
AKT1
NOS2
MIR27A
MT-CO1
DIPK1A
PEE1
COG1
CSF1
S100A8
TRPM7
CDKN2B
CXCR4
MIR221
CHD7
HDAC9
ATRX
IVD
DNASE1

P2RX7
POT1
RAD21
HYOU1
MADD
SMARCD2
MIR181A1HG
ALL1
ALL2
MIR23A
OCRL
PEPD
ANKRD11
MIR20A
PRDX1
BRD4
MMRN1
IRF7
SPN
TNIP1
DOCK8
CDK2
SERPINF2
TYMS
NSD1
CDKN1A
TWNK
MIR181A1
MYC
MLLT3
H2AC18
BCL2L1
B4GALT1
ADAMTSL1
SPRY2
JAK1
CTNNB1
TGM1
XRCC4
LOC110806263
BAX
CDK6
TNFSF13
MYB

ATP7B
ITGB2
TNFSF10
NUDT15
CA2
STT3B
COG4
RAB27A
MPO
MIR195
CTTN
IKZF3
RBM15
TRB
APOA1
YME1L1
FGFR3
HCFC1
CDKN3
RIT1
SHOC2
LOC110006319
RB1
CUBN
CD70
GRB2
IDO1
MIR23B
MIR150
JUN
MIR145
CDK1
SNX10
VPS33A
BTNL2
TMEM165
COG6
ESCO2
PKHD1
HDAC8
CHIT1
VAV1
HPS6
MYH10

PLCG1
IGF2R
SP140
MIR181B1
DDX58
SLC4A1
CFHR2
DEK
NR3C1
SLC25A4
MRAS
PPP1CB
SOS2
RRAS
SPRED2
A2ML1
RASA2
TKFC
DENND11
RPS10-NUDT3
LOC107303343
CD9
XDH
MIR222
MIR22
MIR18A
HLA-DRA
WT1
SLC7A7
UROS
HBB-LCR
IGHE
HPS3
MIR204
MIR320A
MIR107
FHL1
TGFB3
IL18R1
MET
PSTPIP1
DCK
CDKN1B
DLL4

UBA1
AMN
SNCA
THYN1
NKX2-5
MIR142
PMM2
CCL5
PIK3CA
PIK3R1
WASL
UBASH3B
G6PD
CARD14
IGKC
C2
GGT1
MSN
IL12A
VCAM1
COL1A1
COL1A2
GSTP1
FCN2
TBK1
TRIP10
CD59
UGT1A1
RAC1
GFI1
ARFRP1
EGFR
ZAN
WASF2
IL2RG
CDAN1
IGHG3
FN1
PKD1
PLEKHM1
B2M
PHGDH
ADH5
RBPJ

SLC20A2
RPL35
CORIN
RPL18
WARS2
NSUN2
ACAD9
MOGS
RPS15A
ATP6AP1
RPL27
PPIL1
XPR1
JAM2
POMP
RPS7
SARS2
SP110
STOX1
SC5D
OTUD5
GUCY1A1
DZIP1L
MYORG
IL21
IFNAR1
PPARG
TRAF6
RETN
FGFR1
PRODH
EIF1B
WASF1
SDC1
GGCX
VPS33B
AP3D1
TEK
APC
TNFRSF11A
ERF
MIR196A2
MIR127
MIR181C

JAM3
MIR99A
TNXA
HAVCR1
STAT5A
MIRLET7B
TRA
TNFRSF17
LMO2
TSC2
TWIST1
PKD2
MAN2B1
NAGLU
SLC37A4
COG7
ACKR1
AP4E1
CCDC115
CA3-AS1
TBXA2R
SMAD6
DOCK2
CENPF
ALOX12B
TGM6
ARL11
CEP72
TMEM107
C11orf65
MIR34B
MIR32
SNORD118
IGHV4-34
DPYD-AS1
IGHV3-21
TAM
DEL1Q21
CLLS2
CLLS3
CLLS4
CLLS5
CLLS1
MALAT1

CXCR3
NCAM1
BCL2L11
MYH2
ZIC3
TBX20
AFF2
NCK1
LCP2
ACTR2
ACTR3
BAIAP2
SNX9
WASF3
NCKIPSD
RHOD
WHAMM
WASHC1
WASH3P
WASH6P
WASF4P
WASF5P
BMI1
IRF3
IL15
CD33
RUNX3
HOXA10
GAS5
SHANK3
GALE
BMP6
NFE2L2
HSPD1
IL16
AICDA
CABIN1
PROC
PVT1
MIR130A
CD27
AKT3
HNRNPU
RHCE

ZBTB18
COX20
HEPACAM
VTN
FMO5
ACP6
OPRL1
SALL1
SALL2
GATAD2A
SALL3
ATF5
RTP1
RTP2
COL4A3
CCNC
SNAP29
SEPTIN5
SLC45A2
IDS
NBEA
LPA
HTR2A
ACHE
IL23R
CLU
PTX3
CD7
MIR182
CD247
KNG1
C1S
SERPING1
AIRE
CCL3
TIMP1
CD2
PARP1
CX3CR1
CD5
SQSTM1
KIRREL3
OPCML
NTM

NFRKB
DSCAML1
FEZ1
NRGN
ARHGAP32
SNX19
FRA10AC1
BARX2
MED9
BSX
TMEM185A
FRA11B
NOTCH3
ITGAV
DDRGK1
SEC23B
YES1
FRK
GPR65
IGHG2
IGHG4
IGKV@
IGLV@
IGKJ
IGLJ
CREBBP
BLOC1S1
MT-CYB
FZD6
TSC1
LIFR
AXIN1
ABCC9
SGSH
CISH
MECP2
SERPINF1
UBE2L3
GYPC
TIRAP
PDGFRL
CREB3L1
GLMN
P3H1

TNFSF4
TAF6
DEF6
SLC39A7
HGSNAT
JAZF1
DYNC2H1
PXK
GYPB
MTUS1
RINT1
BANK1
KIAA0319L
TMEM199
C6orf89
CPLANE1
MIR191
MIR10A
MIR122
CFAP92
MIR200B
MIR24-2
MIR181B2
MIR224
DGUOK-AS1
MTOR-AS1
MFF-DT
APOA1-AS
SLEB3
EOS
SLEB4
DBA2
KAZA1
PEE2
PEE3
SLEB12
SLEB13
SLEB14
SLEB15
SLEB5
SLEB7
SLEB8
SS3
CFSS

MDM2
MMP2
C5AR1
CSF1R
LPL
CD63
NFKBIA
PGF
IL22
IL2RB
CASP9
KLRK1
CFD
AR
ERCC6
CD38
NQO1
ERVW-1
CDA
CD163
FLT3LG
CYP3A4
KIF23
CDIN1
MAPK8
TNFRSF10A
SLFN13
SLFN12L
SLFN12
RAP1B
SLC22A1
HLA-DRB4
CD72
MIR183
IL33
C1R
FCN3
INF2
ABCC1
GGH
IL13
HLA-DQB1
CCL4
TLR5

PTPRN
PTPRN2
INSM1
INSM2
IL27
PTGS2
MIR146B
IL5
HGF
CASP1
EBI3
CD69
ITGAE
YARS1
TLR3
IL1R1
MCL1
TCF3
KLKB1
FGB
HRG
PROZ
PCID2
LOC101448202
HRG-AS1
LOC107126281
LOC119407405
MYH7B
UBR3
WRN
TCOF1
CCR5
MEFV
FGF2
FOS
APOB
SULT1A3
HSP90AA1
TKT
GSTM1
KDR
IFNAR2
INHA
MASP1

MASP2
AP2M1
COLEC11
CFP
C3AR1
RAPGEF1
FCN1
C5AR2
RBMS1
MYBPH
AGBL2
AGBL1
RBMS2
VSIG4
AGBL3
AGBL4
MVB12A
MIR125A
GZMA
TFAP2A
CPA3
NLRP12
SIGLEC5
IGHV4-38-2
PTPN6
LIF
HMGB1
TNFRSF4
EZH2
UGCG
ISG15
TERF1
HPRT1
AQP1
RBL2
SBF2
RETNLB
LCAT
PTGS1
SLC19A1
BIRC5
KIR3DL1
VNN1
LPIN2

CYP19A1
IL11RA
KDSR
ANPEP
TAL1
MTR
IVNS1ABP
ENTPD1
KDM4C
HLA-DQA1
MX1
SETD2
CD22
ITGAX
CFLAR
DNTT
STIM2
ORAI2
SYPL2
ORAI3
MIR196A1
GUSB
CMA1
IRF8
IL10RA
PRDM1
TCN1
KMT2E
RPL38
CCL18
PLBD2
NEAT1
CCND3
INS
RAPGEF3
IRF9
RHBDF2
KMT2B
CCR3
ERCC1
ARSA
IRF1
PPARA
NPC2

FCRL6
ENPP1
NRP1
HSPH1
IGSF3
IFRD1
ABO
ANXA2
HADHA
SCARB1
TXN
MIR185
IGF1
IFNL3
XK
ADAMTS16
NDFIP2
IRX1
ZNF84
LINC01080
ENSG00000284196
piR-39300-045
piR-31199-095
IL23A
DNMT1
PIK3CG
MMADHC
LEF1
MYCN
APAF1
PROM1
ASPH
MEIS1
PUS1
NUP107
CLDN5
PRAME
SLC25A38
MIR128-2
MIRLET7E
MIR9-1
MIR199A1
ACE
ITGB1

XRCC1
LEPR
EP300
RIPK1
POLG
RNASET2
MBP
FCER2
CD80
EIF6
MT-ND3
UTP4
MIR15B
MT-TF
KIR2DS2
PTTG1
WAC
LSM14A
MIR409
ERCC2
IGF1R
FADD
BIRC3
AURKB
IL12B
OSM
MAPK3
ADIPOQ
CCR7
LAMP1
RSAD2
RPL6
FPGS
VDR
LAIR1
CXCL16
CP
CYP2C9
HFE
SERPINA3
CD177
SMARCA4
NOD2
IL37

HDAC1
IFI27
CYP2D6
IDUA
TREML1
SMC3
MSH2
ARF1
FOXP2
CDC45
TNFRSF9
RECQL4
MPV17
TNFRSF18
USH1C
RPS16
CXCR5
RPS25
MT-ATP6
MIR30E
CDKN2B-AS1
MIR130B
CHEK1
LCN2
ATR
CTSD
NF1
GATA4
COL2A1
CREB1
PLK1
GLA
SPTAN1
FGF8
TP53BP1
DDX11
BGLAP
VTI1B
EMG1
STAP1
PNPLA5
CPLX3
DCLRE1B
MEG3

SLC66A1
TLDC2
HOTAIR
MIR125B1
CCND2
ITPR3
CASQ1
RTN4R
ERP44
NUP133
ESYT1
MICU2
JSRP1
TMCC1
EGR4
TMEM178A
SARAF
CRACR2A
STIMATE
MIR151A
TNFRSF1B
XRCC6
XRCC5
DNASE1L3
IL21R
MRE11
TERF2
HLA-C
GSR
ABCC2
AFP
MYO18A
SMC1A
SIRT2
SIRT3
G3BP1
LOC102724971
TNFRSF6B
IFIT3
SST
CST3
PRKDC
IL6R
ASS1

SLC19A3
IL12RB1
HAMP
PLAUR
RAD17
MDC1
URB2
H19
MME
ABCC3
ZRSR2
TALDO1P1
IGH
TNFRSF10B
DRD2
MUC1
RXRA
SCN8A
ALOX5
CYP1A1
LEP
CDKN1C
KAT5
SMARCB1
PKLR
POLR1D
RNASEL
CCNA2
EXO1
NUP98
CEBPB
LDLRAP1
MTMR3
PLD3
MSH5
ASAH2
CHD8
RPL3
MRC1
CCL19
BHLHE40
PLA2R1
ID4
RPL29

PLD4
RSL24D1
PIF1
LGALS13
KCTD13
DGKK
PLD5
HORMAD2
MBOAT2
NMD3
CLEC5A
NPB
TENT4A
TUG1
XIST
UCA1
DGCR5
MIR30A
MIR134
MIR486-1
TF
CDKN2A
ITGA5
RRM2B
CD79B
ACTA2
NCOR2
PTPN3
ALAS2
BCS1L
FOLR2
FAF1
RIPK3
TRIP11
DTNBP1
THY1
YARS2
ARFGAP1
MLKL
MVP
SFXN4
ARFGAP3
GLRX5
PVALB

NUP160
NUP85
TRMU
VPREB1
HOXA9
ANKRD49
PIR
RAB3IL1
AHCTF1
ARFGAP2
MT-ND4
MT-CO2
MGME1
MT-ND6
MT-CO3
CCDC12
MIR140
MIR199B
MIR451A
MT-TL1
MT-TH
MT-TK
MT-TG
PDPN
IFNGR1
VIM
OTC
ELN
MCEE
ARID2
MPEG1
CNR2
SOCS3
MTRR
TYMP
SLCO1B3
MAPK14
RAD50
GAPDH
SERPINA1
COL4A1
BLM
PON1
HSPA5

TUBG1
ADSL
HLA-DPB1
HNF1A
ETFA
ACADVL
CTSG
RPA1
M6PR
RNF168
IFI16
FBL
RPS13
AIM2
IFITM3
PIGB
HUS1
GP2
IFITM1
PIGT
GTPBP1
GAR1
FCGBP
H2AX
MARVELD3
NAT16
KLK3
MIF
ABCC4
DPP4
ACADS
ALK
MAVS
IGHM
VIPR1
MBTPS2
IL3RA
MAGOH
RAC2
NOTCH2
SLC6A3
NRXN1
PI4KA
NRG1

BARD1
SLC25A1
TXNRD2
GJA5
TP73
CHRNA7
BLNK
NCOR1
FOXP1
HLA-G
TUBA8
ANK3
DISC1
FMR1
FHIT
KMT2C
KAT6A
RANBP1
SPI1
AP2S1
P2RX6
SUZ12
CLTCL1
DGCR8
GCM2
HMGN1
HOXA7
NLGN4X
CD58
TOP3B
DGCR2
BBC3
CYFIP1
MED15
CD68
RUNX1T1
SF3B2
TRMT2A
SPECC1L
ZDHHC8
KLHL22
KIR2DL3
NIP7
FOXE3

MRPL40
PHF21B
PSMA8
STK32C
DHX29
AIFM3
ZNF74
FUT4
GNB1L
MRPS18A
RARS1
BAALC
AHDC1
PPP1R18
ZNF804A
MZB1
TANGO2
CD24
PRAM1
PRDM4
MRTO4
RPF1
THAP7
LSG1
DGCR6
DGCR6L
GLIPR1L2
ANKRD37
VWA7
WBP1L
RABL2B
DYNLT2B
CNPY1
FOXI3
H2AC16
H2AC17
C22orf15
OR2T12
PLPPR1
MBD3L3
MIR26B
RIMBP3B
MIR149
MIR342

HOTAIRM1
LRRC74B
MIR483
MIR193A
MIR574
MIR423
MIR30D
MIR1306
MIR376A1
MIR374A
CCAT1
MIR147A
RNASEH2B-AS1
FAM230B
ADAM10
KEAP1
SEMA4D
RORC
BAD
CARD9
MBD4
IL18BP
MBD2
IFNL2
IFNL1
KIR2DL2
KIR2DS5
THRIL
MIR148A
CDH1
AURKA
SET
EGR1
FSCN1
CD1A
FAAP100
FURIN
AXL
MSH6
RARB
PTCH1
TNFRSF11B
CASP7
LMNA

LRRK2
SLC12A6
FES
ABCB4
TLR8
CPS1
HEXA
PTK2
CARD11
PC
VKORC1
NPPA
HERC2
ABCA4
SGPL1
DAG1
B4GALNT1
ACADSB
ADM
RPSA
UGT8
CSF2RA
DKK1
ASPA
B3GAT1
CYP26A1
MBTPS1
ARAF
LAMP2
NLRP1
BGN
RPA2
NOP56
GDF15
CORO1A
GM2A
INPP4A
MUS81
CCL11
CXCR1
BCKDHB
ATP13A2
RNASEH1
PABPC1

PHF8
NAGS
FLVCR1
MSLN
RPS27A
UBA7
CBX4
AP4B1
ID1
PROCR
TSG101
GBA2
OAS3
RNASE3
AZIN1
MARVELD2
PUS7
PAPPA2
CD83
SLAMF1
HLTF
CD99
POLM
UNC93B1
RCHY1
PIGL
DNASE2
LARP1
UGT3A1
BST2
MAGEA1
PRKN
DLX1
FAN1
HELQ
GULP1
IFITM2
TRUB1
SYT6
SRA1
MAGEA3
MAGEA4
RASIP1
CDCA5

PDS5B
SPCS1
SPRTN
DPH7
PIGY
ACTRT3
SHQ1
TRUB2
CREG2
PCP2
CTAG1B
IGSF5
URB1
XKR7
FAAP24
WT1-AS
MIR33A
MIR214
MIR615
MIR324
HOXA-AS2
H3-7
SNORA67
MIR500A
SCARNA23
FAM223A
MIR1288
MT-TN
MT-TS2
MT-TM
MT-TD
LOC110806264
LOC122056785
HSPA4
SON
PRKCQ
PLA2G7
MAD2L1
DYSF
TIMP2
SYNE1
ITGA4
PTHLH
BIRC2

MIR335
FLT4
RHOA
EFTUD2
CXCL13
CXCL9
SLC35C1
MALT1
CRLF2
TENT5A
IFNGR2
SOD1
ACE2
HSPG2
TYRO3
GJB6
BCL2L2
HLA-S
NLRP6
BMS1P20
IL4R
NR1I2
ADH1B
F12
SLC29A1
LILRA3
ST3GAL4
KLF12
SNX11
MIR557
NEWENTRY
ASPG
ELOVL4
TPO
C1QA
TRIM25
STAG2
CHRNE
CLTA
IFNA13
PAEP
NAAA
MTX1
CAT

PRB2
COX8A
FLII
FGF4
SLURP1
KRT20
BTG3
RIEG2
CDC20
C20orf181
SH2B2
MT1E
GBAP1
MXI1
DCPS
FUNDC2
GGTLC5P
DERL1
PRDX2
SOAT1
SLC25A10
CYLD
CYP2C8
RO60
MFT2
PWAR1
OR2AG1
DDX53
SRF
GSC
CSN1S1
SESTD1
SLCO1B1
PRSS27
SLC28A1
RN7SL263P
GGTLC1
RNF34
LOC102724197
APBB3
S1PR4
MYL9
CHRD
CEACAM5

BCL10
SPZ1
LINC01152
GGTLC4P
GGT2
GGTLC3
GTF2IRD1
HSP90B1
CRISP2
PRDX6
LINC-ROR
ECT
TGS1
NCR1
XPO1
CIT
SLC52A2
DDIT3
WDHD1
CACNB3
ZFP36
PADI4
MTHFD1
MSR1
MPI
ASGR1
GEM
SMCP
MCC
CBLIF
NOS1
SH3BP4
CLEC1B
IL4I1
DUOX2
C20orf194
PRPF31
GAS6
ACAD8
CXCL1
CXCL2
GTF2I
HTC2
HMOX1

HLA-DQB2
HLA-DPA1
BTBD8
DNM2
PVR
STX2
WDR18
C1GALT1
SPHK2
PRSS55
MMS19
ATN1
XYLT2
TNMD
SCT
E2F1
EFNA5
RRM1
ELF3
EMP1
PNLIP
DUOX1
ATP7A
NT5C2
KDM1A
PIK3CB
FOLH1
RASA3
FHL3
PRKG1
ZC4H2
ARHGEF40
RNPC3
PTPA
FBN1
MOCOS
FCER1G
FGFR2
POU2F1
RHOF
TREM1
TRIM33
G6B
IFZF5
