# Supplementary material for: Identifying and Targeting Prediction of the PI3K-AKT Signaling Pathway in Drug-Induced Thrombocytopenia in Infected Patients Receiving Linezolid Therapy: A Network Pharmacology-Based Analysis
Source: J Healthc Eng. 2022 Oct 15;2022:2282351. doi: 10.1155/2022/2282351 (PMC9588367; doi:10.1155/2022/2282351)
Supplement: Supplementary Materials — Supplementary Table 1 and experimental dataset are provided for MCODE cluster analysis. Supplementary data files for all the figures are also provided in the supplementary materials. [file 2282351.f1.zip › Figures 1-3 drug data.pdf]

MAOA
LACTB
MAOB
CYP3A4
CYP2D6
CYP2C9
CYP1A2
F5
POLG
TYMP
SCO2
EDAR
ALB
IL6
SLC22A6
SLC22A8
CFTR
MT-CO2
PIK3C2A
CYP3A5
CYP2C19
CYP2E1
RPL4
IFNG
CCL2
IL10
PDF
RRM2B
SLC25A4
GHR
DGUOK
FANCI
MPV17
POLG2
SSBP1
TMEM70
NT5M
UPP2
TEFM
TWNK
MT-TT
MT-TM
PPBP
NLRP3

MT-TL1
MT-TK
GHRL
CLPB
MT-ND5
MT-TF
CAT
FASN
HSPD1
EPO
MT-CO1
MT-ND6
MT-ND1
MT-CO3
MT-CYB
MT-TS1
MT-TV
MT-TI
MT-TS2
MT-TW
MT-TQ
MT-TC
MT-TP
F10
STAT3
GBA
MAPK14
NAMPT
PRKDC
RORC
KDR
MMP13
RPS6KA3
RPS6KA2
PDE10A
PIK3CB
HPGDS
SMO
CHEK1
P2RX7
SYK
MET
TNKS2
LRRK2

RAF1
JAK2
PARP1
PDE9A
ASAH1
CNR1
CNR2
PIK3CD
PIK3CG
PIK3CA
TNIK
MELK
GABRA5
ALOX5
PDE1C
AVPR2
PLAT
AVPR1A
KLKB1
NTRK1
GRM5
MAPK1
CD38
TBK1
SMG1
CASP6
CASP7
GCK
AKT1
CASP1
MCHR1
TSPO
PABPC1
FLT1
MAP3K12
FGFR1
ADORA1
ADORA2A
ADORA3
IRAK4
CCNE1
FLT3
CDK9
TDO2

ACHE
ELANE
GSK3B
CDK2
ADK
IDO1
PDE3A
PDE3B
CA12
CAPN1
HSD11B1
EPHB4
ERBB2
EGFR
MTOR
CCR8
PTK6
MAP3K8
SLC6A9
AURKB
CDK1
CDK4
LIPE
NAAA
MMP12
AURKA
ZAP70
PDE1A
SELE
PDE1B
KIT
SCN5A
MAPK8
CCNT1
ACACB
AKR1C3
CYP2J2
COPS5
FADS1
GRM2
HKDC1
SLC16A3
NPY2R
PIK3C2G

PDE8B
RAD1
RAD51
SLC6A5
SIGMAR1
STAMBP
STAT6
